# Supplementary material for: Myddosome clustering in IL‐1 receptor signaling regulates the formation of an NF‐kB activating signalosome
Source: EMBO Rep. 2023 Aug 21;24(10):e57233. doi: 10.15252/embr.202357233 (PMC10561168; doi:10.15252/embr.202357233)
Supplement: Supplementary file 1 — Appendix S1 [file EMBR-24-e57233-s004.pdf]

Appendix for **Myddosome clustering in IL-1 receptor signaling regulates the formation of an NF-kB activating signalosome**

Fakun Cao, Rafael Deliz-Aguirre, Fenja H. U. Gerpott, Elke Ziska, and Marcus J. Taylor*

Max Planck Institute for Infection Biology, Chariteplatz 1, Berlin D-10117 Germany

* Corresponding author: taylor@mpiib-berlin.mpg.de

Table of Contents:

Appendix Figure S1: page 2

Appendix Figure S2: page 4

Appendix Figure S3: page 6

Appendix Figure S4: page 8

Appendix Figure S5: page 9

Appendix Figure S1

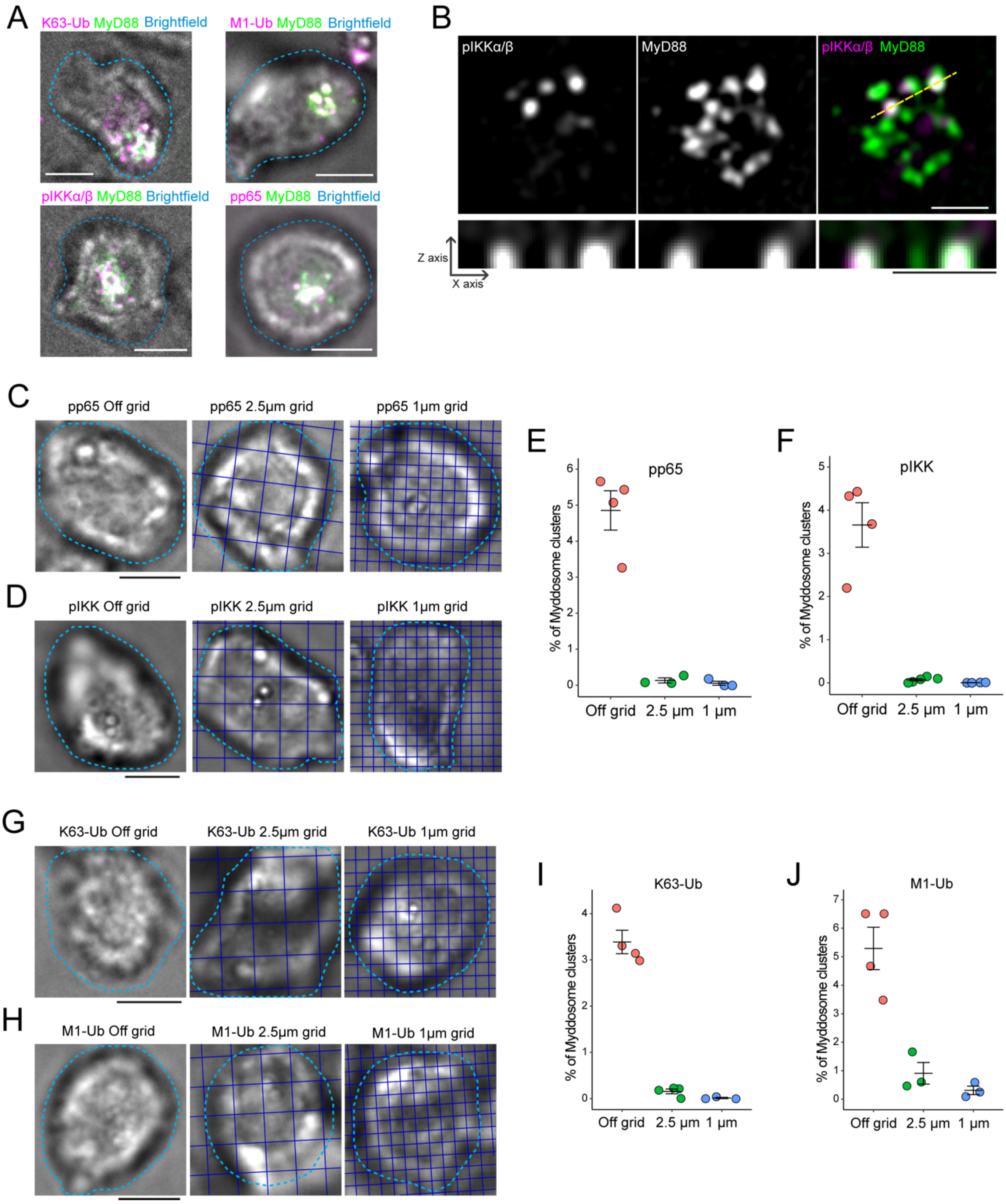

Appendix Figure S1: Additional immunofluorescence images and analysis

A. Brightfield images from Fig. 2A. Brightfield images overlaid with cell contour (blue dashed line) and overlaid with MyD88-GFP and antibody staining. Scale bar, 5 µm.

B. Additional SIM images showing colocalization between Myddosome and pIKKa/b. Scale bar, 1 µm.

(C and D) Brightfield images for Fig. 3A and 3C (A), and Fig. 3B and 3D (B). Cyan dashed lines are cell contour and blue lines indicate grid lines. Scale bar, 5 µm.

(E and F) Quantification of the percentage of MyD88 puncta classified as Myddosome clusters in pp65 (E) or pIKK (F) immunofluorescence staining off grids, on 2.5 µm and on 1

μm grids. MyD88 puncta with an integrated intensity greater than or equal to 0.5 are classified as Myddosome clusters. The percentage of Myddosome clusters off grids, on 2.5 μm and on 1 μm grids in pp65 staining are 4.853 ± 0.547%, 0.133 ± 0.072% and 0.055 ± 0.055%, respectively; and that in pIKK staining are 3.657 ± 0.515%, 0.071 ± 0.026% and 0.004 ± 0.003%, respectively. Bars represent mean ± SEM (n = 3 - 4 biological replicates for pp65, with 259 - 9342 puncta measured per replicate, and 10273, 14009 and 2675 puncta measured off grids, on 2.5 μm and 1 μm grids, respectively; n = 4 - 5 biological replicates for pIKK, with 435 - 24998 puncta measured per replicate, and 2375, 35496 and 59593 puncta measured off grids, on 2.5 μm and 1 μm grids, respectively)
(G and H) Brightfield images for Fig. 4A and 4C (A), and Fig. 4B and 4D (B). Cyan dashed lines are cell contour and blue lines indicate grid lines. Scale bars, 5 μm.
(I and J) Quantification of the percentage of MyD88 puncta classified as Myddosome clusters in K63-Ub (C) or M1-Ub (D) immunofluorescence staining off grids, on 2.5 μm and on 1 μm grids. MyD88 assemblies with an integrated intensity greater than or equal to 0.5 are classified as large MyD88 assemblies. The percentage of large MyD88 assemblies off grids, on 2.5 μm and on 1 μm grids in K63-Ub staining are 3.387 ± 0.253%, 0.156 ± 0.053% and 0.013 ± 0.013%, respectively; and that in M1-Ub staining are 5.292 ± 0.744%, 0.908 ± 0.378% and 0.310 ± 0.147%, respectively. Bars represent mean ± SEM (n = 3 - 4 biological replicates for K63-Ub, with 484 - 14628 puncta measured per replicate, and 14571, 27494 and 24026 puncta measured off grids, on 2.5 μm and 1 μm grids, respectively; n = 3 - 4 biological replicates for M1-Ub, with 338 - 3887 puncta measured per replicate, and 3114, 6091 and 1844 puncta measured off grids, on 2.5 μm and 1 μm grids, respectively).

Appendix Figure S2

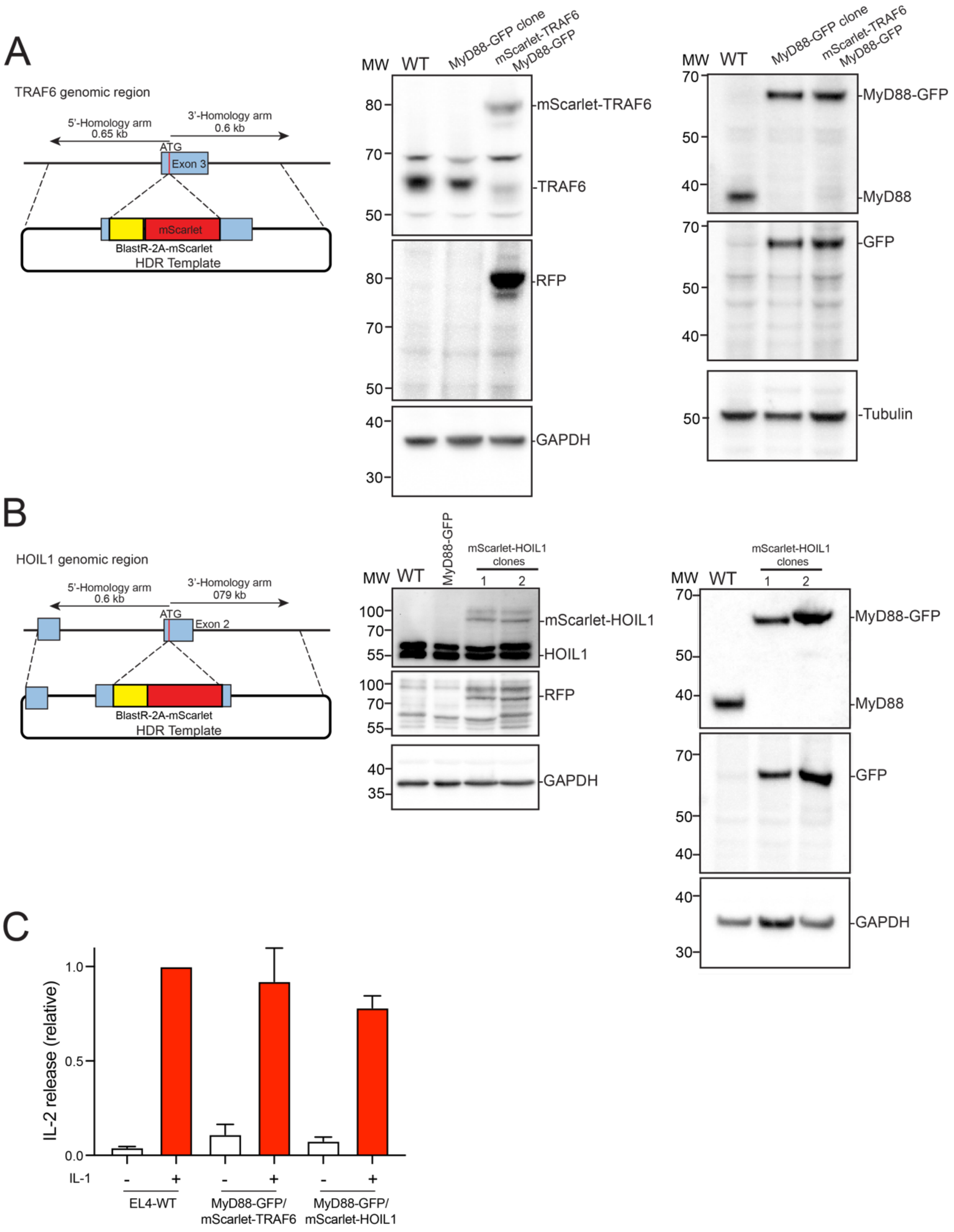

Appendix Figure S2. Validation of EL4 cell lines with CRISPR/Cas9 gene edited MyD88-GFP and mScarlet-TRAF6/HOIL1.

(A and B) Left, schematic of the TRAF6 (A) or HOIL1 (B) gene locus and HDR template designed to insert a mScarlet open reading frame immediately after the start codon. EL4 cells were electroporated with HDR and gRNA/Cas9 plasmids to simultaneously edit MyD88 and TRAF6 (A) or MyD88 and HOIL1 (B) gene loci. Middle, western blot analysis of mScarlet-TRAF6 (A) or HOIL1 (B) in dual gene-edited EL4 clones. Western blots of whole cell

lysates were probed with anti-TRAF6/HOIL1 and anti-RFP antibodies to confirm editing and insertion of fluorescent protein open reading frames at both gene loci. WT EL4 cells and EL4 cells expressing MyD88-GFP only served as negative controls. Right, western blot analysis of MyD88-GFP in dual gene-edited EL4 clones. Western blots of same lysates were probed with anti-MyD88 and anti-GFP to confirm editing and insertion of GFP open reading frame at the MyD88 gene locus. WT EL4 cells served as a negative control and EL4 cells expressing MyD88-GFP only served as a positive control. For MyD88-GFP/mScarlet-HOIL1 cells, all data presented were acquired with clone 1.
(C) IL-2 release in WT and dual gene-edited EL4 cells. IL-2 release was measured by ELISA 24 h after IL-1β stimulation. Values for gene-edited cells shown relative to EL4 WT. Average values are calculated from 3-4 independent experiments. Bars represent mean ± SEM.

Appendix Figure S3

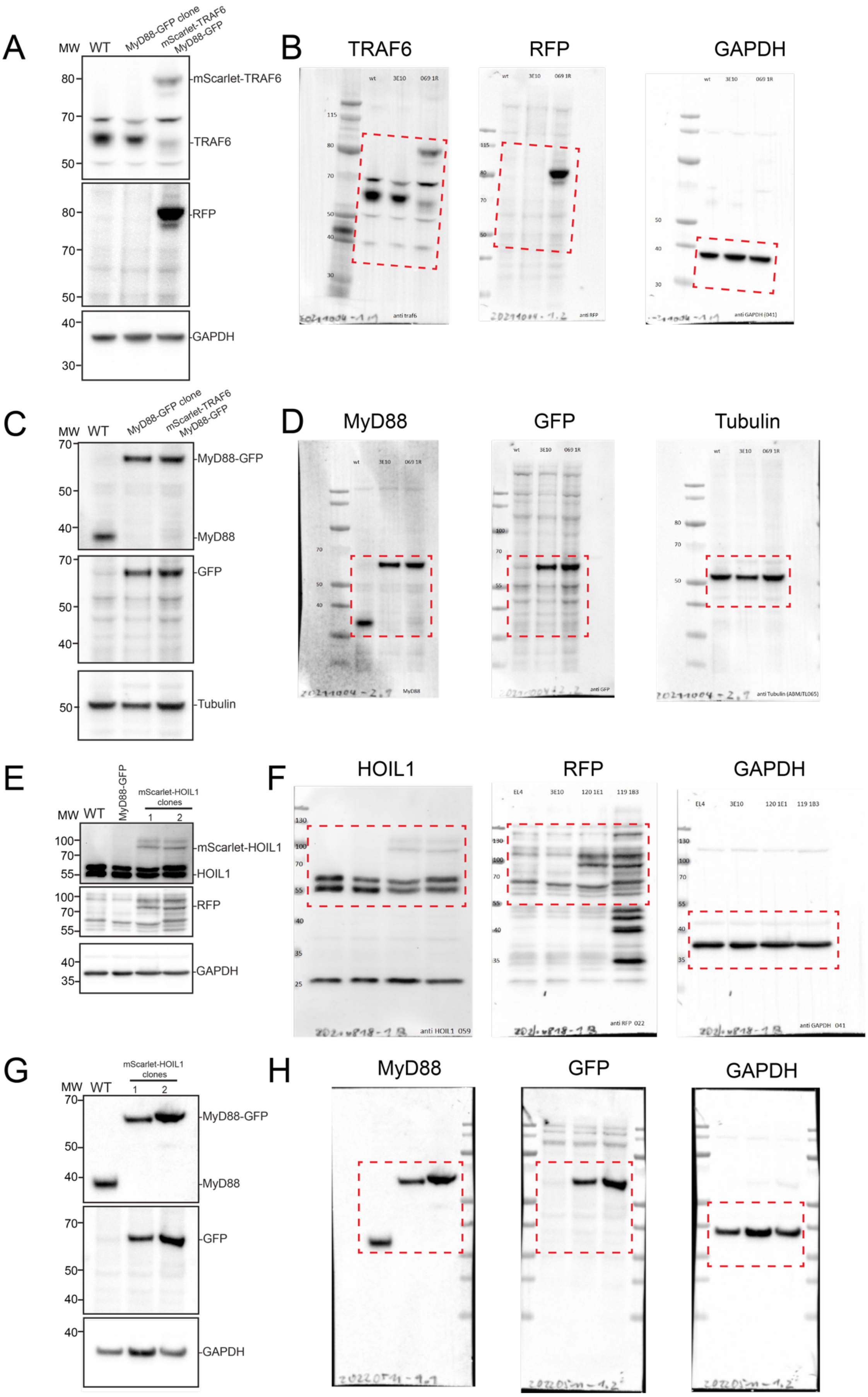

Appendix Figure S3. Full western blots.
A) Cropped blots from Appendix Figure S2A (middle panel)
B). Full western blots of MyD88-GFP/mScarlet-TRAF6 cell line for Appendix Figure S2A (middle panel).
C) The cropped blot from Appendix Figure S2A (right panel)
D) Full western blots of MyD88-GFP/mScarlet-TRAF6 cell line for Appendix Figure S2A (right panel).
E). Cropped blots from Appendix Figure S2B (middle panel)
F) Full western blots of MyD88-GFP/mScarlet-HOIL1 cell line for Appendix S2B (middle panel).
G) Cropped blots from Appendix Figure S2B (right panel)
H) Full western blots of MyD88-GFP/mScarlet-HOIL1 cell line for Appendix S2B (right panel).
All cropped blots are the same as those shown in Appendix Figure S2. Cropped area indicated with red box overlaid the full length blots.

Appendix Figure S4

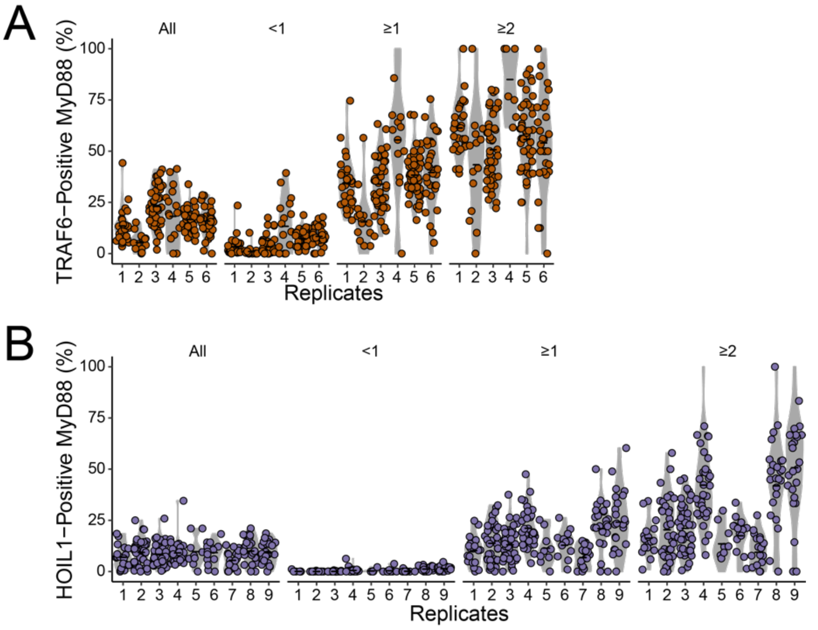

Appendix Figure S4. Individual replicates of mScarlet-TRAF6 and mScarlet-HOIL1 recruitment to single Myddosomes and Myddosome clusters.

A. Percentage (%) of MyD88-GFP puncta that colocalizes with TRAF6 per cell for all puncta, and puncta categorized as containing <1, ≥1, or ≥2 Myddosome complexes, for individual biological replicates shown in Fig. 5C. Violin plots indicate the distribution of individual cell measurements. Colored dots superimposed on the violin plots are the averages from individual cell measurements (n = cells for replicates 1-6: n = 29,17,45,17,48 and 35). Bar represents mean. In total a minority of MyD88 puncta recruit TRAF6 ("All", replicates 1-6: 13.5%, 5.3%, 23.0%, 18.1%, 16.7% and 15.9%). The percentage of TRAF6 recruitment was low for MyD88 puncta containing <1 Myddosome complex ("<1", replicates 1-6: 3.3%, 1.0%, 3.3%, 13.3%, 7.3% and 8.4%), but increased with the number of complexes per puncta ("≥1", replicates 1-6: 34.3%, 15.5%, 33.9%, 55.5%, 38.3% and 39.6%; "≥2", replicates 1-6: 61.3%, 43.4%, 50.7%, 85.0%, 55.7% and 54.0%).

B. Percentage (%) of MyD88-GFP puncta that colocalizes with HOIL1 per cell for all puncta, and puncta categorized as containing <1, ≥1, or ≥2 Myddosome complexes, for individual biological replicates shown in Fig. 5C. Violin plots indicate the distribution of individual cell measurements. Colored dots superimposed on the violin plots are the averages from individual cell measurements (n = cells for replicates 1-9: n = 19, 35, 46, 34, 9, 15, 21, 27 and 24). Bar represents mean. In total a minority of MyD88 puncta recruit HOIL1 ("All", replicates 1-6: 7.0%, 9.3%, 8.4%, 9.1%, 9.7%, 9.2%, 5.5%, 10.0% and 9.2%). The percentage of HOIL1 recruitment was low for MyD88 puncta containing <1 Myddosome complex ("<1", replicates 1-6: 0.06%, 0.01%, 0.04%, 0.52%, 0.26%, 0.34%, 0.11%, 1.2% and 1.4%), but increased with the number of complexes per puncta ("≥1", replicates 1-6: 10.2%, 13.8%, 13.0%, 21.1%, 11.4%, 12.8%, 6.8%, 20.9% and 24.1%; "≥2", replicates 1-6: 16.2%, 20.4%, 21.1%, 42.2%, 13.5%, 17.4%, 9.4%, 41.9% and 50.7%).

Appendix Figure S5

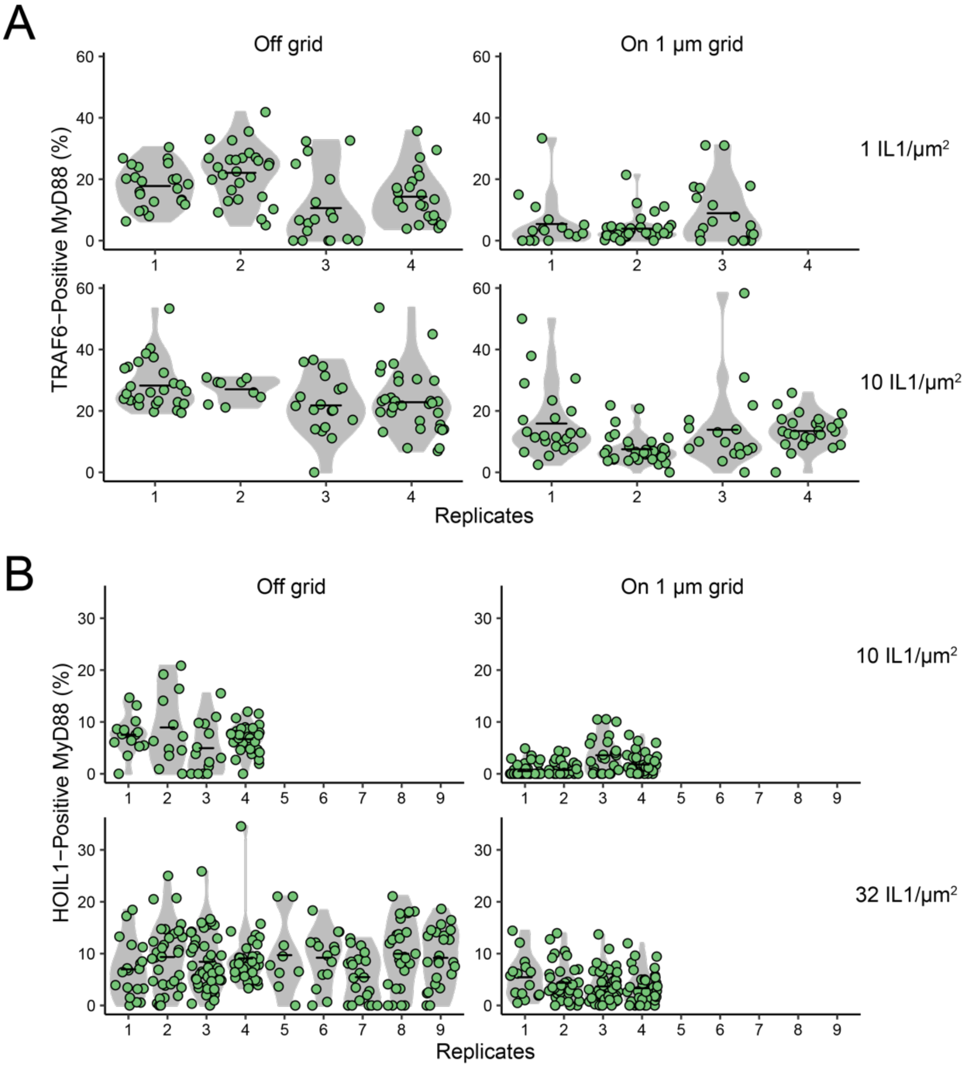

Appendix Figure S5: Biological replicate data from the characterization of dynamics of MyD88-GFP/mScarlet-TRAF6 cells off grids and on 1 µm grids.

A. Percentage of MyD88-GFP puncta that colocalizes with TRAF6 off grids and on 1 µm grids at 1 or 10 IL1/µm^2 per cell across biological replicates shown in Fig. 6C and 6F. Violin plots indicate the distribution of individual cell measurements. Colored dots superimposed on the violin plots are the averages from individual cell measurements. Bars represent means.

B. Percentage of MyD88-GFP puncta that colocalizes with TRAF6 off grids and on 1 µm grids at 1 or 10 IL1/µm^2 per cell across biological replicates in Fig. 6I and 6L. Violin plots indicate the distribution of individual cell measurements. Colored dots superimposed on the violin plots are the averages from individual cell measurements. Bars represent means.
