# Supplementary material for: Myddosome clustering in IL‐1 receptor signaling regulates the formation of an NF‐kB activating signalosome
Source: EMBO Rep. 2023 Aug 21;24(10):e57233. doi: 10.15252/embr.202357233 (PMC10561168; doi:10.15252/embr.202357233)
Supplement: Supplementary file 2 — Expanded View Figures PDF [file EMBR-24-e57233-s013.pdf]

Expanded View Figures

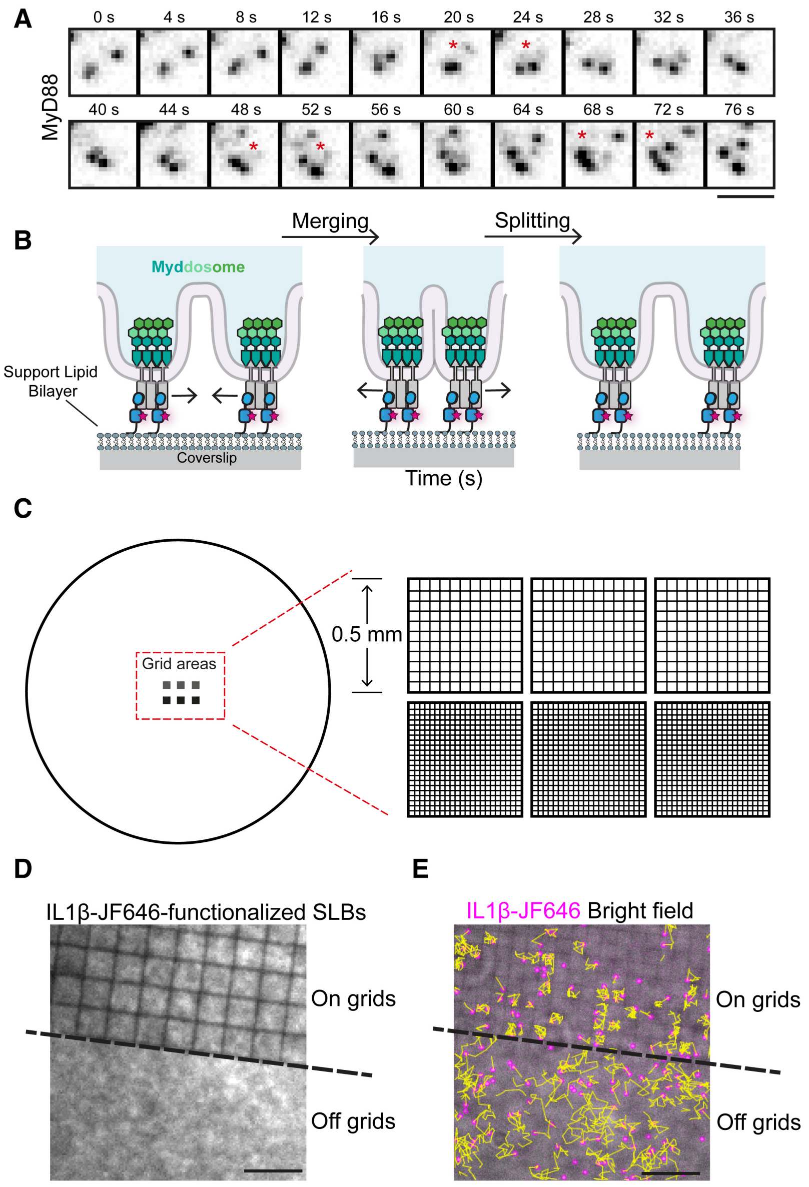

Figure EV1. Illustrations of nanopatterned coverslips to assemble partitioned SLBs.

A Montage of TIRF images showing examples of Myddosomes merging to form clusters and Myddosomes splitting into small puncta. Asterisks indicate frames of merging and splitting. Scale bar, 2 μm.

B Schematic of an alternative membrane topology of Myddosome clusters than the membrane topology presented in Fig 1B.

C Schematic of the coverslip used for preparing partitioned SLBs. The no. 1.5 coverslips with a diameter of 25 mm were fabricated with nanopatterned chromium grids containing square corrals with 2.5 or 1 μm^2 dimensions. Each dimension contains three pieces of square grids with a side length of 0.5 mm.

D TIRF image showing IL-1-JF646 functionalized SLBs at a field of view containing on grids and off 2.5 μm grids SLBs. Continuous SLBs are observed off grids and on each square corral on grids. Scale bar, 5 μm.

E Mobility of IL-1 ligands off and on 2.5 μm grids. Yellow lines are trajectories of IL-1-JF646. Off grids, IL-1 can freely move around, while on grids, IL-1 can only move within individual corrals. Scale bar, 10 μm.

 © 2023 The Authors

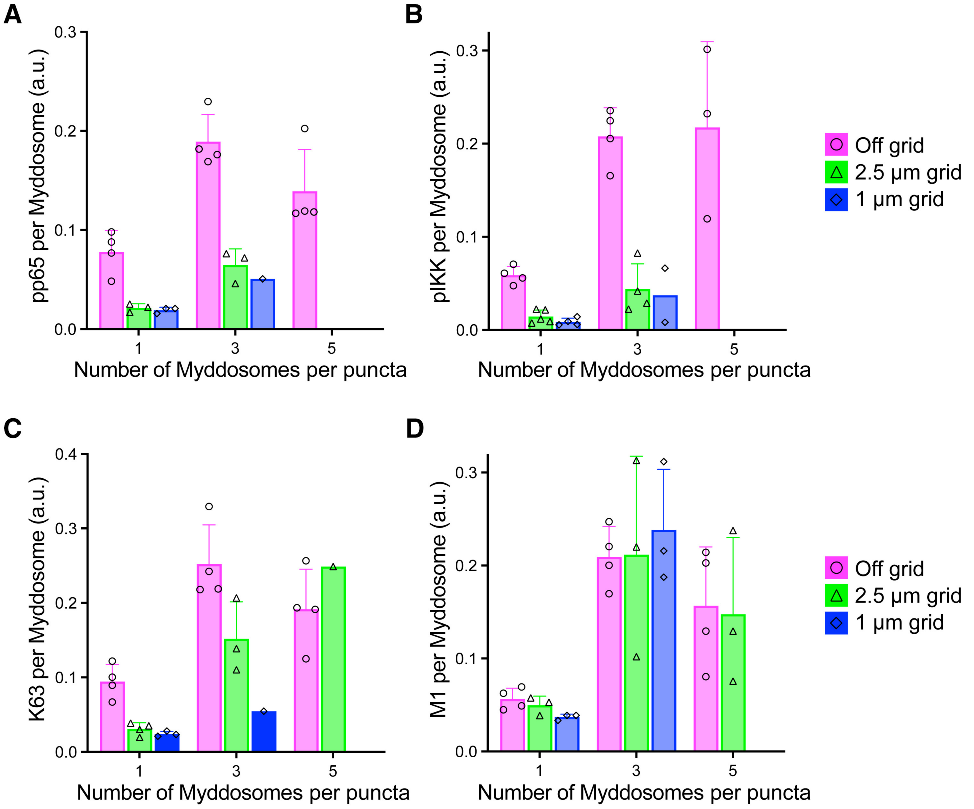

Figure EV2. Staining intensity per complex of single Myddosome and Myddosomes organized into clusters.

A–D The staining intensity for pp65, pIKK, K63-Ub, and M1-Ub (A–D, respectively) of MyD88-GFP puncta using the same datasets from Figs 3 and 4. The staining intensity is normalized to the number of Myddosome contained within the puncta. Data points represent averages from individual biological replicates (three biological replicates for off-grid cells stained for pp65, four biological replicates for off-grid cells stained for pIKK, K63-Ub, and M1-Ub, three biological replicates for 2.5 and 1 µm grids stained for pp65, pIKK, K63-Ub, and M1-Ub). Bars represent mean ± SEM.

© 2023 The Authors

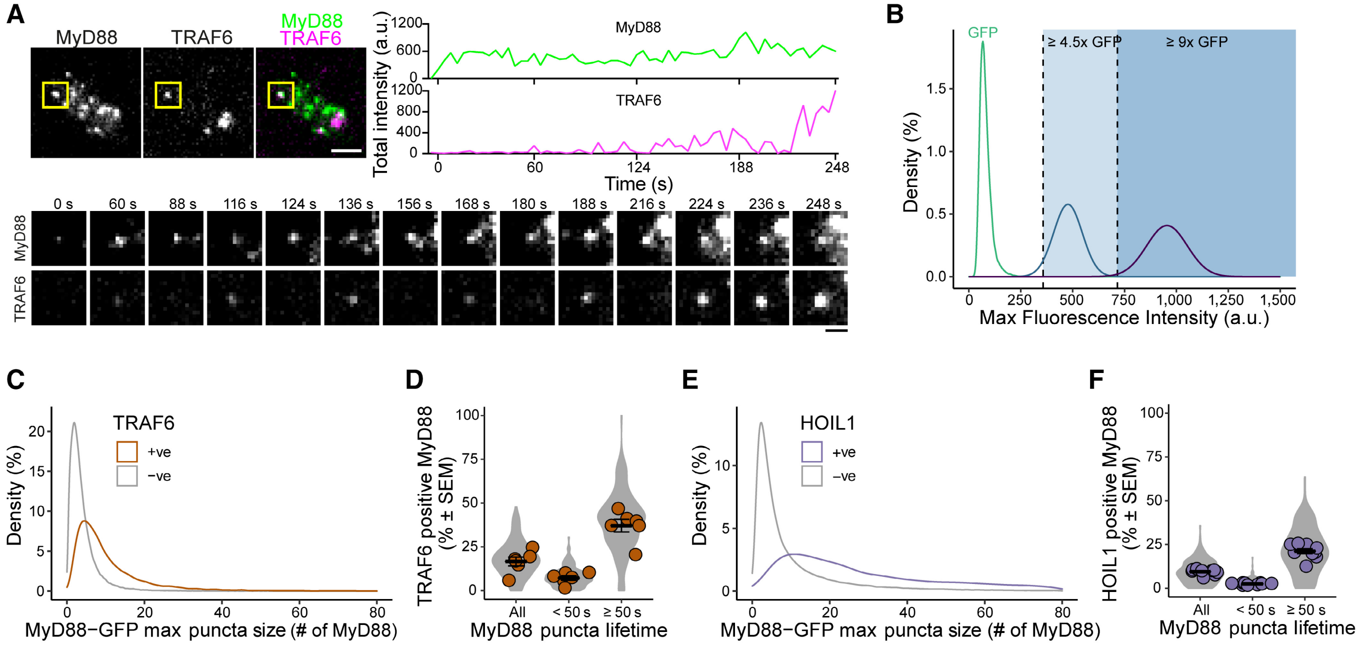

Figure EV3. Characterization of dynamics of MyD88-GFP/mScarlet-TRAF6 and MyD88-GFP/mScarlet-HOIL1 cells.

A TIRF images of an EL4 cell expressing MyD88-GFP and mScarlet-TRAF6. Scale bar, 2 μm. Time-series images indicate MyD88 and TRAF6 puncta from the yellow boxed area. Scale bar in time-series images, 1 μm. TRAF6 is transiently recruited to MyD88 until TRAF6 becomes stable and nucleates on MyD88 puncta. Fluorescence intensities of MyD88 and TRAF6 overtime are shown at top right.

B Density plot of single molecules of GFP (green, n = 40,229 GFP particles) and estimated intensity distribution of a 6× GFP multimer (blue) and a 12× GFP multimer (purple). Shaded light blue and dark blue regions designate intensity values ≥ 4.5× GFP and ≥ 9× GFP, respectively, which were used to categorize MyD88 puncta as containing ≥ 1 or ≥ 2 Myddosome complexes.

C Density plot showing the distribution of MyD88 oligomer size (number of MyD88-GFP monomers is derived from the maximum intensity divided by the average intensity of GFP) for MyD88 puncta that are positive (+ve) or negative (−ve) for TRAF6. The average size for puncta positive or negative for TRAF6 recruitment is 10.4 versus 3.9 MyD88s, measured from 13,526 positive MyD88 puncta versus 83,462 negative MyD88 puncta measured in 191 cells and combined from six biological replicates.

D Quantification of the percentage of MyD88-GFP puncta per cell that colocalizes with TRAF6 for all puncta and puncta with lifetimes < 50 s or ≥ 50 s. Violin plots show the distribution of individual cell measurements. Colored dots superimposed on violin plots correspond to the average value in the independent experiments (n = 6 biological replicates, with 17–48 cells measured per replicate). Bars represent mean ± SEM.

E Density plot showing the distribution of MyD88 oligomer size (number of MyD88-GFP monomers is derived from the maximum intensity divided by the average intensity of GFP) for MyD88 puncta that are positive (+ve) or negative (−ve) for HOIL1. The average size for puncta positive or negative for HOIL1 recruitment is 47.4 versus 11.6 MyD88s, mean calculated from 10,300 HOIL1-positive MyD88 puncta versus 108,054 negative MyD88 puncta measured from 230 cells and combined across nine biological replicates.

F Quantification of the percentage of MyD88-GFP puncta per cell that colocalizes with HOIL1 for all puncta and puncta with lifetimes < 50 s or ≥ 50 s. Violin plots show the distribution of individual cell measurements. Colored dots superimposed on violin plots correspond to the average value in the independent experiments (n = 9 biological replicates, with 9–46 cells measured per replicate). Bars represent mean ± SEM.

© 2023 The Authors

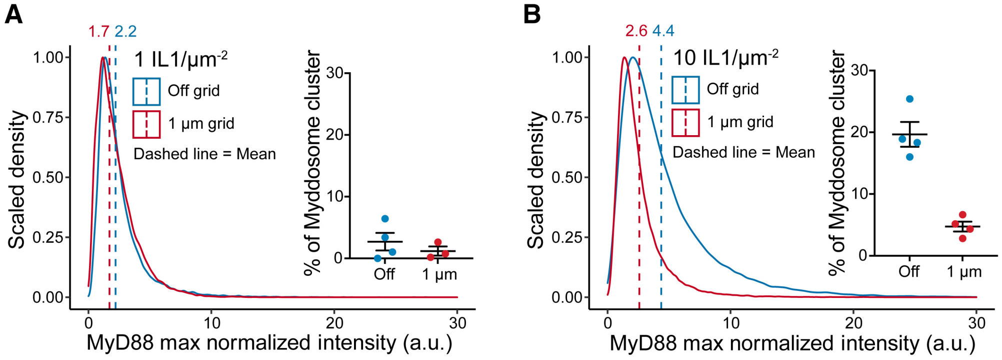

Figure EV4. Characterization of dynamics of MyD88-GFP puncta size in MyD88-GFP/mScarlet-TRAF6 cells off grids and on 1 μm grids.

A, B Scaled density distribution of MyD88 max normalized intensity off grids and on 1 μm grids at a ligand density of 1 (A) or 10 (B) IL1/μm^2. The average MyD88 max normalized intensity (dashed line) at 1 IL1/μm^2 off grids versus on 1 μm grids are 2.2 versus 1.7, and at 10 IL1/μm^2 are 4.4 versus 2.6. Insets are quantifications of the percentages of Myddosome clusters. A Myddosome cluster is defined as a MyD88-GFP puncta containing equal to or greater than two Myddosomes. At 1 IL-1/μm^2, the percentages of Myddosome clusters off grids versus on 1 μm grids are 2.7 ± 1.4% versus 1.2 ± 0.7% and at 10 IL1/μm^2 are 19.7 ± 2.0% versus 4.7 ± 0.8%. Bars represent mean ± SEM. At 1 IL1/μm^2, data are measured from 24,315 MyD88 puncta off grids from 91 cells and four replicates, and 23,161 MyD88 puncta on 1 μm grids from 70 cells and three replicates. At 10 IL1/μm^2, data are measured from 34,452 MyD88 puncta off grids from 87 cells and four biological replicates, and 71,525 MyD88 puncta on 1 μm grids from 100 cells and four biological replicates.

© 2023 The Authors

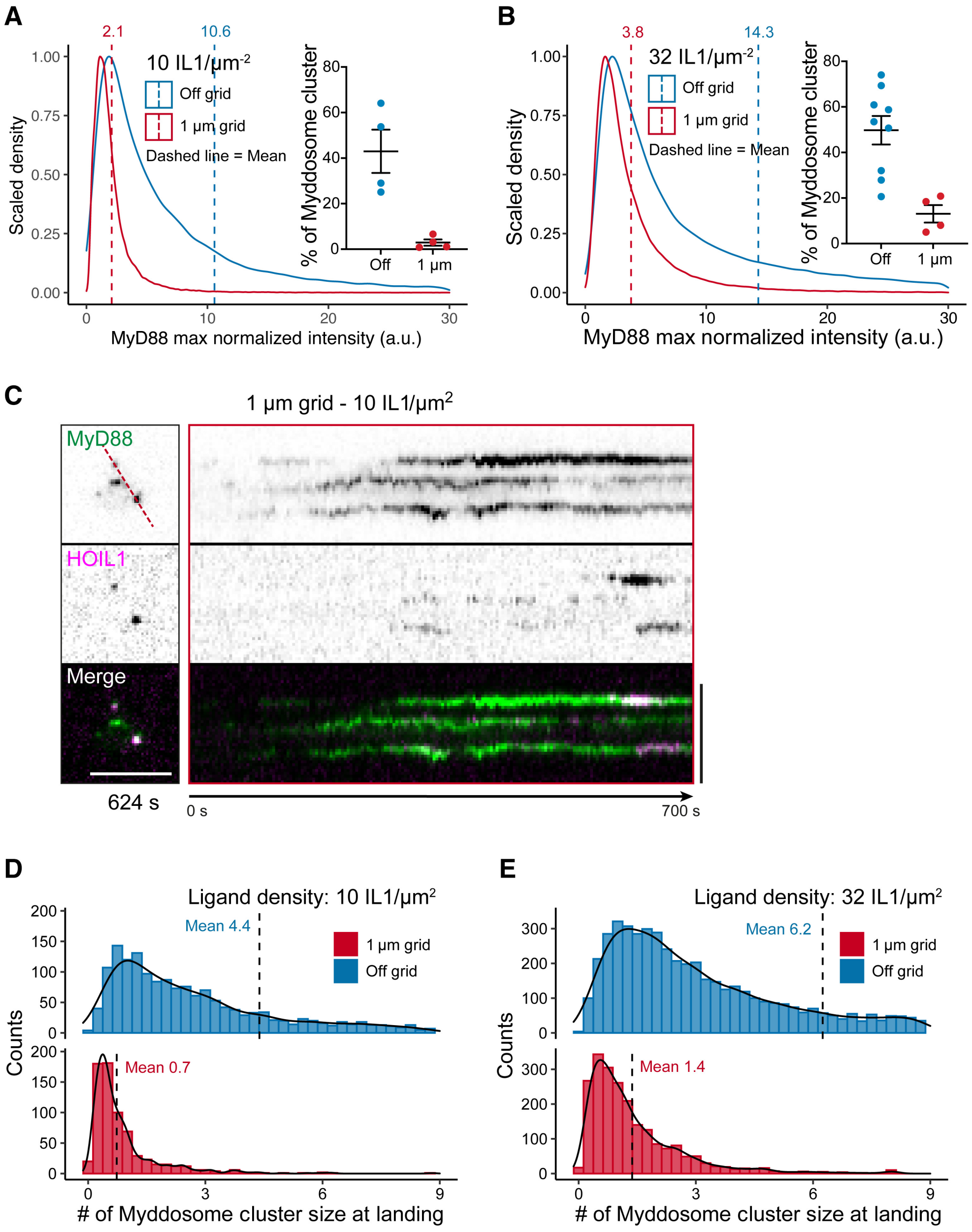

Figure EV5. Characterization of dynamics of MyD88-GFP/mScarlet-HOIL1 cells off and on 1 μm grids.

A, B Scaled density distribution of MyD88 max normalized intensity off grids and on 1 μm grids at a ligand density of 10 (A) or 32 (B) IL1/μm^2. The average MyD88 max normalized intensity (dashed line) at 10 IL1/μm^2 off grids versus on 1 μm grids is 10.6 versus 2.1 and at 32 IL1/μm^2 is 14.3 versus 3.8. Insets are quantifications of the percentage of Myddosome clusters. A Myddosome cluster is defined as a MyD88-GFP puncta containing equal to or greater than 2 Myddosomes. At 10 IL1/μm^2, the percentages of Myddosome clusters off grids versus on 1 μm grids are 43.0 ± 9.5% versus 2.9 ± 1.3%, and at 32 IL1/μm^2 are 49.8 ± 6.3% versus 13.1 ± 3.9%. Bars represent mean ± SEM. At 10 IL1/μm^2, data are measured from 53,852 MyD88 puncta off grids from 74 cells and four biological replicates and 55,075 MyD88 puncta on 1 μm grids from 154 cells and four biological replicates. At 32 IL1/μm^2, data are measured from 118,354 MyD88 puncta off grids from 230 cells and nine biological replicates and 68,819 MyD88 puncta on 1 μm grids from 138 cells and four biological replicates.

C An example of TIRF images of HOIL1 recruitment in EL4 cells expressing MyD88-GFP and mScarlet-HOIL1 stimulated on IL1 functionalized SLBs on 1 μm grids at a ligand density of 10 IL1/μm^2. Kymographs derived from dashed lines overlaid TIRF images (left panel). Scale bar, 5 μm.

D, E Histogram of the average landing size for HOIL1 at 10 IL1/μm^2 (H) and 32 IL1/μm^2 (I) off and on 1 μm grids, overlaid with density plots of the distribution. The landing size of Myddosome is calculated with landing size of MyD88 puncta divided by the intensity of 4.5× GFP. The average landing size of Myddosome at 10 IL1/μm^2 off grids versus on 1 μm grids is 4.4 ± 1.2 versus 0.7 ± 0.2 Myddosomes (Mean ± SEM), measured from 1762 versus 691 MyD88-GFP puncta from 66 versus 86 cells and 4 versus 4 replicates. The average landing size of Myddosome at 32 IL1/μm^2 off grids versus on 1 μm grids is 6.2 ± 1.1 versus 1.4 ± 0.2 Myddosomes (Mean ± SEM), measured from 5,562 versus 2,189 MyD88-GFP puncta from 212 versus 124 cells and 9 versus 4 biological replicates.

 © 2023 The Authors
