# Supplementary material for: Myddosome clustering in IL‐1 receptor signaling regulates the formation of an NF‐kB activating signalosome
Source: EMBO Rep. 2023 Aug 21;24(10):e57233. doi: 10.15252/embr.202357233 (PMC10561168; doi:10.15252/embr.202357233)
Supplement: Supplementary file 3 — Table EV1 [file EMBR-24-e57233-s002.docx]

**Table EV1. Sequences of the *HDR templates***

HDR arms, mScarlet-i ORF, BlastR-2A ORF, START Codon.

| **BlastR-2A-mScarlet-i-TRAF6 HDR template** |
| --- |
| CACCAGACTGGGCATTTAGAAATCCACATGGCATTTTAGATACAGAAAAAGAAAGTTGCCAGTGGTTCCAGCTAGTTGCCCCAGGTCACATTCCCACAACTGACTGTGGCCCTTTGGCTCCCTGCTGTCTGTACCGGTCCCTTCAGTTCAGAGCCCCTAAAGGACCTTCAGTGACATAAAGAACTGGCTCAAGCTGGAGAGGTAGCTCAGCAGCTAAAGGCTCAGCTCGCAGCCACATCTCAGTAGATGCCTAGTAAACATTGACTGTTGTGTGGAAAATCTTGGGCTAAGGTCTGGGCAGGACAGCAGCCTCAGTCCTGTAAACAGATGACAGAGGAAGGTATTGTATTGGAAGTGCTTCACTGTTCAACCTTCTGTTGCTGCAATAGACCACAAACTTTCCTTGAAGGATCTACGTAGTCACCTTTGTAGTCTGCATAGGTGATTCATAAACATCTTGAAGGAGGGAAAGAGATAATTTACCATGAAGTTGTAGTTATGCAACACAGCTCAAAACACTTAAAGCTATTTTGTAAATAACATTGAAACATTATGGTGAACTATGAGGTGGCTATATTAAACGATCTGATCTATTTGCATGCTAAAGCGTTTTGTCGTTGTTTTTCTTGTTGCTGTGTcTGAAGATCGACTGACAACAGAGCTACTATGgccaagcctttgtctcaagaagaatccaccctcattgaaagagcaacggctacaatcaacagcatccccatctctgaagactacagcgtcgccagcgcagctctctctagcgacggccgcatcttcactggtgtcaatgtatatcattttactgggggaccttgtgcagaactcgtggtgctgggcactgctgctgctgcggcagctggcaacctgacttgtatcgtcgcgatcggaaatgagaacaggggcatcttgagcccctgcggacggtgccgacaggtgcttctcgatctgcatcctgggatcaaagccatagtgaaggacagtgatggacagccgacggcagttgggattcgtgaattgctgccctctggttatgtgtgggagggcGGCGGCGGTGAAGGACGAGGCTCACTTCTGACATGCGGTGACGTGGAGGAGAATCCCGGGCCAGTGAGCAAGGGCGAGGCAGTGATCAAGGAGTTCATGCGGTTCAAGGTGCACATGGAGGGCTCCATGAACGGCCACGAGTTCGAGATCGAGGGCGAGGGCGAGGGCCGCCCCTACGAGGGCACCCAGACCGCCAAGCTGAAGGTGACCAAGGGTGGCCCCCTGCCCTTCTCCTGGGACATCCTGTCCCCTCAGTTCATGTACGGCTCCAGGGCCTTCATCAAGCACCCCGCCGACATCCCCGACTACTATAAGCAGTCCTTCCCCGAGGGCTTCAAGTGGGAGCGCGTGATGAACTTCGAGGACGGCGGCGCCGTGACCGTGACCCAGGACACCTCCCTGGAGGACGGCACCCTGATCTACAAGGTGAAGCTCCGCGGCACCAACTTCCCTCCTGACGGCCCCGTAATGCAGAAGAAGACAATGGGCTGGGAAGCGTCCACCGAGCGGTTGTACCCCGAGGACGGCGTGCTGAAGGGCGACATTAAGATGGCCCTGCGCCTGAAGGACGGCGGCCGCTACCTGGCGGACTTCAAGACCACCTACAAGGCCAAGAAGCCCGTGCAGATGCCCGGCGCCTACAACGTCGACCGCAAGTTGGACATCACCTCCCACAACGAGGACTACACCGTGGTGGAACAGTACGAACGCTCCGAGGGCCGCCACTCCACCGGCGGCATGGACGAGCTGTACAAGGGTGGCTCAGGAGGCAGTGGAGGAAGCAGTCTCCTTAACTGTGAGAACAGtTGCGGaTCCAGCCAGTCGTCCAGTGACTGCTGCGCTGCCATGGCCGCCTCCTGCAGCGCTGCAGTGAAAGATGACAGCGTGAGTGGCTCTGCCAGCACCGGGAACCTCTCCAGCTCCTTCATGGAGGAGATCCAGGGCTACGATGTGGAGTTTGACCCACCTCTGGAGAGCAAGTATGAGTGTCCCATCTGCTTGATGGCTTTACGGGAAGCAGTGCAAACACCATGTGGCCACAGGTTCTGCAAAGCCTGCATCATCAAATCCATAAGGTATTTGATTTTGAGAAAGCATGGTGCCCTACAGCAACCAAATGTAAAGAGCATGTTAGAAACAGGCCATCCCAAGAATAGGCAGACAGAAAACACTGGTCAGGAACACAGCAGAATGGACAGAAACCTGAGACAGTTGGGGTCCCATCCCAGTTTGTACTACGGTATGAACAGAGGCTTGCTTCCGTGTCTTCCCACCCAGCCTCCAGGAAAACTGCAAAGTCCATAGCGACTTTTGCCTCAAGCAGTTCTATTGCAGACTAGGAGAGGATTCTGTGTTTCATGAAAGTAGTATTTCTAATTTGCCTTAAACTCAGCTACTTGTG |
| **BlastR-2A-mScarlet-i-HOIL1 HDR template** |
| CAGAGCTAGGCGCTGCCTGGAGTCGGCCGGGTGGGGAGAGGGGGGGATCATGGGTCCCTAGACCAAGTGACTCTGGTCCGCCCAACCAAGTGTGGCGCGAAAGTGCCAGGAGCAGTCTAGAGGTCTGCCCCAGTTGGTGACCCTGCAGAGTCTCGGGTGAGGGGACAGTTTTCCGTTCGGTCTGCAGCACTTCCGGGACTGCCACTTTCACTTTCTCTTCCGGGGACGCTGCTCGCGCTGTCTTCCGGGTAGCGCGAGCCTCCGGGCTGGGGTTCCGGACGCTAGGGCGCCCGCGCCGGCTGGCTGGCCGGCCTCCCTCCGTCCCTCGCTTTTGGGTCGTGGTTACTCATCGCCCTTTGCAGACTTGGCTCGGGGCCTCCTTCGCTGTCCGTGTCCCTCGCGGGGCCCCATTGGTTCCCAGTGCCCGGCCTCGGGCCCTGGGCAGTGTGATGCTGCCCGAGTGCGGACTGGAACACACGCACGAGGCCGGCTAGGGCAGAGTTGCTTCTACCTTCCCGCTCTCTCCCAGGTTACCTCAAAGTAGCGTTTTCCGGAAGCAGCAGCCCTTTCTGAGGGGATGGGCGCAGCCAGGCCAGATGgccaagcctttgtctcaagaagaatccaccctcattgaaagagcaacggctacaatcaacagcatccccatctctgaagactacagcgtcgccagcgcagctctctctagcgacggccgcatcttcactggtgtcaatgtatatcattttactgggggaccttgtgcagaactcgtggtgctgggcactgctgctgctgcggcagctggcaacctgacttgtatcgtcgcgatcggaaatgagaacaggggcatcttgagcccctgcggacggtgccgacaggtgcttctcgatctgcatcctgggatcaaagccatagtgaaggacagtgatggacagccgacggcagttgggattcgtgaattgctgccctctggttatgtgtgggagggcGGCGGCGGTGAAGGACGAGGCTCACTTCTGACATGCGGTGACGTGGAGGAGAATCCCGGGCCAGTGAGCAAGGGCGAGGCAGTGATCAAGGAGTTCATGCGGTTCAAGGTGCACATGGAGGGCTCCATGAACGGCCACGAGTTCGAGATCGAGGGCGAGGGCGAGGGCCGCCCCTACGAGGGCACCCAGACCGCCAAGCTGAAGGTGACCAAGGGTGGCCCCCTGCCCTTCTCCTGGGACATCCTGTCCCCTCAGTTCATGTACGGCTCCAGGGCCTTCATCAAGCACCCCGCCGACATCCCCGACTACTATAAGCAGTCCTTCCCCGAGGGCTTCAAGTGGGAGCGCGTGATGAACTTCGAGGACGGCGGCGCCGTGACCGTGACCCAGGACACCTCCCTGGAGGACGGCACCCTGATCTACAAGGTGAAGCTCCGCGGCACCAACTTCCCTCCTGACGGCCCCGTAATGCAGAAGAAGACAATGGGCTGGGAAGCGTCCACCGAGCGGTTGTACCCCGAGGACGGCGTGCTGAAGGGCGACATTAAGATGGCCCTGCGCCTGAAGGACGGCGGCCGCTACCTGGCGGACTTCAAGACCACCTACAAGGCCAAGAAGCCCGTGCAGATGCCCGGCGCCTACAACGTCGACCGCAAGTTGGACATCACCTCCCACAACGAGGACTACACCGTGGTGGAACAGTACGAACGCTCCGAGGGCCGCCACTCCACCGGCGGCATGGACGAGCTGTACAAGGGTGGCTCAGGAGGCAGTGGAGGAAGCGACGAGAAAACCAAGAAAGGTGGGCACCGATTGCAGCTGGTGGAAGTGGACCCTAGGCCTAGTCAGTGCAAAAATAGGGGTCGGAGACTATGTGAACGGAGTTGGAGAGCTCCTGGTTGTTAGGAAAACAAACTTAAGTTGTCCAGAGTAGGACCGAGAGGGGAACATCCATCCCTCCCCCTGGCCCTTTCACTCAGAACTGGGGGAACTCCGATAATGCCACTGTTCTGTTTGCTGGAAACCTTGTCTGAATCTCCCCCCCCCCTTTTTTTTTACTACTGGGGATTGAACCCGGGTTTTCATGCATGCTAGGCAAATGCTCTATTTCTGAGCTATGCCTCTAGTACATGGCTTTTCTGTTTTGTCCCAACCACTTCTTGCCCAAGGCCTTTGCCCACTCTCTGGCCTCTTGGAACCGTCTTCCTTATTGGATATTGGAATGCCCAGCAACCGCTCCTTCAGGCCTCAGTTTCCCTTACTTCCTAGAGGTCTCCCCTGACCCCTTACTCAGTTATGGCTAGAATGGACCACAGCCTGATTTTCTCATCAAGTTTTAATTTTCTATTTTCTTGTTGAACTTAAAGAGTTTTATAAGGAGAAGAATGCTCTTCGACTTAACCGCTGTTAAAAGAGTCTACTTTCTGGAATAGTGGGTGGCATACAGGAGAGCTCAAGAAATGCTTGTCAGATAAATGAATGGAAGTATGAGGACTCTGTGAGCATGGAGGAAAGAGGGTACAGGGGAGGGAGGACTCCGTGAGTACAGGGTACAGAGGAGTGAGGACCGGAAAA |
