# Supplementary material for: Myddosome clustering in IL‐1 receptor signaling regulates the formation of an NF‐kB activating signalosome
Source: EMBO Rep. 2023 Aug 21;24(10):e57233. doi: 10.15252/embr.202357233 (PMC10561168; doi:10.15252/embr.202357233)
Supplement: Supplementary file 12 — PDF+ [file EMBR-24-e57233-s019.pdf]

Article

SOURCE DATA TRANSPARENT PROCESS OPEN ACCESS

EMBO reports

Myddosome clustering in IL-1 receptor signaling regulates the formation of an NF-kB activating signalosome

Fakun Cao, Rafael Deliz-Aguirre, Fenja HU Gerpott, Elke Ziska & Marcus J Taylor*

Abstract

IL-1 receptor (IL-1R) signaling can activate thresholded invariant outputs and proportional outputs that scale with the amount of stimulation. Both responses require the Myddosome, a multiprotein complex. The Myddosome is required for polyubiquitin chain formation and NF-kB signaling. However, how these signals are spatially and temporally regulated to drive switch-like and proportional outcomes is not understood. During IL-1R signaling, Myddosomes dynamically reorganize into multi-Myddosome clusters at the cell membrane. Blockade of clustering using nanoscale extracellular barriers reduces NF-kB activation. Myddosomes function as scaffolds that assemble an NF-kB signalosome consisting of E3-ubiquitin ligases TRAF6 and LUBAC, K63/M1-linked polyubiquitin chains, phospho-IKK, and phospho-p65. This signalosome preferentially assembles at regions of high Myddosome density, which enhances the recruitment of TRAF6 and LUBAC. Extracellular barriers that restrict Myddosome clustering perturbed the recruitment of both ligases. We find that LUBAC was especially sensitive to clustering with 10-fold lower recruitment to single Myddosomes than clustered Myddosomes. These data reveal that the clustering behavior of Myddosomes provides a basis for digital and analog IL-1R signaling.

Keywords cell signaling; IL-1; Myddosome; polyubiquitin signaling; signalosome

Subject Categories Membrane & Trafficking; Post-translational Modifications & Proteolysis; Signal Transduction

DOI 10.15252/embr.202357233 | Received 24 March 2023 | Revised 20 July 2023 | Accepted 27 July 2023 | Published online 21 August 2023

EMBO Reports (2023) 24: e57233

Introduction

Signaling pathways can give digital or "switch-like" responses that are invariant (Shah & Sarkar, 2011) or alternatively give analog responses that are proportional to the amount of stimulatory input (Nunns & Goentoro, 2018). For instance, in the innate immune system, IL-1 activation of NF-kB has both an invariant component and a response proportional to the stimulating dose (DeFelice *et al*, 2019; Son *et al*, 2021). Critical to these responses is the temporal and spatial control of reactants within a signaling pathway. Protein effectors must be brought together at a precise point within the cell to ensure accurate signal transduction. One way in which signaling pathways seem to achieve this is through the formation of signalosomes: subcellular compartments containing clusters of receptors and signaling effectors. Signalosomes are found in multiple receptor signaling systems such as tyrosine kinases, immune receptors, and Wnt receptors (Case *et al*, 2019). In the case of IL-1 signaling, an NF-kB signalosome assembles in response to stimulation (Tarantino *et al*, 2014). Thus, a key question is how signalosomes, such as that associated with NF-kB activation, can activate both invariant and proportional responses.

A critical component of many signalosomes is protein scaffolds that can bind and concentrate multiple signaling effectors (Wu, 2013; Jaqaman & Ditlev, 2021). The ability of protein scaffolds to oligomerize or self-assemble plays a crucial role in signalosome formation and downstream signaling (Ditlev *et al*, 2018). In the immune system, oligomeric protein scaffolds serve a central role in tuning the intensity and duration of signaling responses (Wu, 2013). The Myddosome is an oligomeric complex, composed of MyD88, IRAK4, and IRAK1 (Lin *et al*, 2010a), that is crucial for IL-1R signal transduction and an inflammatory innate immune response. The Myddosome activates the generation of K63-ubiquitin linked (K63-Ub) chains via directly interacting with the E3 ligase TNF Receptor-Associated Factor 6 (TRAF6; Ye *et al*, 2002). Myddosomes can also activate the generation of M1-Ubiquitin linked (M1-Ub) chains via the linear ubiquitin chain assembly complex (LUBAC; Tokunaga *et al*, 2009). K63-Ub and M1-Ub chains recruit the IκB kinase (IKK) complex that activates NF-kB signaling and results in the translocation of the RelA NF-kB subunit to the nucleus (Wertz & Dixit, 2010; Iwai, 2012). This signaling pathway can encode both digital and analog outputs as defined by downstream readouts such as RelA dynamics or transcriptional responses (Tay *et al*, 2010; Hughey *et al*, 2015; Cheng *et al*, 2021). However, whether and how the Myddosome encodes both invariant and proportional outputs upstream of NF-kB has not been investigated.

Here, we address this problem using live-cell imaging to visualize Myddosome formation and downstream signal transduction in

Max Planck Institute for Infection Biology, Berlin, Germany
*Corresponding author. Tel: +49 3028460138; E-mail: taylor@mpiib-berlin.mpg.de

© 2023 The Authors. Published under the terms of the CC BY 4.0 license

response to IL-1 stimulation. We observe that Myddosomes reorganize into clusters or regions of the plasma membrane that contain a high density of complexes. Physically limiting Myddosome clustering with extracellular barriers diminishes NF-kB activation. We find that Myddosomes function as scaffolds that nucleate a signalosome containing K63/M1-Ub chains and markers of NF-kB activation. Single Myddosomes can nucleate the formation of this signalosome, suggesting it is an invariant or digital signaling output of the complex. However, this NF-kB signalosome preferentially formed at clusters and the degree of Myddosome clustering proportionally increases the size of this signalosome. In particular, clustering amplifies the production of M1-Ub. Live-cell imaging revealed that the ubiquitin ligases TRAF6 and LUBAC are preferentially recruited to Myddosome clusters. Restricting clustering diminished TRAF6 recruitment and severely perturbed HOIL1 recruitment. We conclude that clustering is an important determinant of E3 ubiquitin ligase recruitment, and this dynamic encodes a signaling output that is proportional to the nanoscale density of complexes within the cluster. These results suggest a mechanism for how Myddosomes can encode both digital and analog responses upstream of NF-kB in IL-1R signaling.

Results

Myddosomes dynamically reorganize into clusters

Understanding how the IL-1R and Myddosomes encode digital and analog outputs requires understanding where these differences arise within the signaling network. Therefore, to uncover the link between the spatial organization of Myddosomes and the production of downstream signaling outputs, we used a supported lipid bilayer (SLB) system functionalized with IL-1 (Deliz-Aguirre *et al*, 2021) to visualize the dynamics of IL-1R-Myddosome signal transduction. We pipetted EL4 cells expressing MyD88-GFP into chambers containing SLBs and imaged them as they land on this surface and bind to IL-1. We found that MyD88-GFP assembles into puncta at the cell surface (Fig 1A). Initially, MyD88-GFP puncta are spatially segregated, but over time MyD88-GFP puncta move and coalesce, forming brighter puncta and eventually larger dense patch-like structures at the cell–SLB interface (Fig 1A; Movie EV1). While many Myddosome clusters were highly stable, in some cases, we observe that Myddosome clustering was not unidirectional, with discrete Myddosomes undergoing merging and subsequent splitting (Fig EV1A). This unstable nature of a subset of clusters might be because they are composed of discrete Myddosomes confined to separate membrane protrusions and contact zones with the SLB (Fig EV1B), thus allowing them to split and move apart. Alternatively, more stable Myddosome clusters might be confined to a continuous contact zone with the SLB (Fig 1B). These clusters are similar to large Myddosome structures observed in Toll-like receptor 4 signaling that are associated with stronger NF-kB responses (Latty *et al*, 2018). We decided to investigate how these dynamic Myddosome clusters are involved in IL-1R signal transduction.

The dynamic clustering of MyD88 puncta within the plane of the cell membrane suggests that Myddosomes are tethered to the inner leaflet of the plasma membrane. This tethering is likely due to heterotypic TIR domain interactions between MyD88 and the IL-1R/IL-1RAcP complex (Nimma *et al*, 2017). We predict, if this model is correct, extracellular barriers that restrict the diffusion of SLB-tethered IL-1 would restrict Myddosome mobility and clustering (Fig 1B). To test this model, we used coverslips nano-printed with chromium barriers arranged into multiple 0.5 mm square grids. Within these square grids, chromium grid lines were printed into 1

Figure 1. Myddosomes are tethered to the cell surface and extracellular barriers inhibit Myddosome coalescence and diminish NF-kB activation.

A Timelapse TIRF microscopy images showing an EL4-MyD88-GFP cell interacting with a IL-1 functionalized SLB. MyD88-GFP assembles into puncta that cluster and coalesce at the cell:SLB interface. Scale bar, 2 μm.

B Schematic illustrating a working model for Myddosomes being tethered to the plasma membrane via interaction with IL-1R bound to IL-1. Based on this model, we predict physical barriers (on grid) would restrict the diffusion of IL-1 on the SLB and limit Myddosome clustering. With no external barriers present (off grid), MyD88 puncta can merge to form larger multicomplex assemblies.

C TIRF and bright-field microscopy images of EL4-MyD88-GFP cells incubated for 30 min with IL-1 functionalized SLBs formed off grid and on 1 and 2.5 μm grids. In the presence of a 1 and 2.5 μm grids, Myddosomes only coalesce within individual corrals and do not form multicomplex clusters. Scale bar, 5 μm.

D Time series showing Myddosome formation in an EL4 cell interacting with both continuous and 2.5 μm gridded partitioned SLBs. *t* = 0 s denotes the start of cellular observation. Scale bar, 5 μm.

E Kymographs from panel (D) showing the coalescence of MyD88-GFP puncta off grid and the restricted movement of MyD88-GFP puncta on 2.5 μm grids. Scale bar, 1 μm.

F Quantification of MyD88-GFP puncta maximum fluorescence intensity normalized to GFP from cells stimulated off and on 2.5 or 1 μm grids, at a ligand density of 10 IL-1/μm^2. Violin plots show the distribution of average max puncta intensities from individual cells across replicates. Data points superimposed on the violin plots are the averages from independent experimental replicates. The average max MyD88 puncta intensity (mean ± SEM): for off grid is 7.5 ± 1.8 GFPs, *n* = 8 biological replicates, with 88,304 MyD88-GFP puncta from 161 cells measured in total across all replicates; for 2.5 μm grids is 5.2 ± 1.7 GFPs, *n* = 3 biological replicates, with 13,164 MyD88-GFP puncta from 31 cells measured in total across all replicates; for 1 μm grids is 2.3 ± 0.1 GFPs, *n* = 8 biological replicates, with 126,600 MyD88-GFP puncta from 254 cells measured in total across all replicates. Bars represent mean ± SEM.

G Widefield images showing RelA localization in unstimulated EL4 cells and EL4 cells stimulated by SLB formed on and off grids. EL4 was fixed 30 min after addition to IL-1-functionalized SLBs and stained for RelA (magenta); DAPI-stained nuclei (blue). Scale bar, 10 μm.

H Quantification of RelA nucleus to cytoplasm ratio. Violin plots show the distribution of measurements from individual cells. Data points superimposed on the violin plots are the averages from independent experiments. The RelA nucleus-to-cytoplasm ratio of single cells marked with X in panel (G) is superimposed on the violin plot. RelA nucleus-to-cytoplasm ratio off grids, on 2.5 and 1 μm grids, and unstimulated conditions are 0.45 ± 0.01, 0.34 ± 0.02, 0.32 ± 0.02, 0.21 ± 0.03 (mean ± SEM), respectively. The *P*-value are * = 0.0133 and ** = 0.0027. Bars represent mean ± SEM (*n* = 3–5 biological replicates, with a total of 18,370, 3,627, 2,988, and 965 cells measured off grids, on 2.5 and 1 μm grids, and unstimulated conditions, respectively). Statistical significance is determined using unpaired two-tailed Student's *t*-test.

Source data are available online for this figure.

 © 2023 The Authors

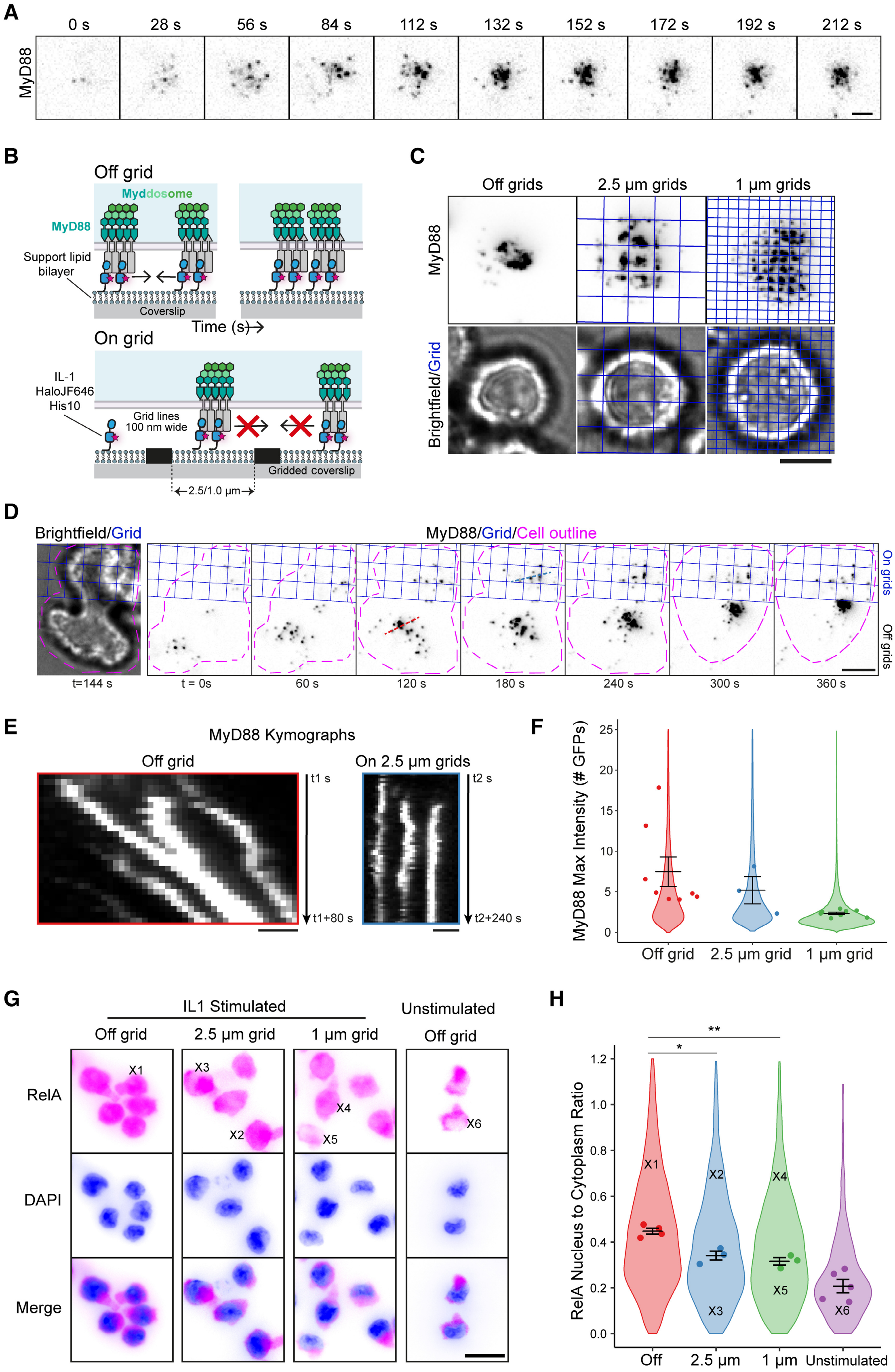
A
0 s
28 s
56 s
84 s
112 s
132 s
152 s
172 s
192 s
212 s
MyD88
B
Off grid
Myddosome
MyD88
Support lipid bilayer
Coverslip
Time (s)
On grid
IL-1 HaloJF646 His10
Grid lines 100 nm wide
Gridded coverslip
2.5/1.0 μm
C
Off grids
2.5 μm grids
1 μm grids
MyD88
Brightfield/Grid
D
Brightfield/Grid
MyD88/Grid/Cell outline
On grids
Off grids
t=144 s
t = 0s
60 s
120 s
180 s
240 s
300 s
360 s
E
MyD88 Kymographs
Off grid
On 2.5 μm grids
t1 s
t1+80 s
t2 s
t2+240 s
F
MyD88 Max Intensity (# GFPs)
Off grid
2.5 μm grid
1 μm grid
G
IL1 Stimulated
Unstimulated
Off grid
2.5 μm grid
1 μm grid
Off grid
RelA
DAPI
Merge
H
RelA Nucleus to Cytoplasm Ratio
Off
2.5 μm
1 μm
Unstimulated

Figure 1.

or 2.5 μm square corrals (Fig EV1C). The chromium grid lines function as physical barriers and create an array of corralled SLBs with uniform dimensions. We confirmed SLB formation within these grids and that IL-1 ligands are freely mobile within corrals, but diffusion between corrals is restricted (Fig EV1D and E). In cells that landed on nanopatterned grids, MyD88 puncta were confined to individual corrals (Fig 1C). We imaged cells that straddled the boundary between the 2.5 μm grid and continuous coverslip, so we could analyze Myddosome dynamics on/off grids within the same cell (Fig 1D; Movie EV2). Kymograph analysis reveals that in the same cell only off-grid MyD88 puncta clustered. In contrast, MyD88 puncta on the 2.5 μm grid were confined to individual corrals and did not merge with puncta in adjacent corrals (Fig 1E). We conclude that Myddosomes are biochemically coupled to extracellular IL-1 via IL-1R, and extracellular barriers limit the diffusion of complexes within the plasma membrane.

To determine whether clustering regulates downstream signaling requires tools that can isolate a single Myddosome for comparative analysis to clustered Myddosomes. We analyzed the size distribution of MyD88 puncta on/off grids (Fig 1F). Off-grid MyD88 puncta had a broad size distribution and a mean MyD88 copy number of 7.5 ± 1.8 MyD88s (Fig 1F), suggesting a mix of clusters and single complexes. However, on 2.5 or 1 μm grids, the mean MyD88 copy number was 5.2 ± 1.7 or 2.3 ± 0.1 MyD88s (Mean ± SEM, Fig 1F, also see Materials and Methods). Only 9.3 ± 1.6% of MyD88 puncta on 1 μm grids had an intensity consistent with ≥ 1 Myddosome complexes and 1.8 ± 0.6% puncta had an intensity consistent with ≥ 2 Myddosome complexes (see Materials and Methods and Fig 1F). Thus, the majority of MyD88-GFP puncta on 1 μm grids are small transient MyD88 assemblies or single Myddosome complexes (Deliz-Aguirre *et al*, 2021), and nanopatterned IL-1 functionalized SLBs can spatially isolate single Myddosomes. In conclusion, nanopatterned coverslips are an effective tool to assay how the spatial organization of Myddosomes is functionally connected to digital and analog signaling outputs.

Inhibiting Myddosome clustering diminishes RelA translocation to the cell nucleus

We tested whether inhibition of clustering perturbed NF-kB signaling by measuring RelA translocation to the nucleus. In unstimulated cells (incubated with unfunctionalized SLBs), RelA staining is limited to the cytosol and depleted within the cell nucleus (Fig 1G). In cells incubated with IL-1 functionalized SLBs without grids, RelA translocates from the cell cytosol to the nucleus, resulting in stronger nuclear staining (Fig 1G). For cells on grids, a mixture of both events was observed (2.5 and 1 μm grids, Fig 1G). When we quantify RelA translocation, we found that the nucleus-to-cytoplasm ratio of RelA staining significantly decreased on 1 or 2.5 μm grids (normalized RelA nucleus-to-cytoplasm ratio of 0.45 ± 0.01 off grid versus 0.34 ± 0.02 and 0.32 ± 0.02 on 2.5 and 1 μm grids, respectively, mean ± SEM, Fig 1H). We conclude that the inhibition of Myddosome clustering impacts NF-kB activation and RelA translocation to the nucleus. The implication of these results is that Myddosome dynamics and spatial density at the cell surface are linked to the production of signaling outputs required for NF-kB activation.

Myddosomes colocalize with an NF-kB-activating signalosome composed of K63-Ub/M1-Ub polyubiquitin chains, phospho-IKK, and phospho-p65

Innate immune signaling complexes are proposed to function as signaling scaffolds that recruit and activate downstream effectors (Wu, 2013). We speculated that the spatial organization of a signaling complex could regulate its scaffolding function, and this could be the basis for invariant or proportional signaling responses. This scaffolding model suggests spatial colocalization between Myddosomes and biochemical signaling reactions. We examined the colocalization of Myddosomes with IL-1 signaling outputs such as K63-Ub, M1-Ub, phosphorylated IκB kinase (pIKK) complex, and phosphorylated RelA subunit p65 (pp65) using immunofluorescence and TIRF microscopy (Fig 2A; Appendix Fig S1A). We found these signaling outputs had a punctate staining pattern that colocalized with dense patches of clustered MyD88-GFP puncta (Fig 2A). Detailed analysis of these Myddosomes patches shows that MyD88 was organized into heterogenous puncta of different sizes and irregular shapes (Fig 2B). While these puncta of K63-Ub, M1-Ub, pIKK, and pp65 staining did not uniformly coat MyD88 patches, these structures were clearly associated with MyD88 clusters. These results confirm that downstream signaling outputs are generated at cell surface Myddosomes.

We used structured illumination microscopy (SIM) to image the spatial organization of pIKK and MyD88 puncta with higher resolution and within the entire cellular volume (Fig 2C; Appendix Fig S1B). Consistent with our TIRF studies, we found that pIKK punctate structures colocalized with MyD88-GFP puncta at the cell surface (Fig 2C). In some instances, SIM revealed that pIKK puncta partially overlapped or were adjacent to MyD88-GFP puncta (inset, Fig 2C). Z-stack analysis revealed that pIKK puncta localized to the cell-bilayer interface and were rarely found deeper within the

Figure 2. Myddosomes colocalize with a NF-kB signalosome composed of K63-Ub and M1-Ub chains and phospho-IKK and phospho-p65.

A TIRF images of fixed EL4-MyD88-GFP cells off grids and stained with antibodies against K63-Ub, M1-Ub, pIKK, and pp65. Cells were activated on IL-1 functionalized SLBs for 30 min before fixation. Scale bar, 5 μm.

B A magnified view of the large patch-like Myddosome clusters from the highlighted region of interest in panel (A) (yellow box on merge images). Scale bar, 1 μm.

C Structured illumination microscopy images of Myddosome clusters stained with anti-pIKK. Top row right, insets show the detail of Myddosome staining with anti-pIKK. Inset taken from regions of interest overlaid the merge image (yellow boxes 1 and 2). Bottom row, x-z view slice taken from yellow line overlaid on the merge image (top row). Myddosome and pIKK staining localize the cell–SLB interface. Blue dashed line defines the nucleus volume determined from the DAPI stain. Scale bar in main image and Z projection, 1 μm; scale bar inset, 0.5 μm.

D Schematic showing working model for how Myddosome clustering could enhance the generation of K63/M1-Ub, pIKK, and pp65 and a NF-kB signalosome. We hypothesize that the Myddosome clustering creates regions with a high density of complexes, and this will lead to enhanced production of signaling intermediates such as K63-Ub and M1-Ub chains, pIKK and pp65.

Source data are available online for this figure.

 © 2023 The Authors

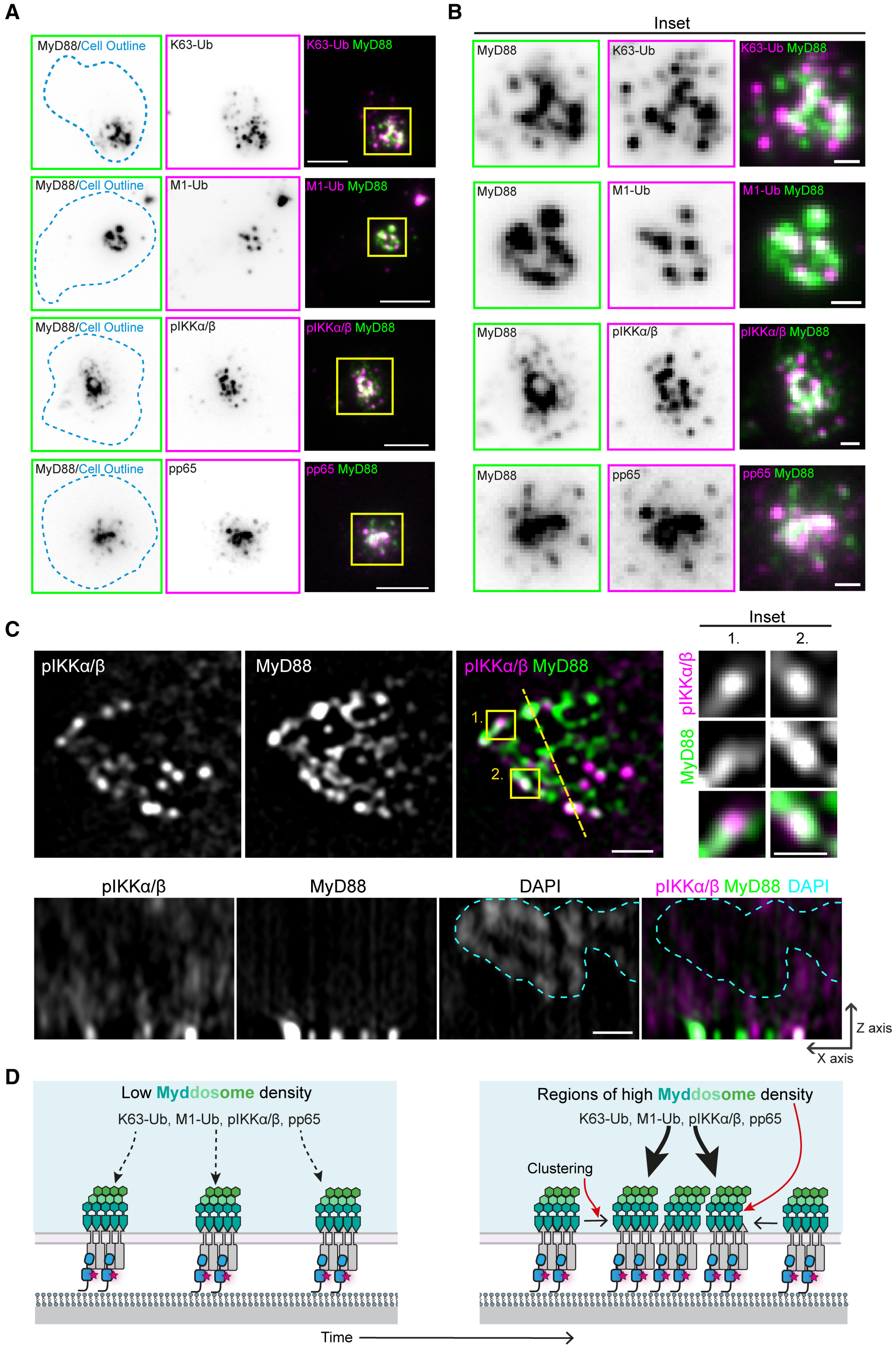
A
MyD88/Cell Outline
K63-Ub
K63-Ub MyD88
M1-Ub
M1-Ub MyD88
pIKKα/β
pIKKα/β MyD88
pp65
pp65 MyD88
B
Inset
MyD88
C
Inset
1.
2.
DAPI
pIKKα/β MyD88 DAPI
Z axis
X axis
D
Low Myddosome density
K63-Ub, M1-Ub, pIKKα/β, pp65
Regions of high Myddosome density
Clustering
Time

Figure 2.

cytosol (x-z view, Fig 2C). In summary, Myddosomes function as a scaffold and a focal point for the formation of an NF-kB signalosome that is composed of K63/M1-Ub chains and phosphorylated IKK complex and NF-kB subunits.

The degree of Myddosome clustering displays a linear relationship with NF-kB signalosome size

Diminished RelA nuclear translocation on nanopatterned grids (Fig 1H) suggested a connection between the spatial organization of Myddosomes and the degree of NF-kB activation. We wondered whether this connection is because multi-Myddosome clusters, as compared to single Myddosomes, function as high-efficiency scaffolds and focal points for enhanced NF-kB signalosome assembly (Fig 2D). To test this hypothesis, we used TIRF microscopy to analyze the spatial relationship between Myddosome density and accumulation of pp65 and pIKK. A scatter plot of MyD88 puncta intensity versus pp65 or pIKK staining intensity (Fig 3A and B) shows a linear relationship (R = 0.64 and 0.71 from pp65 and pIKK, Fig 3A and B; Appendix Fig S1C and D). Thus, brighter MyD88 puncta, which most likely corresponded to clusters of multiple Myddosome complexes (Fig 2D), had increased levels of pIKK and pp65 (see insets, Fig 3A and B for example). Therefore, the signaling output of Myddosomes increases proportionally with the degree of clustering: the higher the density of complexes within a Myddosome cluster, the greater the intensity of pIKK and pp65.

We used SLBs formed on 1 and 2.5 μm grids to inhibit the formation of Myddosome clusters (Fig 1F) and assayed how this impacted pp65 and pIKK staining. We found that cells on grids still assembled MyD88 puncta that colocalized with pp65 and pIKK (Fig 3C and D; Appendix Fig S1C and D). Scatter plot analysis of MyD88 puncta assembled revealed that, similar to off-grid (Fig 3A and B), there was a linear relationship between puncta intensity and associated pp65/pIKK staining (Fig 3E and F). Similar to above (Fig 3A and B), these data suggest a linear relationship between the density of Myddosome complexes and pIKK and pp65 production. However, restricting the degree of clustering proportionally reduced pp65 and pIKK production.

We compared the mean pp65/pIKK intensity of puncta classified as single or clustered Myddosomes with puncta formed on 2.5 and 1 μm grids (Materials and Methods and Appendix Fig S1E and F). We found that Myddosome clusters had a 5- and 10-fold greater mean pp65 and pIKK staining intensity compared with MyD88 puncta on 1 and 2.5 μm grids that were most likely single complexes (Fig 3G and H). Interestingly, puncta classified as single Myddosomes off grid had statistically greater mean pp65/pIKK intensity than single Myddosomes formed on grids. We noticed that off grid some of these single Myddosomes with high pp65/pIKK staining intensity were closely associated with Myddosome clusters; this suggested Myddosome clusters could enhance signaling output for adjacent complexes. Alternatively, these off-grid single Myddosomes with high staining intensity could be linked or connected to Myddosome complexes deeper in the cell, which are not illuminated by the TIRF field, and this could also explain the greater staining intensity. The greater concentrations of pp65 and pIKK at Myddosome clusters suggest they are hot spots for NF-kB signaling, and that the localized production of these outputs is proportional to the degree of complex density within clusters.

We examined the relationship between MyD88-GFP puncta intensity and staining with antibodies against K63-Ub and M1-Ub chains (Fig 4A–D; Appendix Fig S1G–J). As above, we found a linear correlation between MyD88 puncta intensity and K63/M1-Ub staining intensity (R = 0.75 and 0.73 for K63-Ub and M1-Ub staining intensity, scatter plot, Fig 4A and B). MyD88 puncta that formed on 1 and 2.5 μm grids still colocalized with punctate K63/M1-Ub structures but overall had lower staining intensities (Fig 4C and D). However, similar to off-grid, there was a correlation between MyD88 puncta and K63/M1-Ub intensity on grids (Fig 4C–F). We found that Myddosome clusters had a 4-fold greater mean K63-Ub intensity and a 3-fold greater mean M1-Ub intensity than single Myddosomes and puncta on 1 and 2.5 μm grids (Fig 4G and H, Appendix Fig S1I and J). Similar to pp65 and pIKK staining, single Myddosomes off

Figure 3. Comparison of pIKK and pp65 antibody staining at Myddosomes assembled off and on nanopatterned grids.

A, B Top, TIRF images of fixed EL4-MyD88-GFP cells incubated with IL-1 functionalized SLBs for 30 min and stained with antibodies against pp65 (A) or pIKK (B). Scale bar, 5 μm. Region of interest (red box, merge image) shows an example of MyD88-GFP puncta that colocalizes with pp65 (A) or pIKK (B) puncta. Bottom, 2D histograms of the distribution of MyD88 puncta intensity and associated pp65 (A) or pIKK (B) staining intensity. Linear fit is shown as a blue line superimposed on 2D histograms (Pearson correlation coefficient, R, of linear fit labeled on 2D histograms). Blue-shaded regions on scatter plot high MyD88 puncta classified as clustered Myddosomes. Bottom right, zoomed images of the region of interest (red box overlaid merge image, top) show MyD88-GFP channel and associated pp65 (A) and pIKK (B) channel (pp65/pIKK images are displayed with Fire LUT). Red data points on the 2D histogram are from indicated puncta in the MyD88-GFP image (numbered red arrows). Scale bar, 1 μm.

C, D TIRF images of fixed EL4-MyD88-GFP cells incubated with partitioned IL-1 functionalized SLBs (2.5 μm top row and 1 μm bottom row) and stained with anti-pp65 (C) or anti-pIKK (D). Region of interest (red box overlaid merge image) shows examples of MyD88-GFP puncta that colocalize with pp65 (A) or pIKK (B) puncta. Scale bar, 5 μm. Far right, zoomed image of pp65 (C) or pIKK (D) puncta (from region of interest overlaid merge image) displayed with Fire LUT. Scale bar, 1 μm.

E, F 2D histogram of MyD88-GFP puncta intensity and associated pp65 (E) or pIKK (F) staining intensity on 2.5 and 1 μm grids. Linear fit is shown as a blue line superimposed on 2D histograms (Pearson correlation coefficient, R, of linear fit labeled on 2D histograms).

G, H Quantification of mean pp65 (G) or pIKK (H) staining intensity for puncta classifieds as single or clustered Myddosomes, and MyD88 puncta formed on 2.5 and 1 μm grids. The normalized mean intensity for clusters, single Myddosomes MyD88 puncta on 2.5 and 1 μm grids are the following: for pp65 0.318 ± 0.044, 0.163 ± 0.009, 0.059 ± 0.005 and 0.057 ± 0.008; for pIKK 0.393 ± 0.051, 0.130 ± 0.004, 0.037 ± 0.006 and 0.035 ± 0.007 (a.u., mean ± SEM, mean value states in the order they appear on plot, left to right). Violin plots show the distribution of all segmented MyD88 puncta. Data points superimposed on the violin plots are the averages from independent experiments. P-values are * < 0.05, *** < 0.001, **** < 0.0001. Bars represent mean ± SEM (n = 3–4 biological replicates for pp65, with 10,273, 14,009 and 2,675 puncta off grid, on 2.5 μm and 1 μm grid measured in total across all replicates; n = 4–5 biological replicates for pIKK, with 2,375, 35,496 and 59,593 puncta off grid, on 2.5 μm and 1 μm grid measured in total across all replicates). Statistical significance is determined using unpaired two-tailed Student's t-test.

Source data are available online for this figure.

 © 2023 The Authors

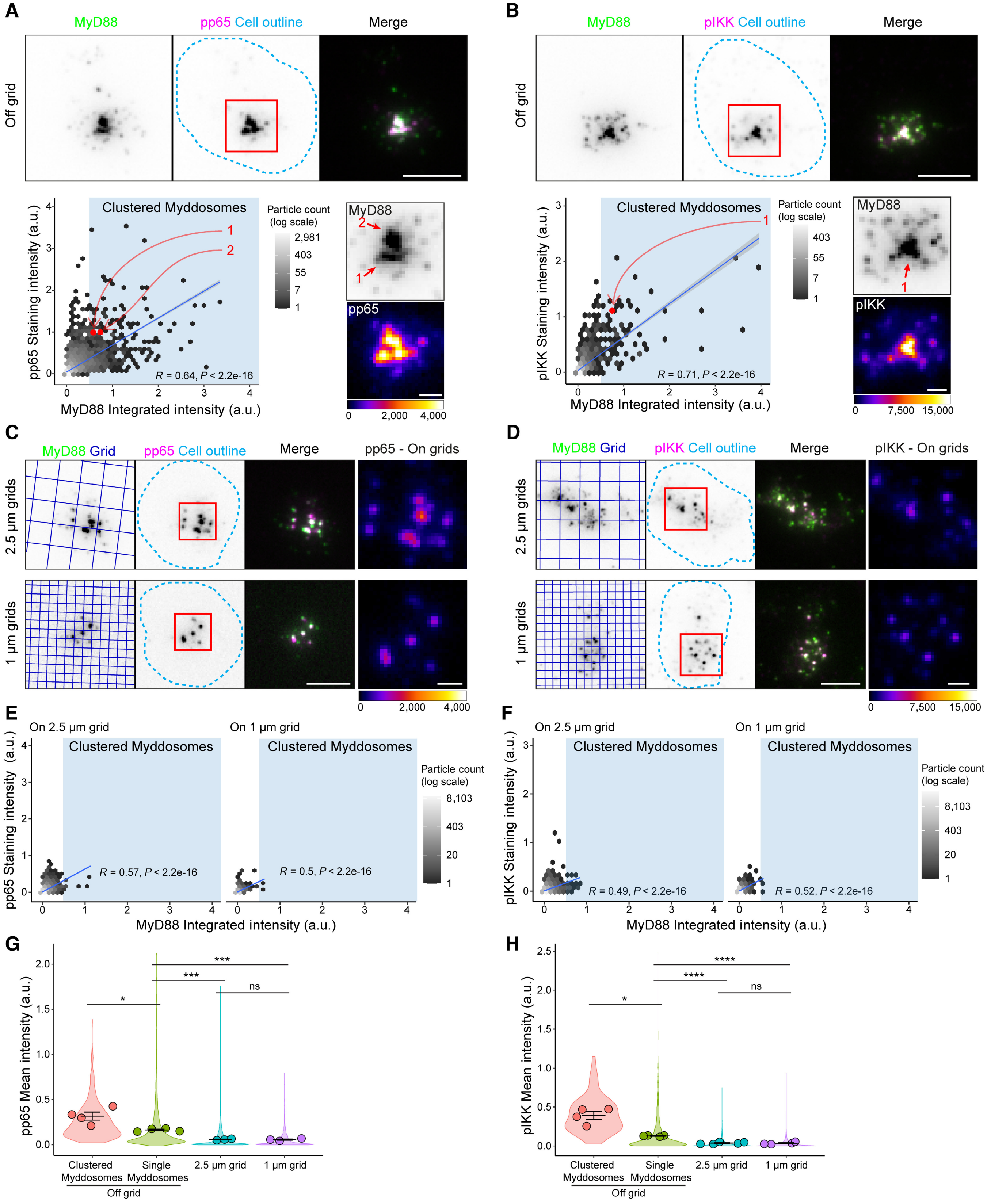
A
MyD88
pp65 Cell outline
Merge
Off grid
Clustered Myddosomes
Particle count (log scale)
2,981
403
55
7
1
MyD88
pp65
R = 0.64, P < 2.2e-16
pp65 Staining intensity (a.u.)
MyD88 Integrated intensity (a.u.)
0 2,000 4,000
B
MyD88
pIKK Cell outline
Merge
Off grid
Clustered Myddosomes
Particle count (log scale)
403
55
7
1
MyD88
pIKK
R = 0.71, P < 2.2e-16
pIKK Staining intensity (a.u.)
MyD88 Integrated intensity (a.u.)
0 7,500 15,000
C
MyD88 Grid
pp65 Cell outline
Merge
pp65 - On grids
2.5 µm grids
1 µm grids
0 2,000 4,000
D
MyD88 Grid
pIKK Cell outline
Merge
pIKK - On grids
2.5 µm grids
1 µm grids
0 7,500 15,000
E
On 2.5 µm grid
On 1 µm grid
Clustered Myddosomes
R = 0.57, P < 2.2e-16
R = 0.5, P < 2.2e-16
Particle count (log scale)
8,103
403
20
1
pp65 Staining intensity (a.u.)
MyD88 Integrated intensity (a.u.)
F
On 2.5 µm grid
On 1 µm grid
Clustered Myddosomes
R = 0.49, P < 2.2e-16
R = 0.52, P < 2.2e-16
Particle count (log scale)
8,103
403
20
1
pIKK Staining intensity (a.u.)
MyD88 Integrated intensity (a.u.)
G
pp65 Mean intensity (a.u.)
*
***
***
ns
Clustered Myddosomes
Single Myddosomes
2.5 µm grid
1 µm grid
Off grid
H
pIKK Mean intensity (a.u.)
*
****
****
ns
Clustered Myddosomes
Single Myddosomes
2.5 µm grid
1 µm grid
Off grid

Figure 3.

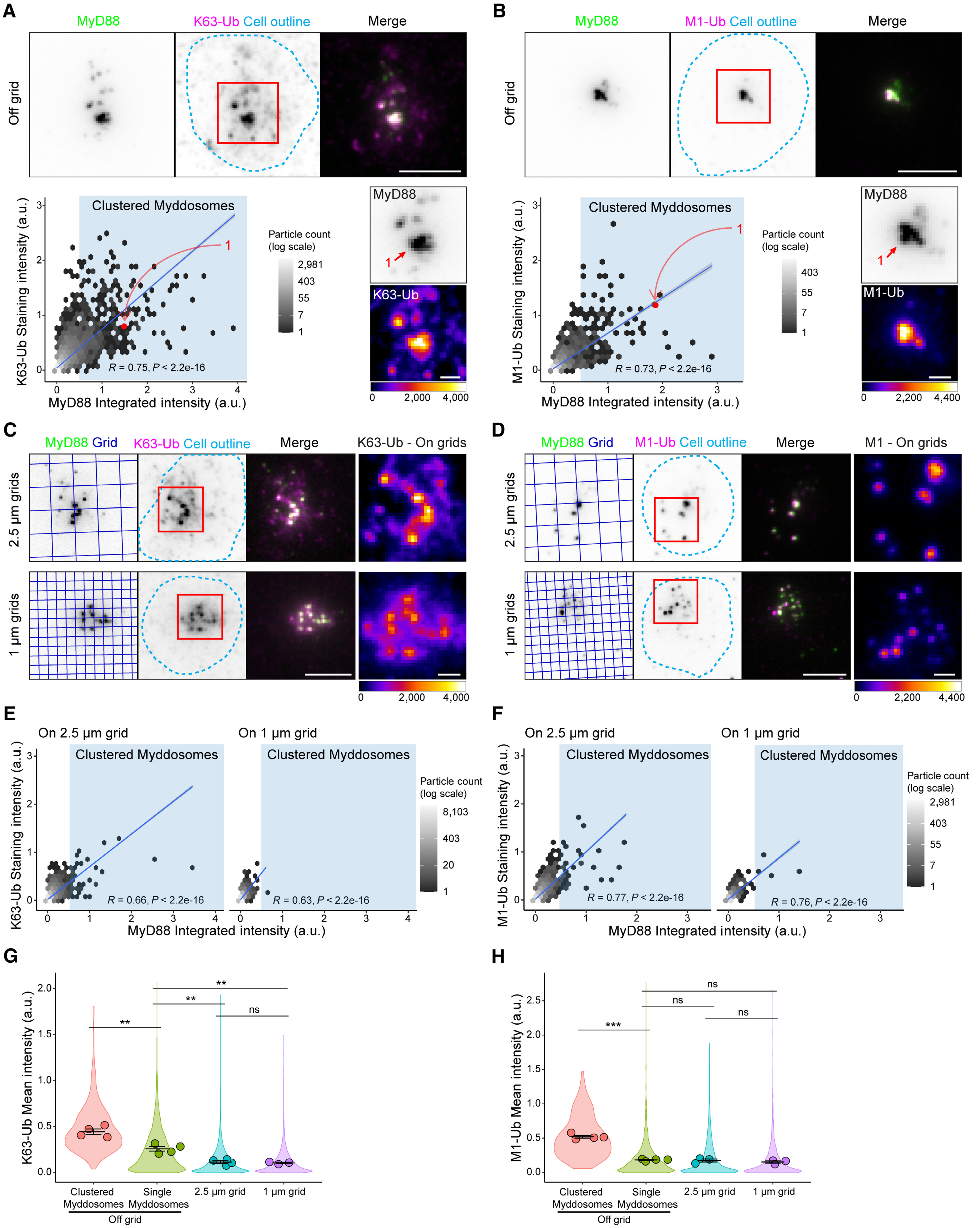

Figure 4.

© 2023 The Authors

Figure 4. Comparison of Myddosome K63-Ub and M1-Ub antibody staining on and off nanopatterned grids.

A, B Top, TIRF images of fixed EL4-MyD88-GFP cells incubated with IL-1 functionalized SLBs for 30 min and stained with anti-K63-Ub (A) or anti-M1-Ub (B). Scale bar, 5 μm. Region of interest (red box, merge image) shows an example of MyD88-GFP puncta that is colocalized with K63-Ub (A) or M1-Ub (B) puncta. Bottom, 2D histograms of the distribution of MyD88 puncta intensity and associated K63-Ub (A) or M1-Ub (B) staining intensity. Linear fit is shown as a blue line superimposed on 2D histograms (Pearson correlation coefficient, *R*, of linear fit labeled on 2D histograms). Blue-shaded region on scatter plot high MyD88 puncta classified as clustered Myddosomes. Bottom right, zoomed images of the region of interest (red box overlaid merge image, top) show MyD88-GFP channel and associated K63-Ub (A) and M1-Ub (B) channel (K63/M1-Ub images are displayed with Fire LUT). Red data points on the 2D histogram are from indicated puncta in the MyD88-GFP image (numbered red arrows). Scale bar, 1 μm.

C, D TIRF images of fixed EL4-MyD88-GFP cells incubated with partitioned IL-1 functionalized SLBs (2.5 μm top row and 1 μm bottom row grids) and stained with anti-K63-Ub (C) or anti-M1-Ub (D). Region of interest (red box overlaid merge image) shows an example of MyD88-GFP puncta that colocalize with K63-Ub (A) or M1-Ub (B) puncta. Scale bar, 5 μm. Far right, zoomed image of K63-Ub (C) or M1-Ub (D) puncta (from region of interest overlaid merge image) displayed with Fire LUT. Scale bar, 1 μm.

E, F 2D histograms of the distribution of MyD88 puncta intensity and associated K63-Ub (E) or M1-Ub (F) staining intensity on 2.5 and 1 μm grids. Linear fit is shown as a blue line superimposed on 2D histograms (Pearson correlation coefficient of linear fit labeled on 2D histograms).

G, H Quantification of mean K63-Ub (G) or M1-Ub (H) staining intensity for puncta classifieds as single or clustered Myddosomes, and MyD88 puncta formed on 2.5 and 1 μm grids. The normalized mean intensity for clustered, single Myddosomes, and MyD88 puncta on 2.5 and 1 μm grids are the following: for K63-Ub 0.444 ± 0.030, 0.257 ± 0.025, 0.113 ± 0.015 and 0.104 ± 0.008; for M1-Ub 0.520 ± 0.020, 0.183 ± 0.008, 0.174 ± 0.022 and 0.153 ± 0.016 (a.u., mean ± SEM, mean value states in the order they appear on plot, left to right). Violin plots show the distribution of all segmented MyD88 puncta. Data points superimposed on the violin plots are the averages from independent experiments. *P*-values are ** < 0.01, *** < 0.001. Bars represent mean ± SEM (n = 3–4 biological replicates for K63, with 14,571, 27,494 and 24,026 puncta off grid, on 2.5 and 1 μm grid measured in total across all replicates; n = 3–4 biological replicates for M1, with 3,114, 6,091 and 1,844 puncta off grid, on 2.5 and 1 μm grid measured in total across all replicates). Statistical significance is determined using unpaired two-tailed Student's *t*-test.

Source data are available online for this figure.

grid had statistically greater K63-Ub staining intensity than on-grid Myddosomes (Fig 4G). In contrast, single Myddosomes on and off grids had no statistical difference in mean M1-Ub staining intensity (Fig 4H). Therefore, similar to pp65 and pIKK, the localized generation of K63-Ub and M1-Ub at the cell surface increases proportionally with the nanoscale density of Myddosomes. However, the generation of M1-Ub was specifically enhanced at Myddosome clusters.

In summary, we found that signaling outputs such as pp65, pIKK and K63/M1-Ub colocalize with single and clustered Myddosome complexes. This suggests that a single Myddosome can activate NF-kB signalosome formation and this is an invariant signaling output encoded by Myddosome assembly. However, we found Myddosome clusters colocalized with > 3-fold larger NF-kB signalosomes defined by greater amounts of pp65, pIKK, and K63/M1-Ub. Furthermore, the degree of clustering led to a proportional increase in signalosome size and the robust incorporation of M1-Ub into this NF-kB signalosome. The impact of clustering on signal transduction is apparent when we calculate the K63/M1-Ub, pp65, and pIKK staining intensity per single Myddosome within clusters and compare that to isolated Myddosome complexes. We conclude that the signaling output of single Myddosome complexes increases when organized within clusters (Fig EV2A–D). This suggests that the spatial organization of the Myddosome encodes an analog signaling response.

Larger Myddosome clusters have enhanced TRAF6 and LUBAC recruitment

One limitation of immunofluorescence analysis (Figs 3 and 4) is that the spatial-temporal relationship between Myddosome formation, clustering, and NF-kB signalosome formation cannot be resolved. Therefore, having shown that Myddosomes can generate invariant and proportional outputs, we examined how these outputs arose from the dynamics of Myddosome formation, clustering and NF-kB signalosome assembly. Our data (Figs 2–4), along with published studies (Tarantino *et al*, 2014; Du *et al*, 2022), suggest that NF-kB activation occurs in condensate cellular compartments that contain K63-Ub/M1-Ub chains. We generated two CRISPR double knock-in EL4 cell lines that expressed MyD88-GFP and either the K63-Ub E3 ligase TRAF6 or the M1-Ub E3 ligase LUBAC subunit HOIL1 labeled with the mScarlet (Appendix Figs S2A–C and S3A–D). When we imaged these cell lines, we found that a subset of MyD88-GFP puncta recruited mScarlet-TRAF6 (Fig 5A) or mScalet-HOIL1 (Fig 5B). We found that TRAF6 or HOIL1 appeared after the formation of the MyD88 puncta (Fig 5A and B, Movies EV3 and EV4). Both MyD88-GFP and mScarlet-TRAF6 or mScarlet-HOIL1 puncta were initially dim and grew in intensity (Fig 5A and B). In some instances, we observed that TRAF6 was transiently recruited to Myddosomes, with this transient TRAF6 recruitment often preceding the stable association of TRAF6 with MyD88 (Fig EV3A). This dynamic suggests that Myddosome served as a scaffold for the nucleation of TRAF6 assemblies (Yin *et al*, 2009). In summary, we found that Myddosomes recruit and assemble punctate structures of the ubiquitin ligases TRAF6 and LUBAC. The molecular dynamics of MyD88 and the E3 ligases TRAF6 or LUBAC are consistent with Myddosomes functioning as an inducible scaffold and focal point for the activation of K63/M1-Ub generation.

We asked whether MyD88-GFP puncta clustering and lifetime enhanced TRAF6 recruitment. We observed that TRAF6-positive MyD88 puncta had an average size of 10.4× MyD88s (Fig EV3B and C). Based on structural studies of 6× MyD88s per Myddosome (Lin *et al*, 2010a), the average MyD88 copy number in TRAF6-positive puncta suggested they contain on average one or more Myddosome complexes. In total, 15.4 ± 2.4% of MyD88 puncta colocalize with TRAF6 (mean ± SEM, from six replicates, Fig 5C, All); however, when we normalized MyD88 puncta intensity to the number Myddosomes per puncta (see Materials and Methods, and Fig EV3B), we found that 58.4 ± 5.9% of Myddosome clusters were TRAF6-positive (Fig 5C, ≥ 2). In comparison, the percentage of TRAF6-positive puncta was 6.1 ± 1.8% and 36.2 ± 5.2% for puncta containing ≤ 1 or > 1 Myddosome complexes (mean ± SEM,

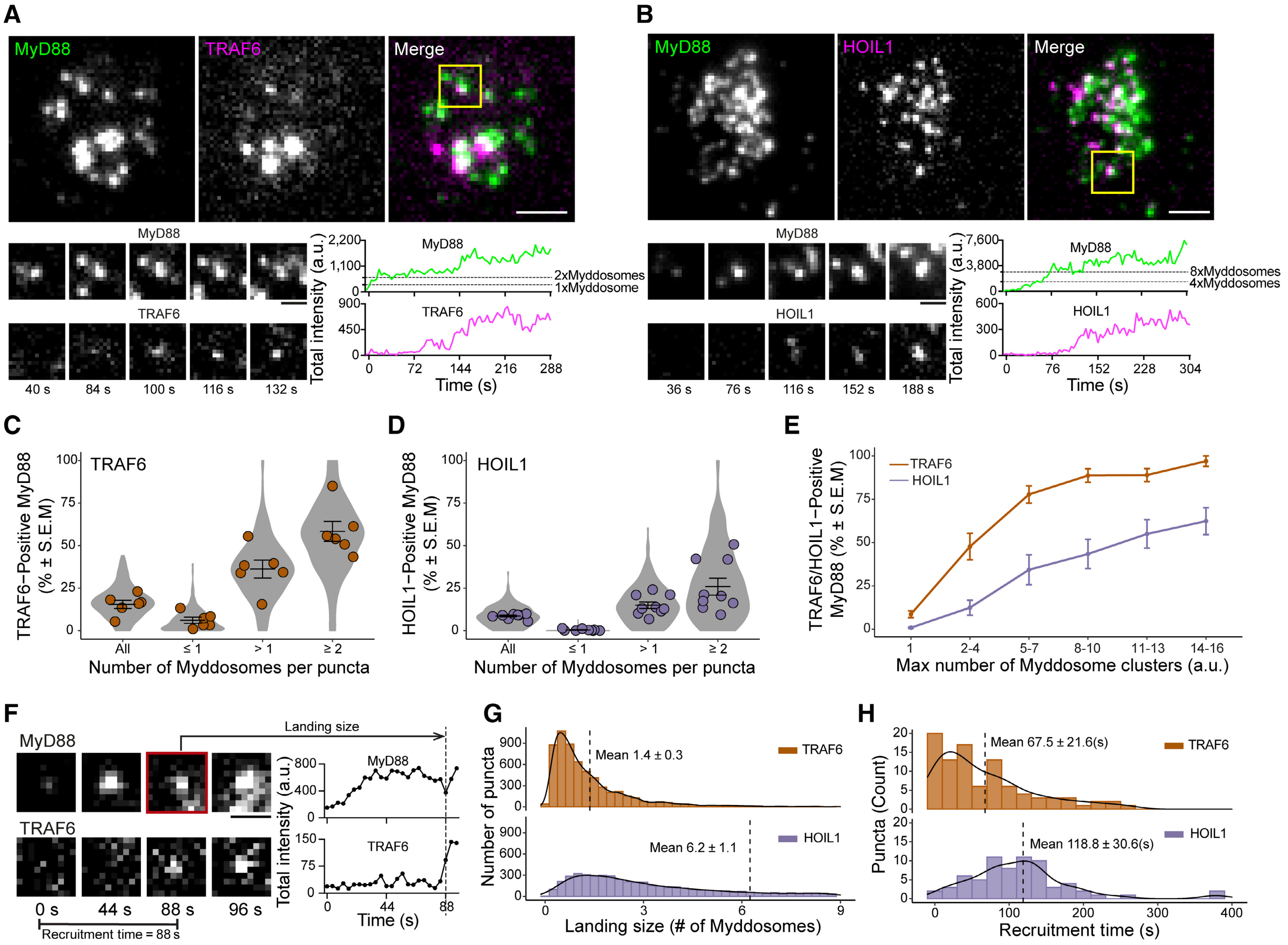

Figure 5. E3 ligases TRAF6 and HOIL1 are recruited to Myddosomes.

A, B Top: TIRF images of MyD88-GFP and mScarlet-TRAF6 (A) or mScarlet-HOIL1 (B). Region of interest (yellow box, merge image) shows an example of a MyD88-GFP puncta colocalized with mScarlet-TRAF6 (A) or mScalet-HOIL1 (B). Bottom: Time-series TIRF images from the region of interest (left) and fluorescence intensity times series (right) of MyD88 and TRAF6 (A) or HOIL1 (B).

C, D Quantifications of percentage of MyD88-GFP puncta that colocalized with TRAF6 (C) or HOIL1 (D) grouped for all puncta, puncta containing ≤ 1×, > 1×, and ≥ 2× Myddosome complexes. The percentages for TRAF6 (C) in these groups are 15.4 ± 2.4%, 6.1 ± 1.8%, 36.2 ± 5.2%, and 58.4 ± 5.9%, respectively (mean ± SEM). The percentages for HOIL1 (D) in these groups are 8.6 ± 0.5%, 0.4 ± 0.2%, 14.9 ± 1.9%, and 25.9 ± 5.0%, respectively (mean ± SEM). Violin plots indicate the distribution of individual cell measurements. Colored dots superimposed on the violin plots are the averages from independent experiments. Bars represent mean ± SEM (n = 6 biological replicates for TRAF6, with 191 cells measured in total across all replicates, see also Appendix Fig S4A; n = 9 biological replicates for HOIL1, with 230 cells measured in total across all replicates, see also Appendix Fig S4B).

E Quantifications of the percentage of TRAF6- or HOIL1-positive MyD88 puncta on single Myddosomes and clusters containing 2–4, 5–7, 8–10, 11–13, and 14–16 Myddosome complexes. With greater Myddosome numbers per puncta, the percentage of MyD88 colocalized with TRAF6 or HOIL1 increases. The data points represent the average across replicates and bars represent mean ± SEM (n = 6 biological replicates for TRAF6, with 191 cells measured in total across all replicates, see also Appendix Fig S4A; n = 9 biological replicates for HOIL1, with 230 cells measured in total across all replicates, see also Appendix Fig S4B).

F Analysis of TRAF6 and HOIL1 landing size and recruitment time to MyD88 puncta. Left, time-series TIRF images showing MyD88-GFP puncta nucleation and the appearance of TRAF6. Right, the associated fluorescence intensity time trace for the time series shown. Recruitment time is defined as the time interval from Myddosome nucleation (e.g., time = 0 s when MyD88-GFP puncta appears) to the appearance of a TRAF6 or HOIL1 puncta. Landing size is defined as the fluorescent intensity of the MyD88 puncta at the time when TRAF6 or HOIL1 appears (indicated on the fluorescent intensity trace with arrow).

G Histogram of landing size of MyD88 puncta, expressed as number of Myddosome complexes per puncta, for TRAF6 (top, n = 6,015 recruitment events, technical replicates, from 183 cells pooled from six biological replicates) and HOIL1 (bottom, n = 5,562 recruitment events, technical replicates, from 212 cells pooled from nine biological replicates) recruitment. Histogram is overlaid with a density plot of the distribution. Black horizontal lines on the histograms denote the average landing size (mean ± SEM).

H Histogram for the recruitment time of TRAF6 (top, n = 94 recruitment events, technical replicates, from four cells pooled from three biological replicates) and HOIL1 (bottom, n = 69 recruitment events, technical replicates, from four cells pooled from four biological replicates) overlaid with the density plot of the distribution. Black horizontal lines on the histograms denote the average recruitment time (mean ± SD).

Source data are available online for this figure.

 © 2023 The Authors

from 6 replicates, Fig 5C and Appendix Fig S4A). We analyzed the relationship between lifetime and TRAF6 recruitment. Using a threshold of 50 s to define long-lived MyD88 puncta, we found that 34.6 ± 3.5% MyD88 with lifetimes ≥ 50 s colocalized with TRAF6 versus 7.1 ± 1.3% of puncta with lifetime < 50 s (mean ± SEM, from six replicates, Fig EV3D). In summary, stable Myddosome clusters are more likely to recruit TRAF6.

We applied the same analysis to investigate HOIL1 recruitment. We found that HOIL1-positive MyD88 puncta were greater in size than noncolocalized puncta (47.4 MyD88s for positive puncta versus 11.6 MyD88s for negative puncta, Fig EV3E). 19.3 ± 1.3% of MyD88 puncta with a lifetime ≥ 50 s colocalized with HOIL1. In comparison, only 2.5 ± 0.2% of MyD88 puncta with lifetime < 50 s colocalized with HOIL1 (Fig EV3F). In summary, like observed for TRAF6, MyD88 puncta that recruit HOIL1 were more likely to be larger puncta with longer lifetimes. We investigated the role of Myddosome clustering in HOIL1 recruitment. We found that on average 8.6 ± 0.5% of all MyD88-GFP puncta colocalized with HOIL1 (mean ± SEM, from 9 replicates, Fig 5D, Appendix Fig S4B). When we quantified multicomplex puncta containing > 1 or ≥ 2 Myddosomes, we found that the percent of HOIL1-positive recruitment increased to 14.9 ± 1.9% and 25.9 ± 5.0%, respectively (mean ± SEM, Fig 5D).

Finally, we plotted the percent of MyD88 puncta that colocalized with TRAF6 or HOIL1 as a function of the number of Myddosome complexes per puncta (Fig 5E). We found that the percent of TRAF6 and HOIL1 colocalized puncta increased as the number of Myddosomes per MyD88-GFP puncta increased (Fig 5E). We observed a dramatic change from single Myddosomes to small clusters, estimated to contain 2–4 complexes, which increased the probability of TRAF6 and HOIL1 recruitment by 5- and 10-fold, respectively. The probability of TRAF6/HOIL1 recruitment continued to increase with the increasing density of Myddosomes per puncta. Therefore, Myddosomes organized into clusters have a greater probability of recruiting E3 ubiquitin ligases TRAF6 and HOIL1. We conclude that this enhanced recruitment is the mechanistic basis for why K63-Ub/M1-Ub responses scale with the density of Myddosome complexes within clusters (Fig 4).

Myddosome clustering triggers the sequential recruitment of TRAF6 and LUBAC

If clustering is a driver for TRAF6 and HOIL1 recruitment, we expect that formation of clusters would precede the recruitment of both ligases. Therefore, we asked whether the formation of Myddosome clusters occurs before or after the recruitment of TRAF6 and HOIL1. We analyzed the size of Myddosomes at the time point when TRAF6 and HOIL1 are recruited. We defined this time point as the TRAF6/HOIL1 landing size (Fig 5F) and quantified the number of Myddosomes per puncta at this time point. We found that the average landing size for TRAF6 was 1.4 ± 0.3 Myddosome complexes and for HOIL1 the landing size was 6.2 ± 1.1 Myddosome complexes per puncta (Fig 5G). This suggests that on average, the formation of Myddosome clusters precedes the recruitment of TRAF6 and LUBAC.

We analyzed the recruitment time of TRAF6 and HOIL1, which we defined as the time interval from the nucleation of a MyD88 puncta to the recruitment of mScarlet-TRAF6 or mScarlet-HOIL1 (Fig 5H). We found that the average recruitment time for TRAF6 was 67.5 ± 21.6 s (mean ± SD, Fig 5H). In contrast, HOIL1 had an average recruitment time of 118.8 ± 30.6 s (mean ± SD, Fig 5H). We conclude that TRAF6 and HOIL1 are recruited to clusters of Myddosomes and that the recruitment of these two ubiquitin ligases is staggered temporally: TRAF6 is recruited first followed by HOIL1. In contrast to TRAF6, HOIL1 is recruited to puncta composed of a greater density of Myddosome complexes. In conclusion, Myddosome signaling outputs are kinetically controlled by spatial organization. Specifically, the analog production of signaling outputs is encoded by Myddosome density within clusters as this regulates the probability of TRAF6 and HOIL1 recruitment.

TRAF6 and LUBAC have enhanced recruitment and lifetime at Myddosome clusters

We set out to assay how the combination of nanopattern grids and ligand density affected Myddosome clustering and TRAF6/HOIL1 recruitment. If clustering regulated the probability of TRAF6/LUBAC recruitment (Fig 5E–G) and this was the basis of digital and analog Myddosome signaling outputs, we reasoned inhibiting clustering and isolating single complexes should reduce the recruitment of both E3 ligases. We predicted that increasing IL-1 density within individual 1 μm^2 corrals would restore TRAF6/HOIL1 recruitment, as single corrals would contain sufficient IL-1 to trigger the assembly of multiple Myddosomes that could merge into clusters.

We characterized the formation of Myddosome clusters in cells on and off 1 μm grids stimulated by SLBs with 1 and 10 IL-1/μm^2 (Fig EV4A and B). We confirmed that on 1 μm grids, Myddosome cluster formation was fourfold greater at the higher ligand density (4.7% versus 1.2% of puncta classified as clusters at 10 and 1 IL-1/μm^2, Fig EV4A and B). As observed previously (Fig 1F), we found that MyD88 puncta size in cells stimulated with 1 μm grids is smaller than those stimulated with SLBs off grid. Live-cell imaging and kymograph analysis at 1 IL-1/μm^2 showed that, in contrast to off-grid (Fig 6A), MyD88-GFP puncta on 1 μm grids did not coalesce and cluster (Fig 6B). However, a portion of these puncta still recruited mScarlet-TRAF6 (Fig 6B, Movie EV5). Analysis revealed a twofold difference off and on grids in the frequency of TRAF6 recruitment at 1 IL-1/μm^2 (16.2 ± 2.5% versus 6.1 ± 1.5% TRAF6-positive MyD88 puncta per cell off and on grids, respectively, mean ± SEM, Fig 6C and Appendix Fig S5A). At a higher ligand density of 10 IL-1/μm^2, the percentage of TRAF6-positive Myddosome on 1 μm grids increased by a factor of 2 (12.7 ± 1.8% versus 6.1 ± 1.5% TRAF6-positive MyD88 puncta per cells at 10 and 1 IL-1/μm^2, Fig 6C–F and Appendix Fig S5A, Movie EV6). Thus, increasing the number of IL-1 per 1 μm^2 corral can rescue the perturbation of TRAF6 recruitment. These data reveal that single Myddosomes can trigger a TRAF6 response, consistent with an invariant signaling output. However, Myddosome clusters have an increased probability of TRAF6 recruitment, suggesting a mechanism for how clusters can generate a proportionally greater signaling output than single complexes.

To examine the role of Myddosome clusters on HOIL1 recruitment, we applied the same strategy of using 1 μm grids and a high and low ligand density to change the frequency of cluster formation (Fig EV5A and B). At a ligand density of 10 IL-1/μm^2, we observed the dynamic coalescence and clustering of MyD88 puncta (Fig 6G,

© 2023 The Authors

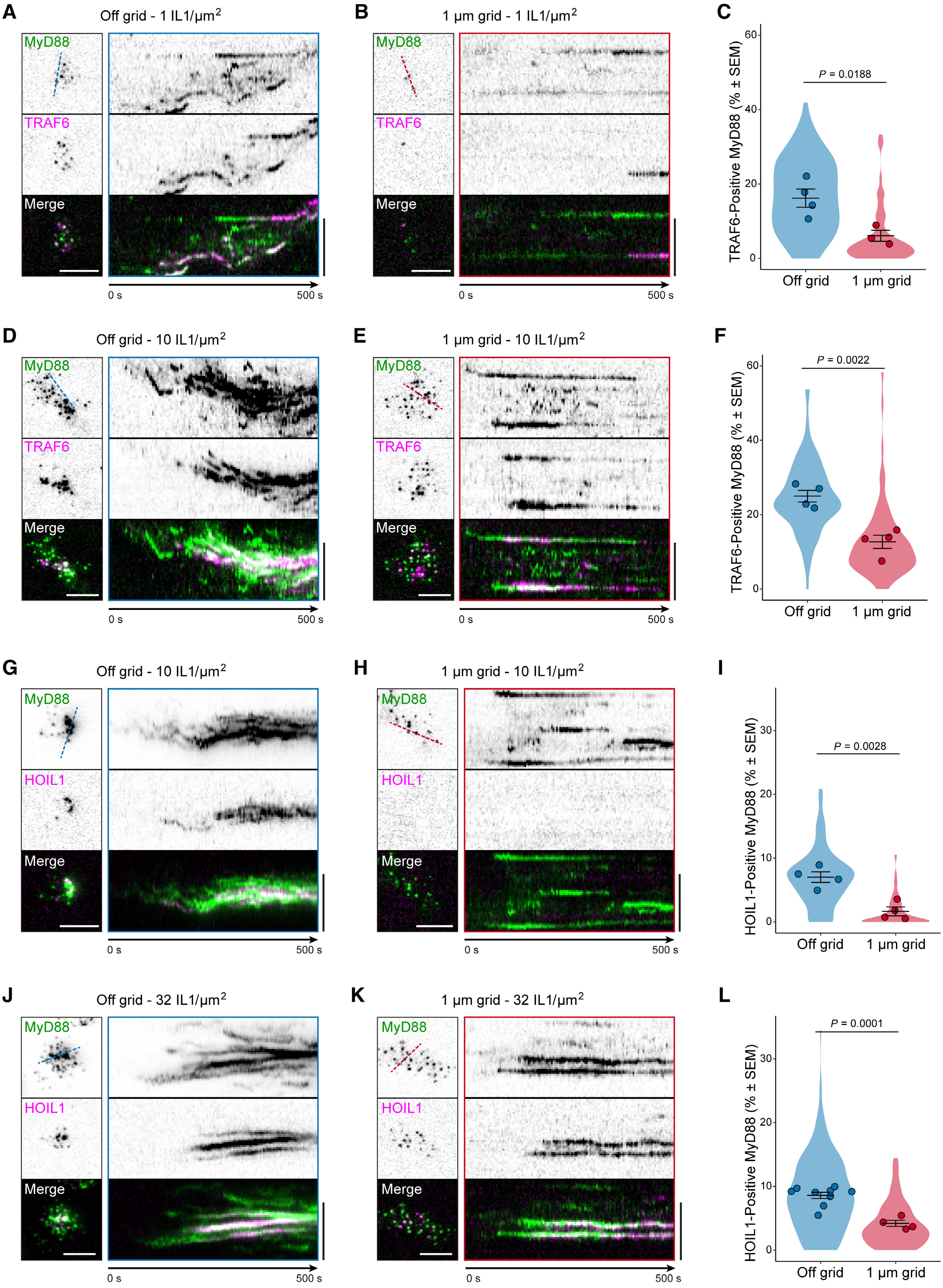

Figure 6.

© 2023 The Authors

Figure 6. Extracellular barriers that restrict Myddosome clustering reduce TRAF6 and HOIL1 recruitment.

A, B TIRF images of EL4 cells expressing MyD88-GFP and mScarlet-TRAF6 stimulated on IL-1 functionalized SLBs at a ligand density of 1 IL-1/μm^2 off grids (A) or on 1 μm grids (B). Kymographs derived from dashed lines overlaid TIRF images (left panel). Scale bars, 5 μm.

C Quantification of percentage of MyD88-GFP puncta that colocalized with TRAF6 off grids and on 1 μm grids at a ligand density of 1 IL-1/μm^2, and the percentages are 16.2 ± 2.5% and 6.1 ± 1.5%, respectively (mean ± SEM). Violin plots indicate the distribution of individual cell measurements. Colored dots superimposed on the violin plots are the averages from independent experiments. Bars represent mean ± SEM (n = 4 experimental replicates off grids, with a total of 24,315 MyD88 puncta from 91 cells; n = 3 biological replicates on 1 μm grids, with a total of 23,161 MyD88 puncta from 70 cells). Statistical significance is determined using unpaired two-tailed Student's *t*-test.

D, E TIRF images of EL4 cells expressing MyD88-GFP and mScarlet-TRAF6 stimulated on IL-1 functionalized SLBs at a ligand density of 10 IL-1 per μm^2 off grids (D) or on 1 μm grids (E). Kymographs derived from dashed lines overlaid TIRF images (left panel). Scale bars, 5 μm.

F Quantification of percentage of MyD88-GFP puncta that colocalized with TRAF6 off grids and on 1 μm grids at a ligand density of 10 IL-1/μm^2, and the percentages are 25.0 ± 1.6% and 12.7 ± 1.8%, respectively (mean ± SEM). Violin plots indicate the distribution of individual cell measurements. Colored dots superimposed on the violin plots are the averages from independent experiments. Bars represent mean ± SEM (n = 4 biological replicates off grids, with a total of 34,452 MyD88 puncta from 87 cells; n = 4 biological replicates on 1 μm grids, with a total of 71,525 MyD88 puncta from 100 cells). Statistical significance is determined using unpaired two-tailed Student's t-test.

G, H TIRF images of EL4 cells expressing MyD88-GFP and mScarlet-HOIL1 stimulated on IL-1 functionalized SLBs at a ligand density of 10 IL-1/μm^2 off grids (G) or on 1 μm grids (H). Kymographs derived from dashed lines overlaid TIRF images (left panel). Scale bars, 5 μm.

I Quantification of percentage of MyD88-GFP puncta that colocalized with HOIL1 off grids and on 1 μm grids at a ligand density of 10 IL-1/μm^2, and the percentages are 7.0 ± 0.8% and 1.7 ± 0.7%, respectively (mean ± SEM). Violin plots indicate the distribution of individual cell measurements. Colored dots superimposed on the violin plots are the averages from independent experiments. Bars represent mean ± SEM (n = 4 biological replicates off grids, with a total of 53,852 MyD88 puncta from 74 cells; n = 4 biological replicates on 1 μm grids, with a total of 55,075 MyD88 puncta from 154 cells). Statistical significance is determined using unpaired two-tailed Student's *t*-test.

J, K TIRF images of EL4 cells expressing MyD88-GFP and mScarlet-HOIL1 stimulated on IL-1 functionalized SLBs at a ligand density of 32 IL-1/μm^2 off grids (J) or on 1 μm grids (K). Kymographs derived from dashed lines overlaid TIRF images (left panel). Scale bars, 5 μm.

L Quantification of percentage of MyD88-GFP puncta that colocalized with HOIL1 off grids and on 1 μm grids at a ligand density of 32 IL-1/μm^2, and the percentages are 8.6 ± 0.5% and 4.2 ± 0.5%, respectively (mean ± SEM). Violin plots indicate the distribution of individual cell measurements. Colored dots superimposed on the violin plots are the averages from independent experiments. Bars represent mean ± SEM (n = 9 biological replicates off grids, with a total of 118,354 MyD88 puncta off grids from 230 cells; n = 4 biological replicates on 1 μm grids, with a total of 68,819 MyD88 puncta from 138 cells). Statistical significance is determined using unpaired two-tailed Student's *t*-test.

Source data are available online for this figure.

Movie EV7). As observed previously (Fig 5B), mScarlet-HOIL1 was recruited to these large clusters of Myddosomes. However, on 1 μm grids at 10 IL-1/μm^2, we observed that $< 2\%$ puncta recruited HOIL1 (7.0 ± 0.8% versus 1.7 ± 0.7% puncta recruited HOIL1 off grid versus on grid, see Figs 6H and I and EV5C and Appendix Fig S5B, Movie EV7). We analyzed the density of Myddosomes per HOIL1-positive MyD88 puncta on and off grids and found that the average number of Myddosomes per puncta at the point of HOIL1 recruitment was 4.4 off grid and 0.7 on 1 μm grids (Fig EV5D). These data suggest that single complexes can recruit HOIL1; however, the probability was $>$ 4-fold lower than at Myddosome clusters. When we increased ligand density, we found that MyD88 puncta readily recruited HOIL1 off and on 1 μm grids (8.6 ± 0.5% versus 4.2 ± 0.5% HOIL1-positive MyD88 puncta off and on 1 μm grids, respectively, Fig 6J–L, also see Appendix Fig S5B, Movie EV8). At 32 IL-1/μm^2, the HOIL1 landing size was 6.2 versus 1.4 Myddosome complexes per puncta off versus on grid, respectively (Fig EV5E), suggesting that in both experimental regimes, HOIL1 is recruited more readily to Myddosome clusters. In summary, we can control the association of LUBAC with Myddosome complexes by controlling the formation of clusters (Fig 2D). Therefore, Myddosomes clustering is a key driver of LUBAC recruitment and upregulating M1-Ub production (Fig 4H).

We examined the lifetime of TRAF6 and HOIL1 recruitment to single and clustered Myddosomes from on- and off-grid data (Fig 7A and B). We find that a greater density and number of Myddosome complexes within clusters correlates with a greater lifetime of TRAF6 and HOIL1. We conclude that Myddosome clusters increase the stability of TRAF6 and HOIL1 at Myddosomes and that this is the possible basis for why clusters have increased signaling output (Fig 7C). Our results show that Myddosome clustering and the sequential recruitment of TRAF6 and then HOIL1 (Fig 5H) is a potential mechanism to generate signaling outputs proportional to the stimulation level (Fig 7C).

Discussion

Here, we used high-resolution microscopy to visualize and quantify the signaling output of Myddosomes. We find single Myddosomes can recruit TRAF6 and HOIL1 (Fig 5) and form a signalosome that promotes the localized production of K63/M1-Ub, pIKK and pp65 (Figs 3 and 4). This suggests that Myddosomes function as a scaffold to stimulate the formation of a NF-kB activating signalosome (Fig 2A and B). We conclude that NF-kB signalosome formation is a digital signaling response of Myddosomes (Fig 7C). However, the probability of activating this response at single complexes is low, but we find that this signaling response can be amplified by increasing local density of Myddosomes within cell surface clusters. We and others have observed that Myddosomes cluster (Latty *et al*, 2018). Here, we use extracellular nanoscale barriers to reveal that Myddosomes are tethered to the cell surface via direct interaction with the IL-1R bound to extracellular IL-1 (Fig 1B and C). Using this technology, we discover the reorganization of Myddosomes into clusters has functional consequences: These Myddosome clusters increase the nucleation frequency and signaling output of this NF-kB signalosome (Fig 2). Myddosome clustering dramatically enhances the recruitment and incorporation of LUBAC into these signalosomes. These results suggest that the spatial organization of Myddosomes can encode responses proportional to the amount of IL-1 stimulation.

Previous studies have found that Myddosomes form large aggregate structures after TLR or IL-1 stimulation (Latz *et al*, 2002; Latty *et al*, 2018; Deliz-Aguirre *et al*, 2021). In macrophages stimulated with TLR4 agonist LPS, the formation of large Myddosome clusters correlated with higher doses of LPS stimulation enhanced NF-kB activation and gene expression (Latty *et al*, 2018). These results are

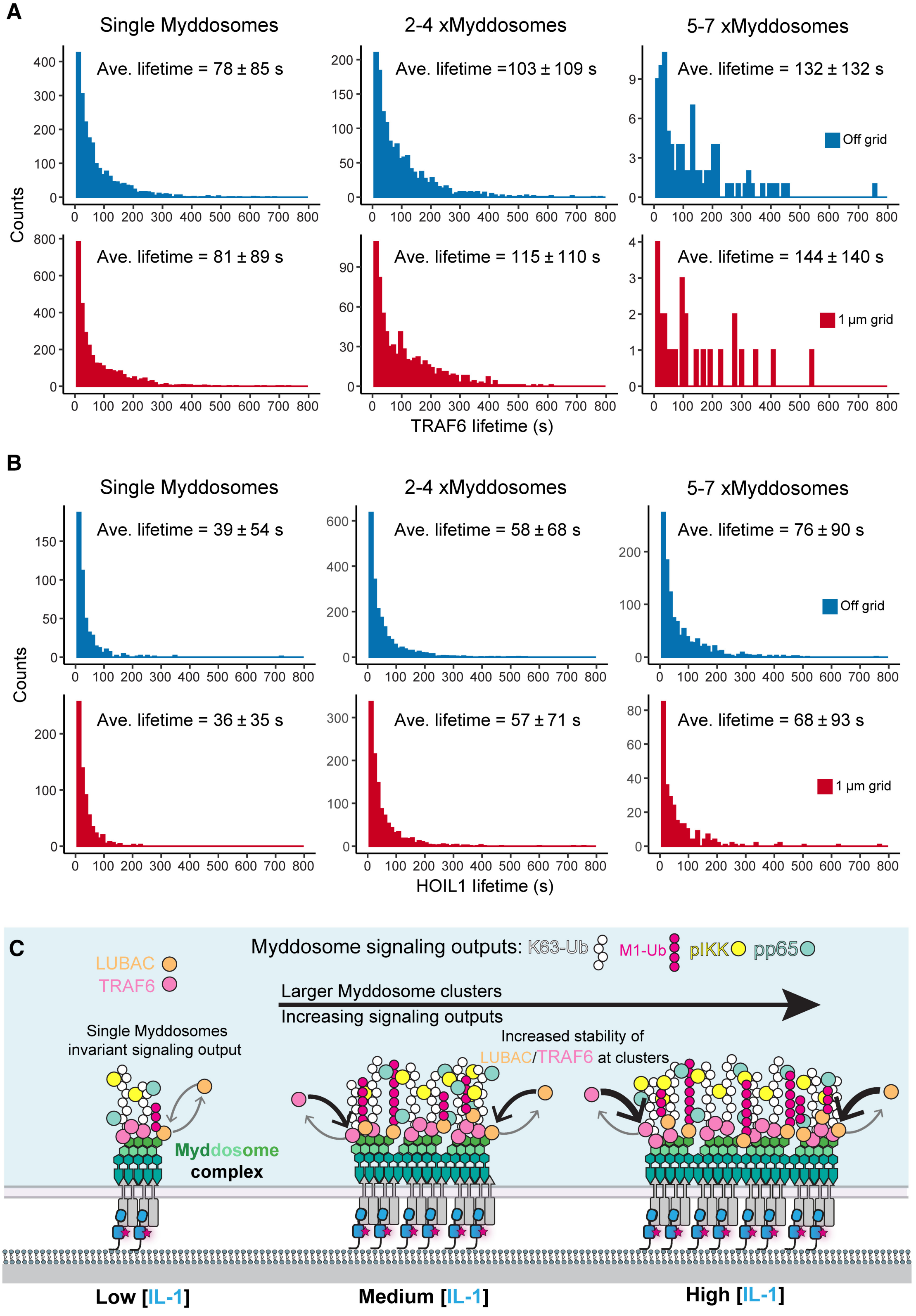

Figure 7.

 © 2023 The Authors

Figure 7. Clustering increases TRAF6 and HOIL1 lifetime at Myddosomes.

A, B Histogram showing the lifetime of TRAF6 and HOIL1 recruitment to single Myddosomes, and clusters containing 2–4 and 5–7 Myddosome complexes, off (blue) and on (red) 1 μm grids.

C Model showing how a single Myddosome has a digital signaling output. However, the amplitude of this output increases proportionally as the density of Myddosome complexes increases within clusters. The increased amplitude of the M1/K63-Ub, pIKK, and pp65 signaling output is likely due to the increase in stability of LUBAC and TRAF6 at larger clusters. As Myddosomes are biochemically coupled to extracellular IL-1, this mechanism examines how IL-1 signaling can generate both digital and analog signaling responses that are proportional to the stimulating dose of IL-1.

Source data are available online for this figure.

consistent with our finding that inhibiting the formation of Myddosome clusters reduces RelA nuclear translocation (Fig 1G). Here, we find evidence that clusters of Myddosomes enhance NF-kB signaling by increasing the probability of TRAF6 and, in particular, LUBAC recruitment (Fig 5C–E). We showed that the number of Myddosome clusters increases with higher IL-1 stimulation (Figs EV4 and EV5A and B) and that Myddosomes are biochemically coupled to IL-1:IL-1R complexes (Fig 1). Therefore, Myddosome clustering "reads out" the amount of IL-1 stimulation and converts it into proportional TRAF6 and HOIL1 recruitment (Fig 6).

Myddosome clusters may have other functional roles beyond enhancing NF-kB signalosome formation. Like previous studies in macrophages (Latty *et al*, 2018), we observe that Myddosomes tend to form a large central focal point (Figs 1A and 2A). As experiments with the nano grids demonstrate (Fig 7), Myddosome clusters can form on grids at higher IL-1 densities, and these clusters can recruit HOIL1 and TRAF6. Despite this rescue of TRAF6/HOIL1 recruitment, the grid still prevents the formation of this large patch-like structure. This structure observed in cells off grid (Fig 1A) possibly plays a role in other downstream signaling processes, such as the internalization of IL-1R-Myddosome complexes by endocytosis or terminating signal transduction. How the spatial organization of Myddosomes regulates other signaling reactions and cellular processes is an avenue for future investigations.

How does clustering enhance TRAF6 and HOIL1 recruitment? The Myddosome has a fixed stoichiometry (Motshwene *et al*, 2009; Lin *et al*, 2010a), and with 4× IRAK1 monomers per complex, it has a maximum of 12× TRAF6-binding motifs per complex (Ye *et al*, 2002). Therefore, clustering might be a dynamic mechanism to increase the avidity of TRAF6-binding sites at a focal point on the plasma membrane. We show that single Myddosomes can still recruit TRAF6 to the cell surface (Fig 6A), although at a lower probability than clusters of Myddosomes. TRAF6 is predicted to form a 2D lattice (Yin *et al*, 2009), with the trimeric C terminus making contact with the Myddosome (Ye *et al*, 2002). Myddosomes clustering might stabilize higher-order assemblies of TRAF6 that promote its ubiquitin ligase activity (Yin *et al*, 2009). LUBAC component HOIP recognizes K63-Ub (Emmerich *et al*, 2013), and thus, its recruitment depends on the amount of K63-Ub chains. We find less K63-Ub associated with single Myddosomes than clustered Myddosomes (Fig 4). We also find HOIL1 and M1-Ub are especially sensitive to Myddosome clustering (Figs 4 and 6). Therefore, Myddosome clustering might lead to larger TRAF6 assemblies, enhanced ubiquitin ligase activity, a greater production of K63-Ub, and enhanced HOIL1 recruitment as well as formation of M1-Ub. Thus, the K63-Ub output of TRAF6 will scale proportionally with the density of Myddosomes within clusters.

In conclusion, Myddosomes function as a plasma membrane-associated scaffold that assembles an NF-kB activating signalosome. We show that the spatial density of the Myddosome regulates the assembly and size of this NF-kB activating compartment. This mechanism might explain how the IL-1 signaling pathway can create invariant and proportional NF-kB responses (DeFelice *et al*, 2019; Son *et al*, 2021). Other innate immune signaling pathways, such as inflammasomes and STING, use the clustering of signaling complexes to control the formation of specialized signaling compartments (Magupalli *et al*, 2020; Yu *et al*, 2021). It is possible clustering is a unifying mechanism across innate immune signaling to transmit switch-like responses and analog information such as the amount and duration of a stimulus. An important future direction is quantifying the spatial organization of other innate immune signaling complexes and how this connects to digital versus analog signaling responses. The approach we establish here that combines live-cell microscopy with technologies that enable spatial control of signaling complexes provides a powerful strategy to study how the dynamics of signaling pathways shape signaling outputs.

Materials and Methods

Reagents and Tools table

Reagent or resource	Source	Identifier
**Antibodies**		
Goat polyclonal anti-MyD88	R and D Systems	Cat# AF3109; RRID: AB_2146703
Rabbit polyclonal anti-TRAF6	Enzo	Cat# ADI-AAP-426; RRID: AB_10619296
Mouse monoclonal anti-RBCK1 (HOIL1)	Santa Cruz Biotechnology	Cat# sc-393754; RRID: NA
Rabbit polyclonal anti-GFP	ChromoTek	Cat# PABG1; RRID: AB_2749857
Mouse monoclonal anti-RFP	ChromoTek	Cat# 6g6; RRID: AB_2631395
Mouse monoclonal anti-GAPDH (6C5)	Thermo Fisher Scientific	Cat# AM4300; RRID: AB_2536381

© 2023 The Authors

Reagents and Tools table (continued)

Reagent or resource	Source	Identifier
Mouse monoclonal anti-tubulin	Abcam	Cat# ab7291; RRID: AB_2241126
Rabbit monoclonal anti-NF-κB p65	Cell Signaling Technology	Cat# 8242; RRID: AB_10859369
Anti-phospho-IKKα/β	Cell Signaling Technology	Cat# 2697; RRID: AB_2079382
Anti-phospho-p65	Cell Signaling Technology	Cat# 3033; RRID: AB_331284
Human monoclonal antilinear (M1-Ub) polyubiquitin (1F11/3F5/Y102L)	Genentech (Matsumoto *et al*, 2012)	NA
Human monoclonal anti-K63-Ub linked polyubiquitin chain	Genentech (Newton *et al*, 2008)	NA
Camelid sdAb FluoTag®-X4 anti-GFP-Atto488	NanoTag Biotechnologies GmbH	Cat# N0304-At488
Goat anti-rabbit conjugated to Alexa Fluor 647	Invitrogen	Cat# A21246
Goat anti-human conjugated to Alexa Fluor 647	Invitrogen	Cat# A21445
Nano-secondary AF568	Chromotek	Cat# srbAF568-1-100
**Bacterial and virus strains**		
BL21 Rosetta	Novagen	Cat# 70954
NEB Stable	NEB	Cat# C3040
TOP10	Thermo	Cat# C404003
**Chemicals, peptides, and recombinant proteins**		
His10-Halo-Tencon-SpycatcherV2-IL-1β	Deliz-Aguirre *et al* (2021)	
Puromycin dihydrochloride	Thermo	Cat# A1113803
Blasticidin	InvivoGen	Cat# ant-bl-05
Mouse IL-1β	Peprotech	Cat# 211-11B
JF646-HaloTag ligand	Luke Lavis, HHMI	
**Critical commercial assays**		
MycoAlert® Mycoplasma Detection Kit	Lonza	Cat# LT07-218
QuickExtract DNA Extraction Solution	Lucigen	Cat# SS000035-D2
Mouse IL-2 DuoSet ELISA	R&D Systems	DY402
**Experimental models: cell lines**		
Mouse: EL4.NOB-1	ECACC	87020408
Mouse: EL4 MyD88-GFP	Deliz-Aguirre *et al* (2021)	
Mouse: EL4 mScarlet-TRAF6/MyD88-GFP	This paper	
Mouse: EL4 mScarlet-HOIL1/MyD88-GFP	This paper	
**sgRNA sequences**		
MyD88 GFP knock-in gRNA1: CCTGCCCTGAAGATGACCCT	Deliz-Aguirre *et al* (2021)	
TRAF6_mScarlet-i knock-in gRNA: GTAAACTGTGAGAACAGCTGC	This paper	
HOIL1_mScarlet-i knock-in gRNA: GTCTTCTCGTCCATCTGGCC	This paper	
**Recombinant DNA**		
pMK	Thermo	GeneArt vector
pX330-U6-Chimeric_BB-CBh-hSpCas9	Addgene	Plasmid #42230
pMK_MyD88-GFP_HDRtemp	Deliz-Aguirre *et al* (2021)	NA
pX330-MyD88-gRNA	Deliz-Aguirre *et al* (2021)	NA
pMK_TRAF6-BlastR-T2A-mScarlet-i_HDRtemp	This paper	NA
pX330-TRAF6-gRNA	This paper	NA
pMK_HOIL1-BlastR-T2A-mScarlet-i_HDRtemp	This paper	NA
pX330-HOIL1-gRNA	This paper	NA
**Software and algorithms**		
Fiji	NIH	https://fiji.sc/
MATLAB	MathWorks	https://www.mathworks.com/

© 2023 The Authors

Reagents and Tools table (continued)

Reagent or resource	Source	Identifier
R	CRAN	https://cran.r-project.org/
ggplot2	tidyverse	https://ggplot2.tidyverse.org/
Fiji/MATLAB/R scripts from image analysis	This study	https://github.com/MJ-Taylor-Lab/Analysis-for-Ub-paper

Methods and Protocols

Cell culture

EL4.NOB1 WT (purchased from ECACC, and referred to as EL4 in the paper) and gene-edited lines (derived from this parental line) were grown in RPMI (Thermo Fisher Scientific) with 10% FBS (Biozol) supplemented with 2 mM L-glutamine. EL4.NOB1 cultures were maintained at a cell density of 0.1–0.5 × 10^6 cells/ml in 5% CO_2, 37°C. All cells were determined to be negative for mycoplasma (Lonza).

Homology-directed repair (HDR) DNA template design for CRISPR/Cas9 endogenous labeling

Plasmid DNA repair templates were designed using a pMK (Life Technologies, Carlsbad, CA) vector backbone. Silent mutations were included in the homology arms to remove sgRNA target sites and avoid Cas9 cleavage of the repair template. Homology arms were amplified from EL4 genomic DNA and assembled with DNA fragments encoding the fluorescent protein tag mScarlet-i (Bindels *et al*, 2017) and a blasticidin resistance cassette separated by a 2A sequence (Szymczak-Workman *et al*, 2012). These DNA fragments were inserted into a pMK plasmid backbone using Gibson Assembly. All HDR template plasmids were sequence verified. The HDR template to label MyD88 with GFP was described previously (Deliz-Aguirre *et al*, 2021). Full details of the HDR DNA template plasmid construction used in this study are given below (full sequences of the HDR templates given in Table EV1).

pMK-BlastR-2A-mScarlet-i-TRAF6-HDR

5′ and 3′ homology arms were designed from the mouse TRAF6 gene (ENSMUSG00000027164) covering a distance of 666 bps and 646 bps either side of the ATG start codon. BlastR-2A-mScarlet-i cassette was inserted between these homology arms and fused to the TRAF6 N terminus via a 3× Gly-Gly-Ser linker. The following primers were used for the 5′ homology arm: 5′-TTGTAAAACGACGGCCAGCACCAGACTGGGCATTTAGAAATCCACATGGC-3′ and 5′-CTTCTTGAGACAAAGGCTTGGCCATAGTAGCTCTGTTGTCAGTCGATCTTCAg-3′. 3′ homology arm: 5′-GCCAGTCGTCCAGTGACTGCTGCGC-3′ and 5′-GGAAACAGCTATGACCATGCACAAGTAGCTGAGTTTAAGGCAAATTAGAAATACTACTTTC-3′. BlastR-2A-mScarlet-i cassette: 5′-ATGGCCAAGCCTTTGTCTCAAGAAGAAT-3′ and 5′-GCAGCAGTCACTGGACGACTGGCTGGATCCGCAACTGTTCTCACAGTTAAGGAGACTGCTTCCTCCACTGCCTCC-3′. pMK vector backbone: 5′-CATGGTCATAGCTGTTTCCTTGC-3′ and 5′-CTGGCCGTCGTTTTACAACG-3′.

pMK-BlastR-2A-mScarlet-i-HOIL1-HDR

5′ and 3′ homology arms were designed from the mouse HOIL1 gene (ENSMUSG00000027466) covering a distance of 598 and 790 bps either side of the ATG start codon. A BlastR-2A-mScarlet-i cassette was inserted between these homology arms and fused to the HOIL1 N terminus via a 3× Gly-Gly-Ser linker. The following primers were used for the 5′ homology arm: 5′-GTTGTAAAACGACGGCCAGCAGAGCTAGGCGCTGCCTGGAGTC-3′ and 5′-ACTGCCTCGCCCTTGCTCACCATCTGGCCTGGCTGCGCCCATCC-3′. 3′ homology arm: 5′-GGCAGTGGAGGAAGCGACGAGAAaACCAAGAAAGGTGGGCACCG-3′ and 5′-GGAAACAGCTATGACCATGTTTTCCGGTCCTCACTCCTCTGTACCC-3′. BlastR-2A-mScarlet-i cassette: 5′-GGAGGAGAATCCCGGGCCAGTGAGCAAGGGCGAGGCAGTGATC-3′ and 5′-CTCGTCGCTTCCTCCACTGCCTCCTGAGCCACCCTT-3′. pMK vector backbone: 5′-CATGGTCATAGCTGTTTCCTTGC-3′ and 5′-CTGGCCGTCGTTTTACAACG-3′.

Generation of CRISPR/Cas9 sgRNA vectors for endogenous labeling of MyD88, TRAF6, and HOIL1

Single-guide RNAs (sgRNA) targeting ± 50 bps of the N terminus start codon of TRAF6 and HOIL1 were designed using the web-based Benchling CRISPR design tool. sgRNAs were selected for each target (see Key Resources Table for sequences), and complementary oligonucleotides designed to be ligated into Bbs1 digested *Streptococcus pyogenes* Cas9 and chimeric guide RNA expression plasmid pX330, (pX330-U6-Chimeric_BB-CBh-hSpCas9, Addgene #42230). sgRNA oligonucleotides were ordered from Integrated DNA Technologies (IDT). Complementary sgRNA oligonucleotides were 5′ phosphorylated with T4 Polynucleotide kinase, annealed, and ligated into Bbs1 digested pX330 using Quick Ligase (NEB). pX330 plasmids were transformed into *NEB Stable* competent cells. All sgRNA pX330 plasmids were sequence verified.

Generation of CRISPR/Cas9 engineered cell lines

EL4.NOB1 cells were electroporated with a pX330 Cas9/gRNA expressing vector and the pMK vector encoding the HDR template with the Neon Transfection System. EL4 cells were electroporated with the following conditions: voltage (1,080 V), width (50 ms), and number of pulses (one). For double editing of MyD88/TRAF6 or MyD88/HOIL1 gene loci, 1.5 µg of sgRNA-Cas9 and HDR template plasmids (in equal molar ratio) were electroporated simultaneously. After electroporation, cells were plated in RPMI culture medium without antibiotics for 24 h. For the selection of TRAF6 and HOIL1 edited alleles, 6 µg/ml blasticidin was added to the cell culture medium 24 h after electroporation. EL4 cells were selected in blasticidin for 48 h.

Monoclonal cell lines were generated by fluorescence-activated cell sorting (FACS). Cells were sorted using BD FACS Aria II at Deutsches Rheuma-Forschungszentrum Berlin, Flow Cytometry Core Facility. To isolate gene-edited EL4 cells, we first performed a bulk sorting of double-positive cells. This population was expanded, and single cells were sorted into 96-well plates containing culture medium with 15% EL4.NOB-1 conditioned RPMI medium.

© 2023 The Authors

The gene-edited clonal cell lines were verified using PCR, sequencing, and western blot analysis. First, genomic DNA was isolated from selected monoclonal cell lines using QuickExtract DNA Extraction Solution (Epicentre). To test for gene editing and correct insertion of mGFP/mScarlet-i cassette, PCR primers were designed to amplify a DNA fragment that contained the junctions between mGFP/mScarlet-i open reading frame, the 3′ or 5′ homology arm and the gene locus. To check whether single-cell clones were homozygous or heterozygous, we designed PCR primers that amplified a fragment containing mGFP/mScarlet-i cassette, the entire 3′ or 5′ homology arms and the junction between the homology arms and the gene locus (see Table EV1). PCR products were analyzed on a 0.8–1% agarose gel, gel extracted using Monarch Nucleic Acid Purification Kits (NEB) and submitted for Sanger Sequencing. Analysis of EL4 HOIL1-mScarlet/MyD88-GFP genomic DNA showed heterozygous editing of the HOIL gene locus and homozygous editing of the MyD88 locus.

To confirm the presence of mEGFP/mScarlet-i fusion protein, the cell clones were analyzed by western blot using specific antibodies against MyD88, TRAF6, HOIL1, and GFP or mScarlet-i (RFP). Insertion of fluorescent tags resulted in a 25 kDa increase of molecular weight in comparison with nontagged protein. As expected from the sequencing result, the HOIL1 edited cell line was expressing mScarlet-i-HOIL1 and nontagged HOIL1. The TRAF6 edited cell line was expressing mScarlet-i-TRAF6 (Appendix Fig S2, full-length western blot shown in Appendix Fig S3). Finally, all cell clones were imaged by microscopy to check for correct localization of fluorescent signals.

Assay of IL-2 release in WT and gene-edited EL4 cells

To measure IL-2 release, we used the Mouse IL-2 DuoSet ELISA kit (R&D Systems; DY402-05) following the manufacturer's protocol. First, 10^6 cells in 150 µl medium per well were seeded into a 48-well plate and allowed to settle for 30 min. Cells were then stimulated with IL-1β (Peprotech, cat. No. 211-11B) in 50 µl medium per well at a final concentration of 10 ng/10^6 cells. For unstimulated controls, 50 µl medium only was added. After 24 h, plates were centrifuged (300 *g* for 5 min), and supernatants were transferred to a new plate. Supernatants were stored at −80°C until IL-2-ELISA analysis. Absorbance readings were acquired on a VersaMax Microplate Reader (Molecular Devices) at 450 nm. IL-2 release was assayed on three independent days in triplicate. The obtained results were normalized based on the EL4 WT IL-2 release (Appendix Fig S2C).

Chromium nanopatterned coverslips

Chromium nanopatterned coverslips with the design and specification described (see Fig EV1C–E) were produced by ThunderNIL Srl (Trieste, Italy). Coverslips were fabricated by the pulsed nanoimprint lithography method (Lin *et al*, 2010b) and printed with a master design which contained multiple nanopatterned chromium grids containing square corrals with 2.5 or 1 µm^2 dimensions. Chromium gridlines were 100 nm thick and 5 nm high and were printed on no. 1.5 coverslips with a diameter of 25 mm.

Imaging chambers and supported lipid bilayers

SLBs were prepared using a previously published method (Taylor *et al*, 2017; Deliz-Aguirre *et al*, 2021). Briefly, phospholipid mixtures consisting of 97.5% mol 1-palmitoyl-2-oleoyl-*sn*-glycero-3-phosphocholine (POPC), 2% mol 1,2-dioleoyl-sn-glycero-3-[(N-(5-amino-1-carboxypentyl)iminodiacetic acid)succinyl] (ammonium salt; DGS-NTA), and 0.5% mol 1,2-dioleoyl-sn-glycero-3-phosphoethanolamine-N-[methoxy(polyethylene glycol)-5000] (PE-PEG5000) were mixed in glass round-bottom flasks and dried down with a rotary evaporator. All lipids used were purchased from Avanti Polar Lipids. Dried lipids were placed under vacuum for 2 h to remove trace chloroform and resuspended in PBS. Small unilamellar vesicles (SUVs) were produced by several freeze–thaw cycles combined with bath sonication. Once the suspension had cleared, the SUVs were spun in a benchtop ultracentrifuge at 35,000 *g* for 45 min. SUVs were stored at 4°C for up to a week. IL-1β-functionalized SLBs were formed in 96-well glass bottom plates (Matrical), coverslips (25 mm diameter, No. 1.5 H, Marienfeld-Superior) or on nanopatterned coverslips (25 mm diameter, No. 1.5 H, grid lines produced by ThunderNIL Srl).

To prepare SLBs on 96-well glass bottom plates (Matrical), the plates were cleaned for 30 min with a 5% Hellmanex solution containing 10% isopropanol heated to 50°C, then incubated with 5% Hellmanex solution for 1 h at 50°C, followed by extensive washing with pure water. Ninety-six-well plates were dried with nitrogen gas and sealed until needed. To prepare SLB, individual wells were cut out and base etched for 15 min with 5 M KOH and then washed with PBS. To form SLBs, SUV suspension was deposited in each well or coverslip and allowed to form for 1 h at 45°C. After 1 h, wells were washed extensively with PBS. SLBs were incubated for 15 min with HEPES buffered saline (HBS: 20 mM HEPES, 135 mM NaCl, 4 mM KCl, 10 mM glucose, 1 mM $CaCl_2$, 0.5 mM $MgCl_2$) with 10 mM $NiCl_2$ to charge the DGS-NTA lipid with nickel. The SLBs were then washed in HBS containing 0.1% BSA to block the surface and minimize nonspecific protein adsorption. After blocking, the SLBs were functionalized by incubation for 1 h with His10-IL-1β. The labeling solution was then washed out, and each well was completely filled with HBS with 0.1% BSA. For SLBs set up on 96-well plates, the total well volume was 630 µl (manufacturers specifications), and 530 µl was removed leaving 100 µl of HBS 0.1% BSA in each well. Each SLB was functionalized with 100 µl His10-Halo-IL-1β of twofold desired concentration for 1 h, and excessive ligands were washed away with HBS.

To prepare SLBs on normal or nanopatterned coverslips, the coverslips were cleaned by bath sonication for 30 min in MilliQ H2O. After sonication, coverslips were immersed in freshly prepared piranha solution (sulfuric acid:hydrogen peroxide, 3:1) for 15 min, rinsed in MilliQ water 20 times, and finally dried with nitrogen gas. To form SLBs, we sandwiched 30 µl of a SUV suspension between a petri dish and a coverslip. After a 5-min incubation, the petri dish was immersed in MilliQ water bath. The coverslip was removed from the petri dish and washed in the MilliQ water to remove excessive SUVs. Coverslips were assembled in Attofluor Chamber (Thermo Fisher). The MilliQ water in each chamber was slowly replaced with PBS and incubated with 10 mM $NiCl_2$ for 15 min, followed by incubation with 0.1% BSA for 30 min. Finally, each SLB was functionalized with His10-Halo-IL-1β for 1 h and excessive ligands were washed away with 20 ml HBS.

Protein expression, purification, and labeling

To functionalize the SLBs with active mouse IL-1β, we expressed and purified fusion protein of His10-Halo-IL-1β as previously described (Deliz-Aguirre *et al*, 2021).

© 2023 The Authors

This protein was produced from two separate expression plasmids: pET28a-MmIL1β-Spytag and pET28a-His10-Halo-Tencon-SpycatcherV2. We expressed IL-1β-Spytag and His10-Halo-Tencon-SpycatcherV2 in BL21-DE3 Rosetta *E. coli* (Novagen) grown in Terrific Broth media. After an overnight induction with IPTG, the bacterial culture was pelleted and the cell pellets were resuspended in the lysis buffer (50 mM TRIS pH 8.0, 250 mM NaCl, 5 mM Imidazole with protease inhibitors, Lysozyme 100 μg/ml) and lysed using sonication. To covalently couple His10-Halo-Tencon-Spycatcher to MmIL1β-Spytag, the cleared lysates were mixed and incubated with mild agitation for 1 h at 4°C. To ensure complete Spycatcher-Spytag conjugation, the lysates were mixed with 2:1 ratio (vol:vol, based on starting bacterial culture volume) of MmIL1β-Spytag to His10-Halo-Tencon-Spycatcher. After the conjugation, the His10-Halo-Tencon-Spycatcher-IL-1β-Spytag was purified by Ni-NTA resin. Conjugation was monitored by mobility shift using SDS–PAGE. After elution, the protein was desalted with HiTrap desalting column into 20 mM HEPES and subject to anion exchange chromatography with a MonoQ column. This was followed by gel filtration over Superdex 200 26/600 into storage buffer (20 mM HEPES, 150 mM NaCl). The protein was snap-frozen with the addition of 20% glycerol in liquid nitrogen and placed at −80°C for long-term storage. In text, this protein is referred to as His10-Halo-IL-1β. Following purification, the His10-Halo-Tencon-Spycatcher-IL-1β-Spytag protein was either snap-frozen and stored at −80°C or directly used for HaloTag-labeling. To label the HaloTag, a 2.5× molar excess of JF646-HaloLigand was mixed with the protein and incubated at room temperature for 1 h followed by an overnight incubation at 4°C. Postlabeling, the protein was gel filtered over a Superdex 200 26/600 into storage buffer and snap-frozen with the addition of 20% glycerol in liquid nitrogen and placed in −80°C for storage. The degree of labeling was calculated with a spectrophotometer by comparing 280 nm and 640 nm absorbance (usually 85–95% labeling efficiency was achieved).

For microscopy calibration of mScarlet single-molecule intensity, we used His10-mScarlet-IL-1β (previously described here, Deliz-Aguirre *et al*, 2021). For mEGFP single-molecule intensity, His10-mEGFP was expressed from a pET28a vector and purified with Ni-NTA resin followed by gel filtration. Frozen aliquot of both proteins was stored at -80C.

Immunofluorescence staining and widefield microscopy of RelA nuclear translocation

To analyze the nuclear translocation of RelA in IL-1β-stimulated EL4 cells with or without inhibition of Myddosome coalescence (Fig 1G), IL-1β-functionalized SLBs were prepared on coverslips without chromium grid lines (off grid) or on coverslips with 2.5 or 1 μm grid lines. Nonfunctionalized SLBs served as unstimulated controls. EL4 was incubated for 30 min with IL-1β-labeled SLBs functionalized with 100 IL-1β molecules/μm^2. Cells were then fixed with 3.5% (wt/vol) PFA containing 0.5% (wt/vol) Triton X-100 for 20 min at room temperature. Cells were washed with PBS and blocked with PBS 10% BSA (wt/vol) at 4°C overnight.

The next day, fixed cells were labeled with a one-step staining solution containing anti-RelA (1:400, Cell Signaling Technology, #8242), Nano-secondary AF568 (Chromotek, #srbAF568-1-100, 1:1,000) diluted in PBS 10% (wt/vol) BSA containing 0.1% Triton X-100, for 1 h at room temperature. Cells were labeled with DAPI and 488-phalloidin to label the nucleus and cytosol, respectively. Finally, cells were washed five times in PBS before imaging.

We acquired widefield microscopy images of RelA nuclear translocation on an inverted microscope (Nikon TiE) equipped with Lumencor Spectra-X illumination. Fluorescent images were acquired with Nikon Plan Apo 40× 0.95 NA air objective lens and projected onto a Photometric Prime 95 camera and a 1.5× magnification lens (calculated pixel size of 181.41 nm). Image acquisition was performed with NIS-Elements software.

Immunofluorescence staining of phospho-p65, phospho-IKK, K63-Ub, and M1-Ub

To analyze the colocalization of phospho-IKK, K63-Ub, and M1-Ub with MyD88-GFP (Fig 2), EL4 cells were stimulated with IL-1β-functionalized SLBs for 30 min and then fixed with 3.5% (wt/vol) PFA containing 0.5% (wt/vol) Triton X-100 for 20 min at room temperature. Staining was then performed with a traditional two-step staining method. After fixation, cells were washed with PBS and then blocked in PBS 10% (wt/vol) BSA containing 4% normal goat serum for 1 h at room temperature. Fixed cells were labeled with primary antibodies diluted in PBS 10% (wt/vol) BSA containing 0.1% Triton X-100 at 4 C overnight. The next day, cells were washed five times with PBS and labeled with secondary antibodies (goat anti-rabbit/human conjugated to Alexa Fluor 647; 1:1,000; Invitrogen, #A21246/A21445) and FluoTag-X4 anti-GFP conjugated to Atto488 (1:500, Nano Tag Biotechnology, #N0304-At488-L) for 1 h at room temperature. Finally, cells were washed five times in PBS before imaging with TIRF or SIM microscopy. To analyze the colocalization of phospho-p65 with MyD88, we used a one-step staining protocol detailed below. To image pIKK with SIM (Fig 2C), coverslips were mounted in Prolong Glass Antifade Mountant (Thermo, #P36980).

To quantitatively compare phospho-p65, phospho-IKK, K63-Ub, and M1-Ub staining off and on grids (Figs 3 and 4), EL4-MyD88-GFP cells were starved in serum-free medium for 3–4 h. After starvation, 7 × 10^5 cells were applied to SLBs and incubated at 37°C for 30 min. We used SLB functionalized with 5–25 IL-1β/μm^2. Cells were fixed with a final concentration of 3.5% (wt/vol) PFA containing 0.5% (wt/vol) Triton X-100 for 20 min at room temperature. Cells were washed with PBS and blocked with PBS 10% BSA (wt/vol) at 4°C overnight. The next day, we performed one-step staining. Cells were incubated with an antibody mixture containing primary antibody, Nano-secondary AF568 (Chromotek, #srbAF568-1-100, 1:1,000) prepared in PBS with 10% (wt/vol) BSA and 0.1% Triton X-100. Cells were incubated with this labeling solution at room temperature for 1 h. Across all immunofluorescence experiments, we used the following primary antibodies and labeling concentrations: anti-phospho-p65, 1:800 (Cell Signaling Technology, #3033); anti-phospho-IKK, 1:400 (Cell Signaling Technology, #2697); anti-M1-Ub, 1:2,500 (Matsumoto *et al*, 2012, Genentech); and anti-K63, 1:10,000 (Newton *et al*, 2008, Genentech). Cells were then labeled with FluoTag-X4 anti-GFP conjugated to Atto488 at room temperature for 1 h. After antibody labeling, each chamber was washed with 40 ml PBS. Cells were then imaged immediately with TIRF microscopy.

TIRF microscopy data acquisition

Imaging of MyD88-GFP, mScarlet-TRAF6, and mScarlet-HOIL1 recruitment was performed on an inverted microscope (Nikon TiE) equipped with a Nikon fiber launch TIRF illuminator. Illumination

© 2023 The Authors

was controlled with a laser combiner using the 488-, 561-, and 640-nm laser lines at ~ 0.35, ~ 0.25, and ~ 0.17 mW laser power, respectively (laser power measured after the objective). Fluorescence emission was collected through filters for GFP (525 ± 25 nm), RFP (595 ± 25 nm), and JF646 (700 ± 75 nm). All images were collected using a Nikon Plan Apo 100× 1.4 NA oil immersion objective that projected onto a Photometrics 95B Prime sCMOS camera with 2 x 2 binning (calculated pixel size of 150 nm) and a 1.5× magnifying lens. Image acquisition was performed using NIS-Elements software. All experiments were performed at 37°C. The microscope stage temperature was maintained using an OKO Labs heated microscope enclosure. Images were acquired with an interval of 4 s using exposure times of 60–100 ms.

Imaging EL4 cells endogenously expressing MyD88-GFP, mScarlet-TRAF6, or mScarlet-HOIL1 on IL-1β functionalized SLBs with TIRF microscopy

His10-Halo-JF646-IL-1β-functionalized SLBs were set up as described above. To quantify the density of IL-1β on the SLB, wells were prepared that were functionalized with identical labeling protein concentration and time, but with different molar ratios of labeled to unlabeled His10-Halo-IL-1β. Before application of cells, SLBs were analyzed by TIRF microscopy to check formation, mobility, and uniformity. Short time series were collected at wells containing a ratio of labeled to unlabeled His10-Halo-IL-1β (e.g., < 1 His10-Halo-JF646-IL-1β molecule/μm^2) to calculate ligand densities on the SLB based upon direct single-molecule counting. By controlling the concentration of His10-Halo-JF646-IL-1β in the labeling reaction, we could label SLB with final IL-1β densities ranging from 1 to 200 molecules/μm^2.

Before each imaging experiment, we acquired calibration images using recombinant mEGFP and His10-mScarlet-IL-1β previously described here (Deliz-Aguirre *et al*, 2021). To image a single GFP/mScarlet-i fluorophores, the recombinant purified proteins were diluted in HBS and adsorbed to KOH-cleaned glass. Single molecules of GFP/mScarlet-i were imaged using identical microscope acquisition settings to those used for cellular imaging. To image live cells, EL4 cells were pipetted onto supported lipids bilayers functionalized with His10-Halo-JF646-IL-1β. EL4 cells expressing MyD88-GFP, mScarlet-TRAF6, or mScarlet-HOIL1 were sequentially illuminated for 60–100 ms with 488-nm and 561-nm laser line at a frame interval of 4 s (Fig 5). Diffraction-limited punctate structures of MyD88-GFP, mScarlet-TRAF6, or mScarlet-HOIL1 were detected and tracked using the Fiji TrackMate plugin (Tinevez *et al*, 2017).

Structured illumination microscopy data acquisition

We acquired 3D structured illumination microscopy of fixed EL4 cells on a Zeiss Elyra 7 microscope equipped with 405, 488, 561, and 642 nm laser lines for excitation. Image acquisition was performed with a 63×, NA 1.46 oil objective, and images were captured on pco.edge 4.2 sCMOS camera. We acquired Z stacks of fixed cells using the 3D leap acquisition plugin using a 200 nm Z-axis step size with 13 phases. We performed postprocessing image reconstruction in Zeiss Zen software.

Quantification and statistical analysis

All data are expressed as the mean ± the standard deviation (SD) or mean ± the standard error of the mean (SEM), as stated in the figure legends and results. The exact value of n and what n represents (e.g., number of cells, MyD88-GFP puncta, or experimental replicates) is stated in figure legends and results. Means of experimental replicates were compared using an unpaired two-tailed Student's t-test implemented in R studio. Data distribution was assumed to be normal based on density plots, but this was not formally tested. We performed no blinding of the data for any data analysis performed.

Quantification of immunofluorescence staining of RelA nuclear localization

We quantified widefield microscopy images of RelA nuclear localization in an analysis pipeline implemented in FIJI and Cell Profiler. First, we performed background subtraction from the MyD88-GFP and RelA (Cy3 channel) immunofluorescence staining micrographs in FIJI. Background was removed in two steps: First, we subtracted a dark field image from each image. Second, we estimate cytosolic background by generating median blur from each micrograph. We then subtracted this median blur from the parent micrograph.

We then performed segmentation and quantification using a custom Cell Profiler pipeline that allowed images to be processed in batch. We segmented the cell nucleus using the DAPI channel. Selected nuclei retained for analysis had to have a diameter between 30 and 60 pixels, this excluded small DAPI-stained objects that corresponded to cell fragments and apoptotic cells. Segmentation of the 488-phalloidin staining channel identified the total cell volume. Both segmentation steps were performed using an Otsu threshold. The volume corresponding to cellular cytoplasm is identified by subtracted the total cell volume minus nucleus volume. The RelA staining intensity of the cell nucleus and cytoplasm was extracted, and the ratio calculated. RelA nucleus-to-cytoplasm ratio from images acquired on 2.5 and 1 μm grids and unstimulated negative controls were normalized to the intensity of RelA nucleus-to-cytoplasm ratio from off-grid data. We normalized intensity using the following equation: $Norm.\ Int = (Intensity - quantile(0.05)_{off\ grid})/(quantile(0.95)_{off\ grid} - quantile(0.05)_{off\ grid})$. Finally, we performed data visualization of normalized RelA nucleus-to-cytoplasm ratio in ggplot2 (Fig 1H).

Quantification of immunofluorescence staining and analysis of phospho-p65, phospho-IKK, K63-Ub, and M1-Ub

We quantified TIRF microscopy images of pp65, pIKK, K63-Ub, and M1-Ub immunofluorescence staining in an analysis pipeline implemented in FIJI and Cellprofiler (McQuin *et al*, 2018). First, we performed background subtraction from the MyD88-GFP and immunofluorescence staining TIRF micrographs in FIJI. Background was removed in two steps: First, we subtracted a dark-field image from each image. Second, we estimate cytosolic background by generating median blur from each TIRF micrograph. We then subtracted this median blur from the parent TIRF micrograph.

Next, we segmented MyD88-GFP puncta and quantified fluorescence intensity using a custom Cell Profiler pipeline that allowed images to be processed in batch. We segmented MyD88-GFP puncta using Otsu threshold. Only segmented MyD88-GFP puncta were retained that has a diameter between 3 and 30 pixels. After image segmentation and object detection, the integrated intensity and mean intensity of the MyD88-GFP and immunofluorescence staining channel were extracted for each segmented puncta. We performed

 © 2023 The Authors

manual inspection of the segmented images and objects to verify correct processing and remove incorrectly segmented puncta.

Data normalization and visualization were performed using R. To compare MyD88-GFP puncta size and staining intensity across different replicates acquired on different days, we normalized puncta fluorescence intensities. Fluorescence intensities of MyD88-GFP and immunofluorescence staining from images acquired on 2.5 μm and 1 μm grids were normalized to the intensity of those from off-grid data. We normalized intensity using the following equation: $Norm.\ Int = (Intensity - quantile(0.01)_{off\ grid})/(quantile(0.99)_{off\ grid} - quantile(0.01)_{off\ grid})$.

We used the following criteria to classify MyD88 puncta in fixed cells as single Myddosomes or clusters of Myddosomes (Figs 3G and H, and 4G and H). We observed that MyD88 puncta that formed on grids rarely had a fluorescent intensity greater than 0.5 (normalized integrated intensity, Figs 3E and F, and 4E and F). In contrast, we found that off-grid, between 3 and 5% of MyD88 puncta were classified as clusters (Appendix Fig S1E, F, I and J). This was in agreement with our live-cell measurement of MyD88 puncta size (Fig 1F). Based on these observations and previous measurement that nanopatterned grids disrupted cluster formation (Fig 1F), we defined MyD88 puncta that were clusters as puncta with an intensity ≥ 0.5 and single Myddosomes as puncta being below this threshold. To calculate the per Myddosome staining intensity for clusters (Fig EV2), we divided the MyD88-GFP normalized integrated intensity by this value (0.5), thereby giving an estimate of the number of Myddosomes within a puncta. We then divided the puncta pp65/pIKK/K63-Ub/M1-Ub normalized integrated staining intensity by the number of Myddosomes per puncta, thereby calculating the per Myddosome intensity for each complex within the cluster (Fig EV2). Finally, we performed data visualization of MyD88-GFP puncta size and immunofluorescence staining intensity in ggplot2 and GraphPad Prism (Figs 3A, B and E–H and 4A, B and E–H).

Quantification and analysis of MyD88-GFP puncta and colocalization and recruitment of mScarlet-TRAF6/mScarlet-HOIL1

To quantify the dynamics of MyD88-GFP, mScarlet-TRAF6, and mScarlet-HOIL1, we used an image analysis pipeline described previously (Deliz-Aguirre *et al*, 2021) and briefly described here. First, images in each channel were processed in Fiji to remove background fluorescence. Background subtraction was performed in two steps. First, we subtracted a dark frame image (acquired with no light exposure to the camera, but identical exposure time to experimental acquisition) to remove noise intrinsic to the camera. Then, we subtracted a median-filtered image (generated in Fiji from a median blurred image generated with a radius of 25 pixels) to remove the background associated with cytosolic fluorescence. Next, individual cells were segmented in Fiji according to a maximum projection of MyD88-GFP fluorescence channel. After segmentation, we tracked MyD88-GFP and mSclarlet-TRAF6/mScarlet-HOIL1 puncta in each cell using the Fiji Trackmate plugin (Tinevez *et al*, 2017).

Tracking coordinates generated by Trackmate were imported into MATLAB, and the fluorescence intensity of MyD88-GFP puncta was measured from a 3 × 3 pixel region. To quantify colocalization between MyD88-GFP and mScarlet-TRAF6/HOIL1 puncta, we used the tracking coordinates to identify puncta that colocalized for at least two or more consecutive frames. Colocalized puncta were defined as having centroids ≤ 0.25 μm apart at a given time point. By these criteria, MyD88 tracked puncta were classified as either positive or negative for TRAF6/HOIL1 colocalization.

To estimate the size and number of MyD88s in MyD88-GFP puncta, we acquired images of single mEGFP fluorophores (referred to as simply GFP) absorbed to glass with identical imaging settings to those used in live-cell imaging. Images of single molecules of mEGFP were processed identically to live-cell imaging data, with background subtraction, tracking, and intensity measurement performed as described above. Once intensity measurements were obtained for single molecules of GFP, this was used to divide the fluorescence intensity of MyD88-GFP puncta to yield an estimate of MyD88 copy number. To normalize the puncta by the number of Myddosome complexes, we divided GFP normalized intensity by 4.5 (e.g., the intensity of a single Myddosome, based on the broad fluorescent intensity distribution of a complex containing 6× MyD88-GFP (Lin *et al*, 2010a), see Fig EV3B, and (Deliz-Aguirre *et al*, 2021)). Using these criteria, a MyD88 puncta is defined as a Myddosome complex if the fluorescent intensity is greater than or equal to 4.5× GFP and is a cluster of Myddosomes (e.g., 2 or more complex) if the fluorescent intensity is greater than or equal to 9× mean intensity of GFP.

Finally, we performed data analysis and visualization in R. MyD88-GFP puncta with an intensity ≥ 4.5× the mean intensity of mEGFP were defined as fully assembled Myddosome complexes (see Deliz-Aguirre *et al*, 2021). Myddosome clusters (defined as MyD88-GFP puncta containing two or more Myddosome complexes) were defined as MyD88-GFP puncta with an intensity ≥ 9× the mean intensity of mEGFP (see Fig EV3B).

Quantification of TRAF6/HOIL1 recruitment percentage and lifetime as a function of Myddosome cluster size

To categorize Myddosome clusters in different bins, the max number of Myddosome clusters was rounded to integers and was binned into single Myddosomes, then by every three Myddosomes (e.g., 2–4, 5–7, 8–10, and so on Myddosomes per puncta). The percentage of TRAF6/HOIL1-positive MyD88 at increasing cluster sizes was plotted as a dot plot (Fig 5E). The percentage of TRAF6-positive MyD88 puncta for single Myddosomes and increasing bin sizes of Myddosome clusters are 8.7 ± 2.0% (single Myddosomes), 47.7 ± 7.7% (2–4× Myddosomes per puncta), 77.8 ± 5.0% (5–7× Myddosomes per puncta), 88.7 ± 3.9% (8–10× Myddosomes per puncta), 88.9 ± 3.7% (11–13× Myddosomes per puncta), and 97.0 ± 3.0% (14–16× Myddosomes per puncta). The percentage of HOIL1-positive MyD88 are 0.9 ± 0.4% (single Myddosomes), 12.4 ± 4.3% (2–4× Myddosomes per puncta), 34.3 ± 8.6% (5–7× Myddosomes per puncta), 43.4 ± 8.4% (8–10× Myddosomes per puncta), 55.0 ± 8.2% (11–13× Myddosomes per puncta), and 62.4 ± 7.8% (14–16× Myddosomes per puncta). The lifetime of TRAF6 and HOIL1 puncta that colocalized with those specific Myddosome clusters was plotted as histograms (Fig 7A). We measured 2,079 and 3,170 lifetimes for TRAF6 recruitment to single Myddosomes, 1,427 and 713 lifetimes at clusters composed of 2–4 Myddosomes, 91 and 26 lifetimes at clusters composed of 5–7 Myddosomes, off and on grids, respectively (7.6 and 4.6% of total single Myddosomes, 21.2 and 25.7% of total 2–4 Myddosomes, and 28.8 and 38.8% of total 5–7 Myddosomes off and on grids at 10 IL-1/μm^2). We measured 475 and 668 lifetimes of HOIL1 recruitment to single Myddosomes,

2,051 and 1,171 lifetimes at clusters composed of 2–4 Myddosomes, and 1,130 and 275 lifetimes at clusters composed of 5–7 Myddosomes, off and on grids, respectively (0.7 and 1.1% of total single Myddosomes, 6.4 and 13.3% of total 2–4 Myddosomes, and 11.5 and 40.4% of total 5–7 Myddosomes off and on grids at 32 IL-1/μm^2).

Data availability

The image analysis scripts: GitHub (https://github.com/MJ-Taylor-Lab/Analysis-for-Ub-paper). In an original preprint version of this paper, imaging datasets were deposited here: Zendo Data Depository 10.5281/zenodo.7408704, https://zenodo.org/record/7408704).

Expanded View for this article is available online.

Acknowledgements
We thank Luke Lavis (Janelia Research Campus, Ashburn, VA) for providing Janelia Fluor dyes. We acknowledge Olivia Majer (MPIIB) for access and assistance with 3D structured illumination microscopy and for critical reading of the manuscript. We thank Mark Cronan for reading and commenting on an early version of this manuscript. We thank Vishva Dixit and Genentech for providing specific antibodies against K63/M1-Ub. We thank Paulina Dirvanskyte for helpful suggestions in the writing of this paper. We thank Enfu Hui (UCSD) for critical feedback on the manuscript. We thank all Taylor lab members for critical feedback and commenting on the project. This work was supported by the Max Planck Society. Open Access funding enabled and organized by Projekt DEAL.

Author contributions
Fakun Cao: Conceptualization; resources; data curation; software; formal analysis; validation; investigation; visualization; methodology; writing – original draft; writing – review and editing. **Rafael Deliz-Aguirre:** Software; formal analysis; visualization; methodology. **Fenja HU Gerpott:** Data curation; investigation. **Elke Ziska:** Data curation; investigation. **Marcus J Taylor:** Conceptualization; data curation; supervision; funding acquisition; investigation; visualization; methodology; writing – original draft; project administration; writing – review and editing.

Disclosure and competing interests statement
The authors declare that they have no conflict of interest.

References

Bindels DS, Haarbosch L, van Weeren L, Postma M, Wiese KE, Mastop M, Aumonier S, Gotthard G, Royant A, Hink MA *et al* (2017) mScarlet: a bright monomeric red fluorescent protein for cellular imaging. *Nat Methods* 14: 53–56

Case LB, Ditlev JA, Rosen MK (2019) Regulation of transmembrane signaling by phase separation. *Annu Rev Biophys* 48: 465–494

Cheng QJ, Ohta S, Sheu KM, Spreafico R, Adelaja A, Taylor B, Hoffmann A (2021) NF-κB dynamics determine the stimulus specificity of epigenomic reprogramming in macrophages. *Science* 372: 1349–1353

DeFelice MM, Clark HR, Hughey JJ, Maayan I, Kudo T, Gutschow MV, Covert MW, Regot S (2019) NF-κB signaling dynamics is controlled by a dose-sensing autoregulatory loop. *Sci Signal* 12: eaau3568

Deliz-Aguirre R, Cao F, Gerpott FHU, Auevechanichkul N, Chupanova M, Mun Y, Ziska E, Taylor MJ (2021) MyD88 oligomer size functions as a physyical threshold to trigger IL1R Myddosome signaling. *J Cell Biol* 220: e202012071

Ditlev JA, Case LB, Rosen MK (2018) Who's in and who's out-compositional control of biomolecular condensates. *J Mol Biol* 430: 4666–4684

Du M, Ea C-K, Fang Y, Chen ZJ (2022) Liquid phase separation of NEMO induced by polyubiquitin chains activates NF-κB. *Mol Cell* 82: 2415–2426.e5

Emmerich CH, Ordureau A, Strickson S, Arthur JSC, Pedrioli PGA, Komander D, Cohen P (2013) Activation of the canonical IKK complex by K63/M1-linked hybrid ubiquitin chains. *Proc Natl Acad Sci USA* 110: 15247–15252

Hughey JJ, Gutschow MV, Bajar BT, Covert MW (2015) Single-cell variation leads to population invariance in NF-κB signaling dynamics. *Mol Biol Cell* 26: 583–590

Iwai K (2012) Diverse ubiquitin signaling in NF-κB activation. *Trends Cell Biol* 22: 355–364

Jaqaman K, Ditlev JA (2021) Biomolecular condensates in membrane receptor signaling. *Curr Opin Cell Biol* 69: 48–54

Latty SL, Sakai J, Hopkins L, Verstak B, Paramo T, Berglund NA, Cammorota E, Cicuta P, Gay NJ, Bond PJ *et al* (2018) Activation of Toll-like receptors nucleates assembly of the MyDDosome signaling hub. *Elife* 7: e31377

Latz E, Visintin A, Lien E, Fitzgerald KA, Monks BG, Kurt-Jones EA, Golenbock DT, Espevik T (2002) Lipopolysaccharide rapidly traffics to and from the Golgi apparatus with the toll-like receptor 4-MD-2-CD14 complex in a process that is distinct from the initiation of signal transduction. *J Biol Chem* 277: 47834–47843

Lin S-C, Lo Y-C, Wu H (2010a) Helical assembly in the MyD88-IRAK4-IRAK2 complex in TLR/IL-1R signalling. *Nature* 465: 885–890

Lin W-C, Yu C-H, Triffo S, Groves JT (2010b) Supported membrane formation, characterization, functionalization, and patterning for application in biological science and technology. *Curr Protoc Chem Biol* 2: 235–269

Magupalli VG, Negro R, Tian Y, Hauenstein AV, Di Caprio G, Skillern W, Deng Q, Orning P, Alam HB, Maliga Z *et al* (2020) HDAC6 mediates an aggresome-like mechanism for NLRP3 and pyrin inflammasome activation. *Science* 369: eaas8995

Matsumoto ML, Dong KC, Yu C, Phu L, Gao X, Hannoush RN, Hymowitz SG, Kirkpatrick DS, Dixit VM, Kelley RF (2012) Engineering and structural characterization of a linear polyubiquitin-specific antibody. *J Mol Biol* 418: 134–144

McQuin C, Goodman A, Chernyshev V, Kamentsky L, Cimini BA, Karhohs KW, Doan M, Ding L, Rafelski SM, Thirstrup D *et al* (2018) CellProfiler 3.0: Next-generation image processing for biology. *PLoS Biol* 16: e2005970

Motshwene PG, Moncrieffe MC, Grossmann JG, Kao C, Ayaluru M, Sandercock AM, Robinson CV, Latz E, Gay NJ (2009) An oligomeric signaling platform formed by the Toll-like receptor signal transducers MyD88 and Irak-4. *J Biol Chem* 284: 25404–25411

Newton K, Matsumoto ML, Wertz IE, Kirkpatrick DS, Lill JR, Tan J, Dugger D, Gordon N, Sidhu SS, Fellouse FA *et al* (2008) Ubiquitin chain editing revealed by polyubiquitin linkage-specific antibodies. *Cell* 134: 668–678

Nimma S, Ve T, Williams SJ, Kobe B (2017) Towards the structure of the TIR-domain signalosome. *Curr Opin Struct Biol* 43: 122–130

Nunns H, Goentoro L (2018) Signaling pathways as linear transmitters. *Elife* 7: e33617

Shah NA, Sarkar CA (2011) Robust network topologies for generating switch-like cellular responses. *PLoS Comput Biol* 7: e1002085

 © 2023 The Authors

Son M, Wang AG, Tu H-L, Metzig MO, Patel P, Husain K, Lin J, Murugan A, Hoffmann A, Tay S (2021) NF-κB responds to absolute differences in cytokine concentrations. *Sci Signal* 14: eaaz4382

Szymczak-Workman AL, Vignali KM, Vignali DAA (2012) Design and construction of 2A peptide-linked multicistronic vectors. *Cold Spring Harb Protoc* 2012: 199 – 204

Tarantino N, Tinevez J-Y, Crowell EF, Boisson B, Henriques R, Mhlanga M, Agou F, Israël A, Laplantine E (2014) TNF and IL-1 exhibit distinct ubiquitin requirements for inducing NEMO-IKK supramolecular structures. *J Cell Biol* 204: 231 – 245

Tay S, Hughey JJ, Lee TK, Lipniacki T, Quake SR, Covert MW (2010) Single-cell NF-kappaB dynamics reveal digital activation and analogue information processing. *Nature* 466: 267 – 271

Taylor MJ, Husain K, Gartner ZJ, Mayor S, Vale RD (2017) A DNA-based T cell receptor reveals a role for receptor clustering in ligand discrimination. *Cell* 169: 108 – 119.e20

Tinevez J-Y, Perry N, Schindelin J, Hoopes GM, Reynolds GD, Laplantine E, Bednarek SY, Shorte SL, Eliceiri KW (2017) TrackMate: an open and extensible platform for single-particle tracking. *Methods* 115: 80 – 90

Tokunaga F, Sakata S-I, Saeki Y, Satomi Y, Kirisako T, Kamei K, Nakagawa T, Kato M, Murata S, Yamaoka S *et al* (2009) Involvement of linear polyubiquitylation of NEMO in NF-kappaB activation. *Nat Cell Biol* 11: 123 – 132

Wertz IE, Dixit VM (2010) Signaling to NF-kappaB: regulation by ubiquitination. *Cold Spring Harb Perspect Biol* 2: a003350

Wu H (2013) Higher-order assemblies in a new paradigm of signal transduction. *Cell* 153: 287 – 292

Ye H, Arron JR, Lamothe B, Cirilli M, Kobayashi T, Shevde NK, Segal D, Dzivenu OK, Vologodskaia M, Yim M *et al* (2002) Distinct molecular mechanism for initiating TRAF6 signalling. *Nature* 418: 443 – 447

Yin Q, Lin S-C, Lamothe B, Lu M, Lo Y-C, Hura G, Zheng L, Rich RL, Campos AD, Myszka DG *et al* (2009) E2 interaction and dimerization in the crystal structure of TRAF6. *Nat Struct Mol Biol* 16: 658 – 666

Yu X, Zhang L, Shen J, Zhai Y, Jiang Q, Yi M, Deng X, Ruan Z, Fang R, Chen Z *et al* (2021) The STING phase-separator suppresses innate immune signalling. *Nat Cell Biol* 23: 330 – 340

License: This is an open access article under the terms of the Creative Commons Attribution License, which permits use, distribution and reproduction in any medium, provided the original work is properly cited.

Expanded View Figures

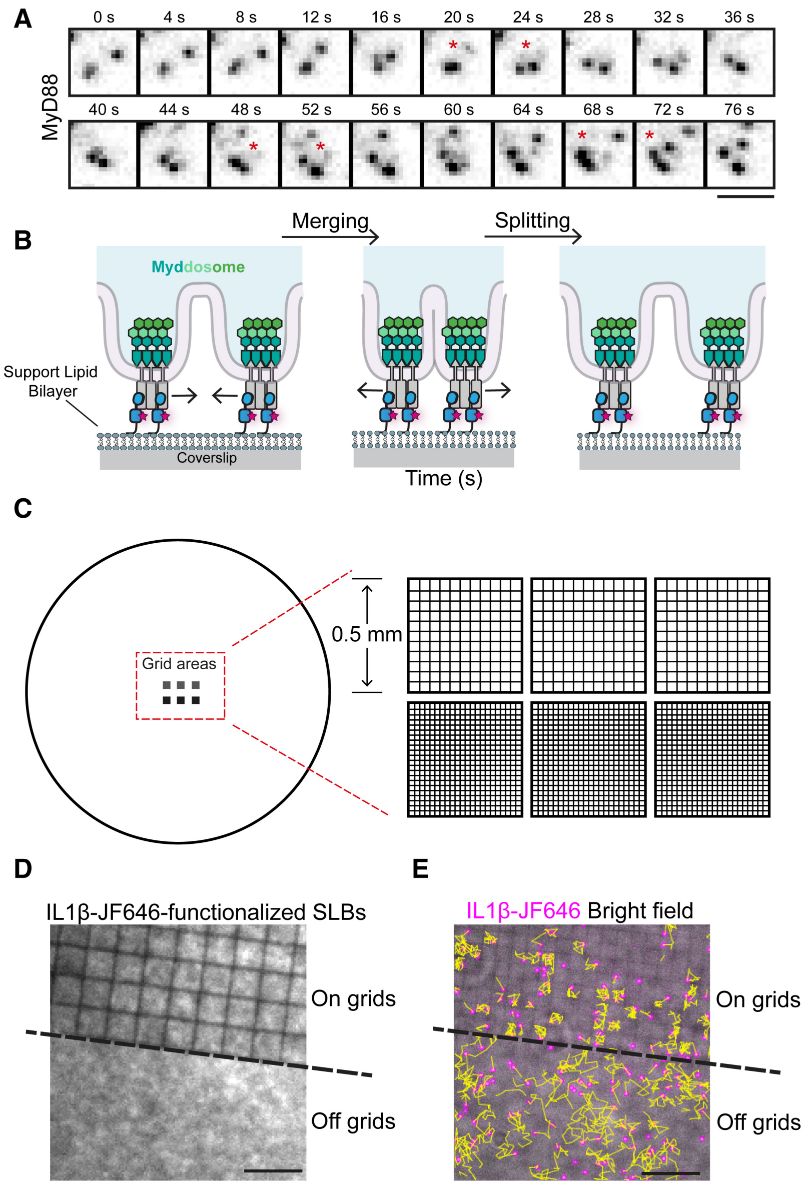

Figure EV1. Illustrations of nanopatterned coverslips to assemble partitioned SLBs.

A Montage of TIRF images showing examples of Myddosomes merging to form clusters and Myddosomes splitting into small puncta. Asterisks indicate frames of merging and splitting. Scale bar, 2 μm.

B Schematic of an alternative membrane topology of Myddosome clusters than the membrane topology presented in Fig 1B.

C Schematic of the coverslip used for preparing partitioned SLBs. The no. 1.5 coverslips with a diameter of 25 mm were fabricated with nanopatterned chromium grids containing square corrals with 2.5 or 1 μm^2 dimensions. Each dimension contains three pieces of square grids with a side length of 0.5 mm.

D TIRF image showing IL-1-JF646 functionalized SLBs at a field of view containing on grids and off 2.5 μm grids SLBs. Continuous SLBs are observed off grids and on each square corral on grids. Scale bar, 5 μm.

E Mobility of IL-1 ligands off and on 2.5 μm grids. Yellow lines are trajectories of IL-1-JF646. Off grids, IL-1 can freely move around, while on grids, IL-1 can only move within individual corrals. Scale bar, 10 μm.

© 2023 The Authors

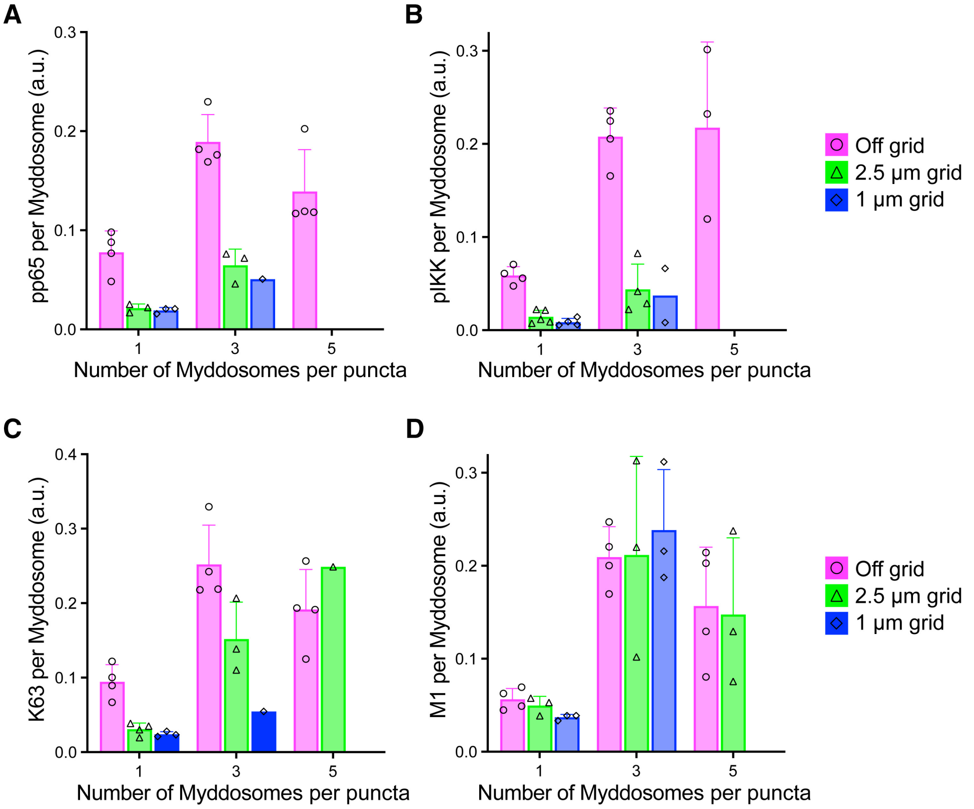

Figure EV2. Staining intensity per complex of single Myddosome and Myddosomes organized into clusters.

A–D The staining intensity for pp65, pIKK, K63-Ub, and M1-Ub (A–D, respectively) of MyD88-GFP puncta using the same datasets from Figs 3 and 4. The staining intensity is normalized to the number of Myddosome contained within the puncta. Data points represent averages from individual biological replicates (three biological replicates for off-grid cells stained for pp65, four biological replicates for off-grid cells stained for pIKK, K63-Ub, and M1-Ub, three biological replicates for 2.5 and 1 µm grids stained for pp65, pIKK, K63-Ub, and M1-Ub). Bars represent mean ± SEM.

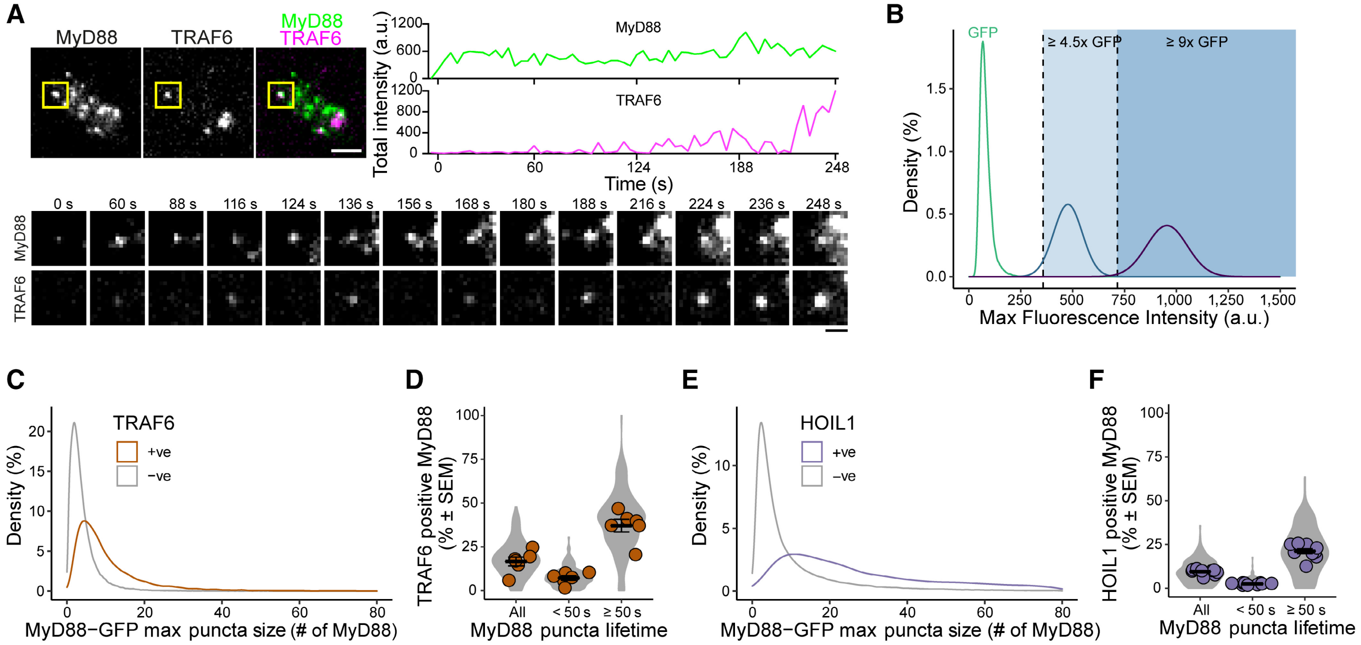

Figure EV3. Characterization of dynamics of MyD88-GFP/mScarlet-TRAF6 and MyD88-GFP/mScarlet-HOIL1 cells.

A TIRF images of an EL4 cell expressing MyD88-GFP and mScarlet-TRAF6. Scale bar, 2 μm. Time-series images indicate MyD88 and TRAF6 puncta from the yellow boxed area. Scale bar in time-series images, 1 μm. TRAF6 is transiently recruited to MyD88 until TRAF6 becomes stable and nucleates on MyD88 puncta. Fluorescence intensities of MyD88 and TRAF6 overtime are shown at top right.

B Density plot of single molecules of GFP (green, n = 40,229 GFP particles) and estimated intensity distribution of a 6× GFP multimer (blue) and a 12× GFP multimer (purple). Shaded light blue and dark blue regions designate intensity values ≥ 4.5× GFP and ≥ 9× GFP, respectively, which were used to categorize MyD88 puncta as containing ≥ 1 or ≥ 2 Myddosome complexes.

C Density plot showing the distribution of MyD88 oligomer size (number of MyD88-GFP monomers is derived from the maximum intensity divided by the average intensity of GFP) for MyD88 puncta that are positive (+ve) or negative (−ve) for TRAF6. The average size for puncta positive or negative for TRAF6 recruitment is 10.4 versus 3.9 MyD88s, measured from 13,526 positive MyD88 puncta versus 83,462 negative MyD88 puncta measured in 191 cells and combined from six biological replicates.

D Quantification of the percentage of MyD88-GFP puncta per cell that colocalizes with TRAF6 for all puncta and puncta with lifetimes < 50 s or ≥ 50 s. Violin plots show the distribution of individual cell measurements. Colored dots superimposed on violin plots correspond to the average value in the independent experiments (n = 6 biological replicates, with 17–48 cells measured per replicate). Bars represent mean ± SEM.

E Density plot showing the distribution of MyD88 oligomer size (number of MyD88-GFP monomers is derived from the maximum intensity divided by the average intensity of GFP) for MyD88 puncta that are positive (+ve) or negative (−ve) for HOIL1. The average size for puncta positive or negative for HOIL1 recruitment is 47.4 versus 11.6 MyD88s, mean calculated from 10,300 HOIL1-positive MyD88 puncta versus 108,054 negative MyD88 puncta measured from 230 cells and combined across nine biological replicates.

F Quantification of the percentage of MyD88-GFP puncta per cell that colocalizes with HOIL1 for all puncta and puncta with lifetimes < 50 s or ≥ 50 s. Violin plots show the distribution of individual cell measurements. Colored dots superimposed on violin plots correspond to the average value in the independent experiments (n = 9 biological replicates, with 9–46 cells measured per replicate). Bars represent mean ± SEM.

 © 2023 The Authors

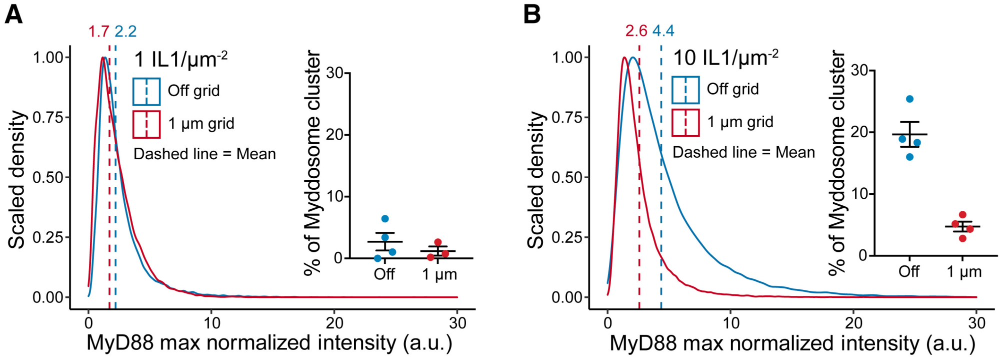

Figure EV4. Characterization of dynamics of MyD88-GFP puncta size in MyD88-GFP/mScarlet-TRAF6 cells off grids and on 1 µm grids.

A, B Scaled density distribution of MyD88 max normalized intensity off grids and on 1 µm grids at a ligand density of 1 (A) or 10 (B) IL1/µm^2. The average MyD88 max normalized intensity (dashed line) at 1 IL1/µm^2 off grids versus on 1 µm grids are 2.2 versus 1.7, and at 10 IL1/µm^2 are 4.4 versus 2.6. Insets are quantifications of the percentages of Myddosome clusters. A Myddosome cluster is defined as a MyD88-GFP puncta containing equal to or greater than two Myddosomes. At 1 IL-1/µm^2, the percentages of Myddosome clusters off grids versus on 1 µm grids are 2.7 ± 1.4% versus 1.2 ± 0.7% and at 10 IL1/µm^2 are 19.7 ± 2.0% versus 4.7 ± 0.8%. Bars represent mean ± SEM. At 1 IL1/µm^2, data are measured from 24,315 MyD88 puncta off grids from 91 cells and four replicates, and 23,161 MyD88 puncta on 1 µm grids from 70 cells and three replicates. At 10 IL1/µm^2, data are measured from 34,452 MyD88 puncta off grids from 87 cells and four biological replicates, and 71,525 MyD88 puncta on 1 µm grids from 100 cells and four biological replicates.

© 2023 The Authors

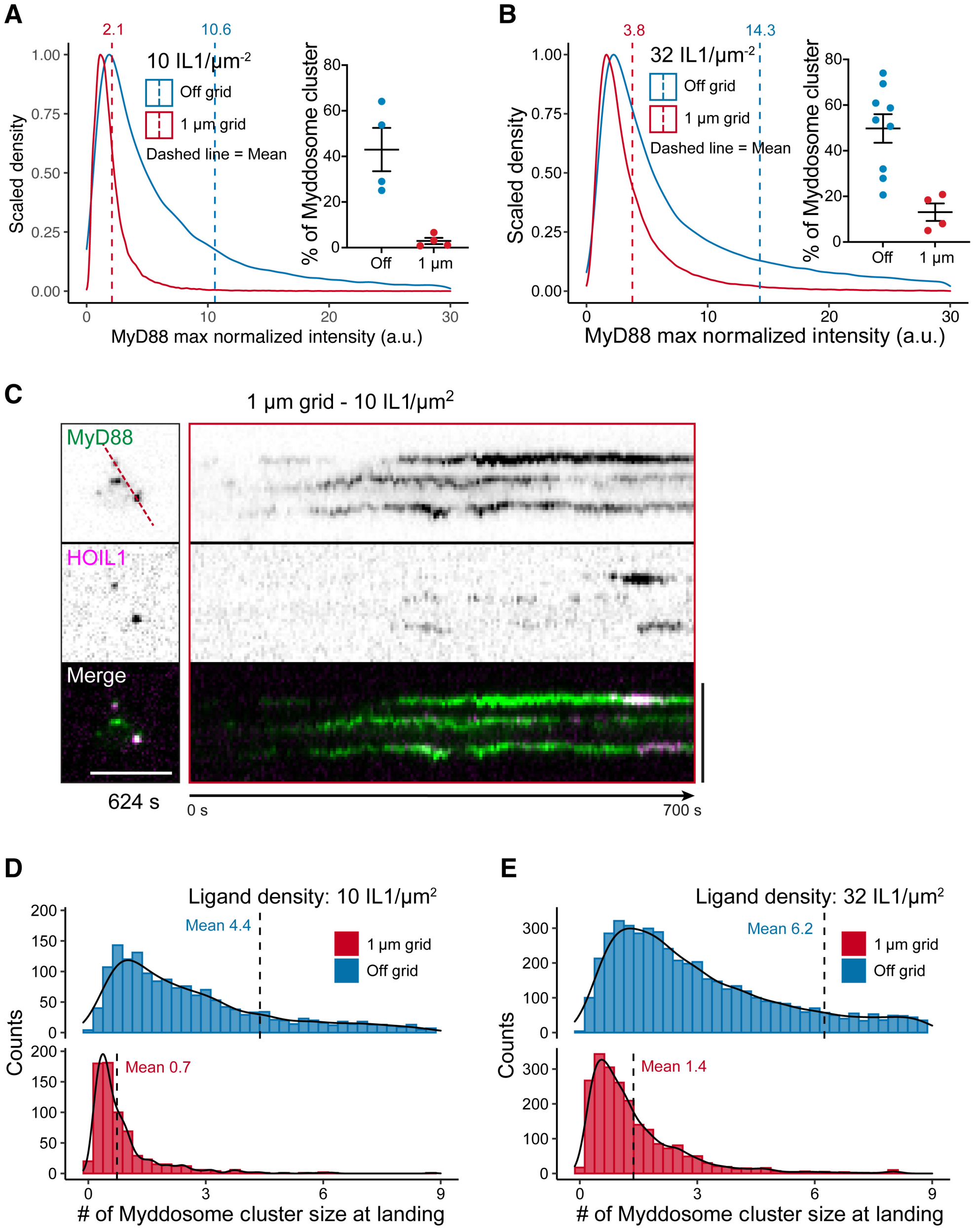

Figure EV5. Characterization of dynamics of MyD88-GFP/mScarlet-HOIL1 cells off and on 1 μm grids.

A, B Scaled density distribution of MyD88 max normalized intensity off grids and on 1 μm grids at a ligand density of 10 (A) or 32 (B) IL1/μm^2. The average MyD88 max normalized intensity (dashed line) at 10 IL1/μm^2 off grids versus on 1 μm grids is 10.6 versus 2.1 and at 32 IL1/μm^2 is 14.3 versus 3.8. Insets are quantifications of the percentage of Myddosome clusters. A Myddosome cluster is defined as a MyD88-GFP puncta containing equal to or greater than 2 Myddosomes. At 10 IL1/μm^2, the percentages of Myddosome clusters off grids versus on 1 μm grids are 43.0 ± 9.5% versus 2.9 ± 1.3%, and at 32 IL1/μm^2 are 49.8 ± 6.3% versus 13.1 ± 3.9%. Bars represent mean ± SEM. At 10 IL1/μm^2, data are measured from 53,852 MyD88 puncta off grids from 74 cells and four biological replicates and 55,075 MyD88 puncta on 1 μm grids from 154 cells and four biological replicates. At 32 IL1/μm^2, data are measured from 118,354 MyD88 puncta off grids from 230 cells and nine biological replicates and 68,819 MyD88 puncta on 1 μm grids from 138 cells and four biological replicates.

C An example of TIRF images of HOIL1 recruitment in EL4 cells expressing MyD88-GFP and mScarlet-HOIL1 stimulated on IL1 functionalized SLBs on 1 μm grids at a ligand density of 10 IL1/μm^2. Kymographs derived from dashed lines overlaid TIRF images (left panel). Scale bar, 5 μm.

D, E Histogram of the average landing size for HOIL1 at 10 IL1/μm^2 (H) and 32 IL1/μm^2 (I) off and on 1 μm grids, overlaid with density plots of the distribution. The landing size of Myddosome is calculated with landing size of MyD88 puncta divided by the intensity of 4.5× GFP. The average landing size of Myddosome at 10 IL1/μm^2 off grids versus on 1 μm grids is 4.4 ± 1.2 versus 0.7 ± 0.2 Myddosomes (Mean ± SEM), measured from 1762 versus 691 MyD88-GFP puncta from 66 versus 86 cells and 4 versus 4 replicates. The average landing size of Myddosome at 32 IL1/μm^2 off grids versus on 1 μm grids is 6.2 ± 1.1 versus 1.4 ± 0.2 Myddosomes (Mean ± SEM), measured from 5,562 versus 2,189 MyD88-GFP puncta from 212 versus 124 cells and 9 versus 4 biological replicates.

 © 2023 The Authors

Appendix for **Myddosome clustering in IL-1 receptor signaling regulates the formation of an NF-kB activating signalosome**

Fakun Cao, Rafael Deliz-Aguirre, Fenja H. U. Gerpott, Elke Ziska, and Marcus J. Taylor*

Max Planck Institute for Infection Biology, Chariteplatz 1, Berlin D-10117 Germany

* Corresponding author: taylor@mpiib-berlin.mpg.de

Table of Contents:

Appendix Figure S1: page 2

Appendix Figure S2: page 4

Appendix Figure S3: page 6

Appendix Figure S4: page 8

Appendix Figure S5: page 9

Appendix Figure S1

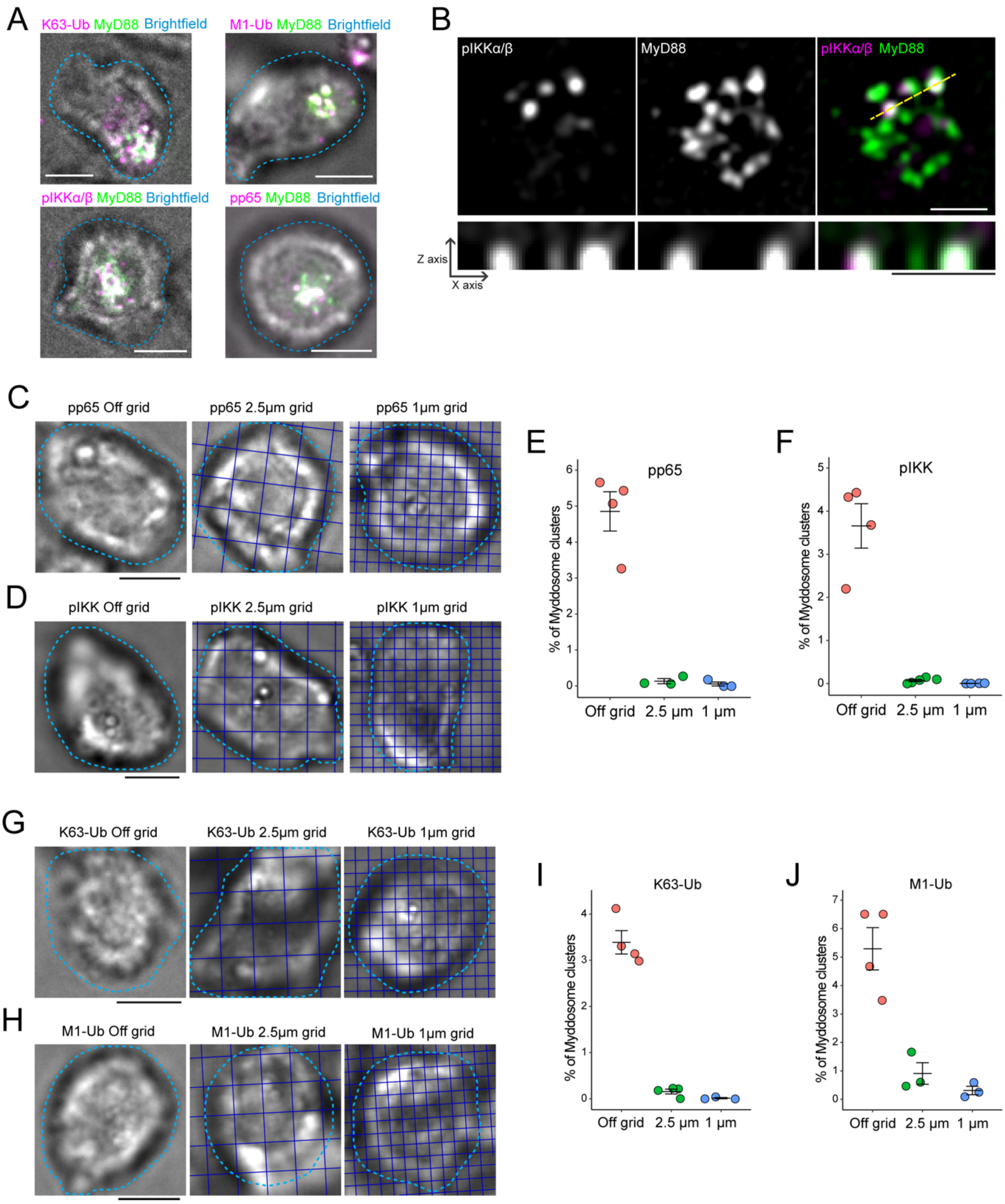

Appendix Figure S1: Additional immunofluorescence images and analysis

A. Brightfield images from Fig. 2A. Brightfield images overlaid with cell contour (blue dashed line) and overlaid with MyD88-GFP and antibody staining. Scale bar, 5 μm.

B. Additional SIM images showing colocalization between Myddosome and pIKKa/b. Scale bar, 1 μm.

(C and D) Brightfield images for Fig. 3A and 3C (A), and Fig. 3B and 3D (B). Cyan dashed lines are cell contour and blue lines indicate grid lines. Scale bar, 5 μm.

(E and F) Quantification of the percentage of MyD88 puncta classified as Myddosome clusters in pp65 (E) or pIKK (F) immunofluorescence staining off grids, on 2.5 μm and on 1

µm grids. MyD88 puncta with an integrated intensity greater than or equal to 0.5 are classified as Myddosome clusters. The percentage of Myddosome clusters off grids, on 2.5 µm and on 1 µm grids in pp65 staining are 4.853 ± 0.547%, 0.133 ± 0.072% and 0.055 ± 0.055%, respectively; and that in pIKK staining are 3.657 ± 0.515%, 0.071 ± 0.026% and 0.004 ± 0.003%, respectively. Bars represent mean ± SEM (n = 3 - 4 biological replicates for pp65, with 259 - 9342 puncta measured per replicate, and 10273, 14009 and 2675 puncta measured off grids, on 2.5 µm and 1 µm grids, respectively; n = 4 - 5 biological replicates for pIKK, with 435 - 24998 puncta measured per replicate, and 2375, 35496 and 59593 puncta measured off grids, on 2.5 µm and 1 µm grids, respectively)

(G and H) Brightfield images for Fig. 4A and 4C (A), and Fig. 4B and 4D (B). Cyan dashed lines are cell contour and blue lines indicate grid lines. Scale bars, 5 µm.

(I and J) Quantification of the percentage of MyD88 puncta classified as Myddosome clusters in K63-Ub (C) or M1-Ub (D) immunofluorescence staining off grids, on 2.5 µm and on 1 µm grids. MyD88 assemblies with an integrated intensity greater than or equal to 0.5 are classified as large MyD88 assemblies. The percentage of large MyD88 assemblies off grids, on 2.5 µm and on 1 µm grids in K63-Ub staining are 3.387 ± 0.253%, 0.156 ± 0.053% and 0.013 ± 0.013%, respectively; and that in M1-Ub staining are 5.292 ± 0.744%, 0.908 ± 0.378% and 0.310 ± 0.147%, respectively. Bars represent mean ± SEM (n = 3 - 4 biological replicates for K63-Ub, with 484 - 14628 puncta measured per replicate, and 14571, 27494 and 24026 puncta measured off grids, on 2.5 µm and 1 µm grids, respectively; n = 3 - 4 biological replicates for M1-Ub, with 338 - 3887 puncta measured per replicate, and 3114, 6091 and 1844 puncta measured off grids, on 2.5 µm and 1 µm grids, respectively).

Appendix Figure S2

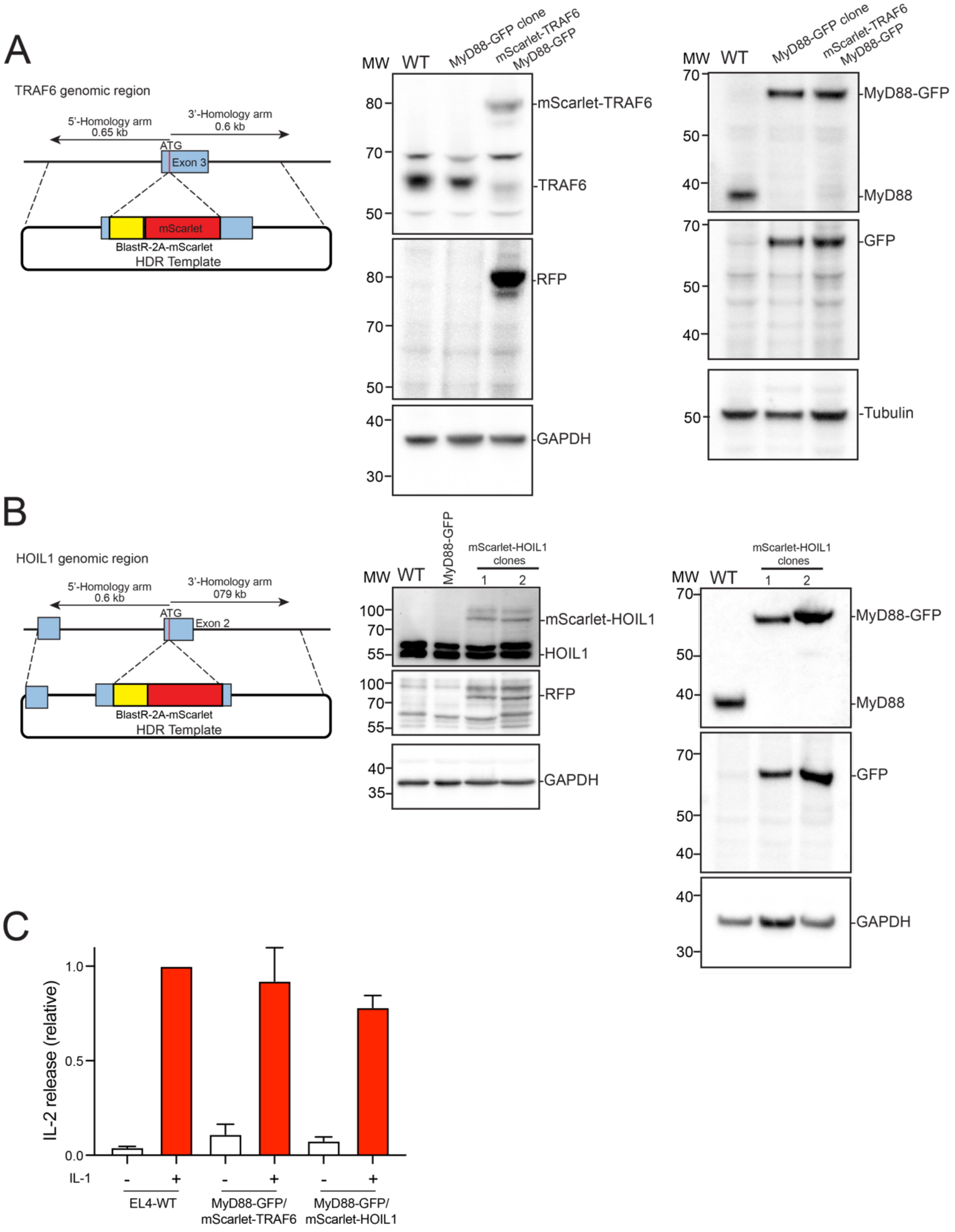

Appendix Figure S2. Validation of EL4 cell lines with CRISPR/Cas9 gene edited MyD88-GFP and mScarlet-TRAF6/HOIL1.

(A and B) Left, schematic of the TRAF6 (A) or HOIL1 (B) gene locus and HDR template designed to insert a mScarlet open reading frame immediately after the start codon. EL4 cells were electroporated with HDR and gRNA/Cas9 plasmids to simultaneously edit MyD88 and TRAF6 (A) or MyD88 and HOIL1 (B) gene loci. Middle, western blot analysis of mScarlet-TRAF6 (A) or HOIL1 (B) in dual gene-edited EL4 clones. Western blots of whole cell

lysates were probed with anti-TRAF6/HOIL1 and anti-RFP antibodies to confirm editing and insertion of fluorescent protein open reading frames at both gene loci. WT EL4 cells and EL4 cells expressing MyD88-GFP only served as negative controls. Right, western blot analysis of MyD88-GFP in dual gene-edited EL4 clones. Western blots of same lysates were probed with anti-MyD88 and anti-GFP to confirm editing and insertion of GFP open reading frame at the MyD88 gene locus. WT EL4 cells served as a negative control and EL4 cells expressing MyD88-GFP only served as a positive control. For MyD88-GFP/mScarlet-HOIL1 cells, all data presented were acquired with clone 1.
(C) IL-2 release in WT and dual gene-edited EL4 cells. IL-2 release was measured by ELISA 24 h after IL-1β stimulation. Values for gene-edited cells shown relative to EL4 WT. Average values are calculated from 3-4 independent experiments. Bars represent mean ± SEM.

Appendix Figure S3

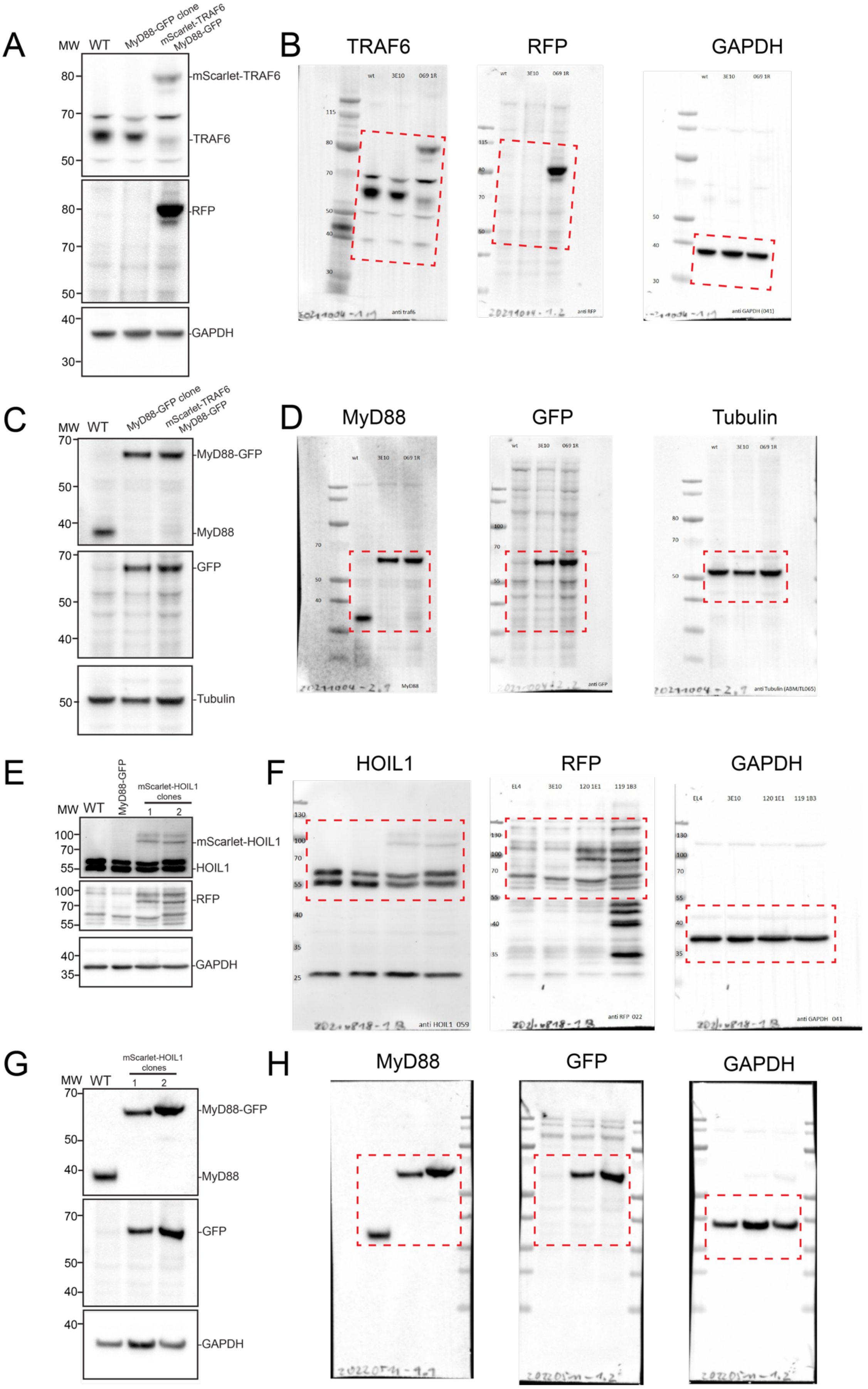

Appendix Figure S3. Full western blots.
A) Cropped blots from Appendix Figure S2A (middle panel)
B). Full western blots of MyD88-GFP/mScarlet-TRAF6 cell line for Appendix Figure S2A (middle panel).
C) The cropped blot from Appendix Figure S2A (right panel)
D) Full western blots of MyD88-GFP/mScarlet-TRAF6 cell line for Appendix Figure S2A (right panel).
E). Cropped blots from Appendix Figure S2B (middle panel)
F) Full western blots of MyD88-GFP/mScarlet-HOIL1 cell line for Appendix S2B (middle panel).
G) Cropped blots from Appendix Figure S2B (right panel)
H) Full western blots of MyD88-GFP/mScarlet-HOIL1 cell line for Appendix S2B (right panel).
All cropped blots are the same as those shown in Appendix Figure S2. Cropped area indicated with red box overlaid the full length blots.

Appendix Figure S4

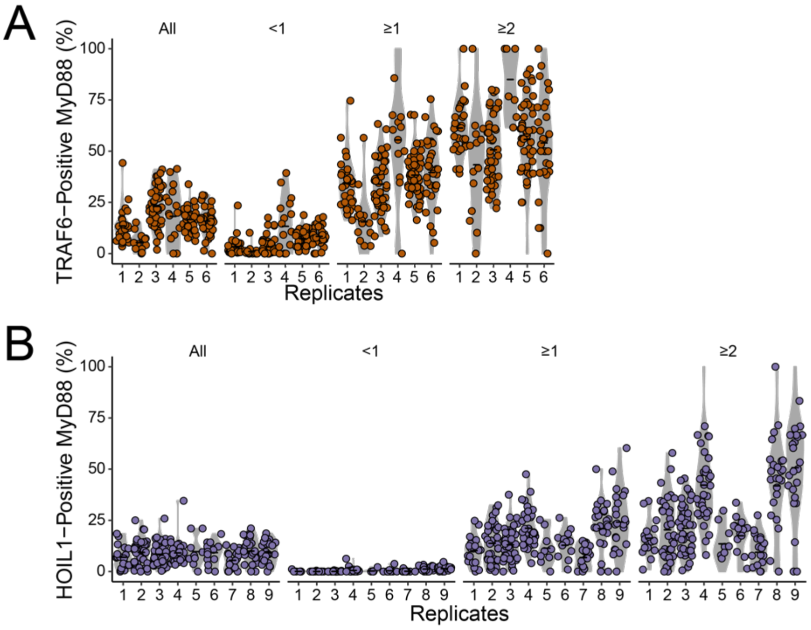

Appendix Figure S4. Individual replicates of mScarlet-TRAF6 and mScarlet-HOIL1 recruitment to single Myddosomes and Myddosome clusters.

A. Percentage (%) of MyD88-GFP puncta that colocalizes with TRAF6 per cell for all puncta, and puncta categorized as containing <1, ≥1, or ≥2 Myddosome complexes, for individual biological replicates shown in Fig. 5C. Violin plots indicate the distribution of individual cell measurements. Colored dots superimposed on the violin plots are the averages from individual cell measurements (n = cells for replicates 1-6: n = 29,17,45,17,48 and 35). Bar represents mean. In total a minority of MyD88 puncta recruit TRAF6 (“All”, replicates 1-6: 13.5%, 5.3%, 23.0%, 18.1%, 16.7% and 15.9%). The percentage of TRAF6 recruitment was low for MyD88 puncta containing <1 Myddosome complex (“<1”, replicates 1-6: 3.3%, 1.0%, 3.3%, 13.3%, 7.3% and 8.4%), but increased with the number of complexes per puncta (“≥1”, replicates 1-6: 34.3%, 15.5%, 33.9%, 55.5%, 38.3% and 39.6%; “≥2”, replicates 1-6: 61.3%, 43.4%, 50.7%, 85.0%, 55.7% and 54.0%).

B. Percentage (%) of MyD88-GFP puncta that colocalizes with HOIL1 per cell for all puncta, and puncta categorized as containing <1, ≥1, or ≥2 Myddosome complexes, for individual biological replicates shown in Fig. 5C. Violin plots indicate the distribution of individual cell measurements. Colored dots superimposed on the violin plots are the averages from individual cell measurements (n = cells for replicates 1-9: n = 19, 35, 46, 34, 9, 15, 21, 27 and 24). Bar represents mean. In total a minority of MyD88 puncta recruit HOIL1 (“All”, replicates 1-6: 7.0%, 9.3%, 8.4%, 9.1%, 9.7%, 9.2%, 5.5%, 10.0% and 9.2%). The percentage of HOIL1 recruitment was low for MyD88 puncta containing <1 Myddosome complex (“<1”, replicates 1-6: 0.06%, 0.01%, 0.04%, 0.52%, 0.26%, 0.34%, 0.11%, 1.2% and 1.4%), but increased with the number of complexes per puncta (“≥1”, replicates 1-6: 10.2%, 13.8%, 13.0%, 21.1%, 11.4%, 12.8%, 6.8%, 20.9% and 24.1%; “≥2”, replicates 1-6: 16.2%, 20.4%, 21.1%, 42.2%, 13.5%, 17.4%, 9.4%, 41.9% and 50.7%).

Appendix Figure S5

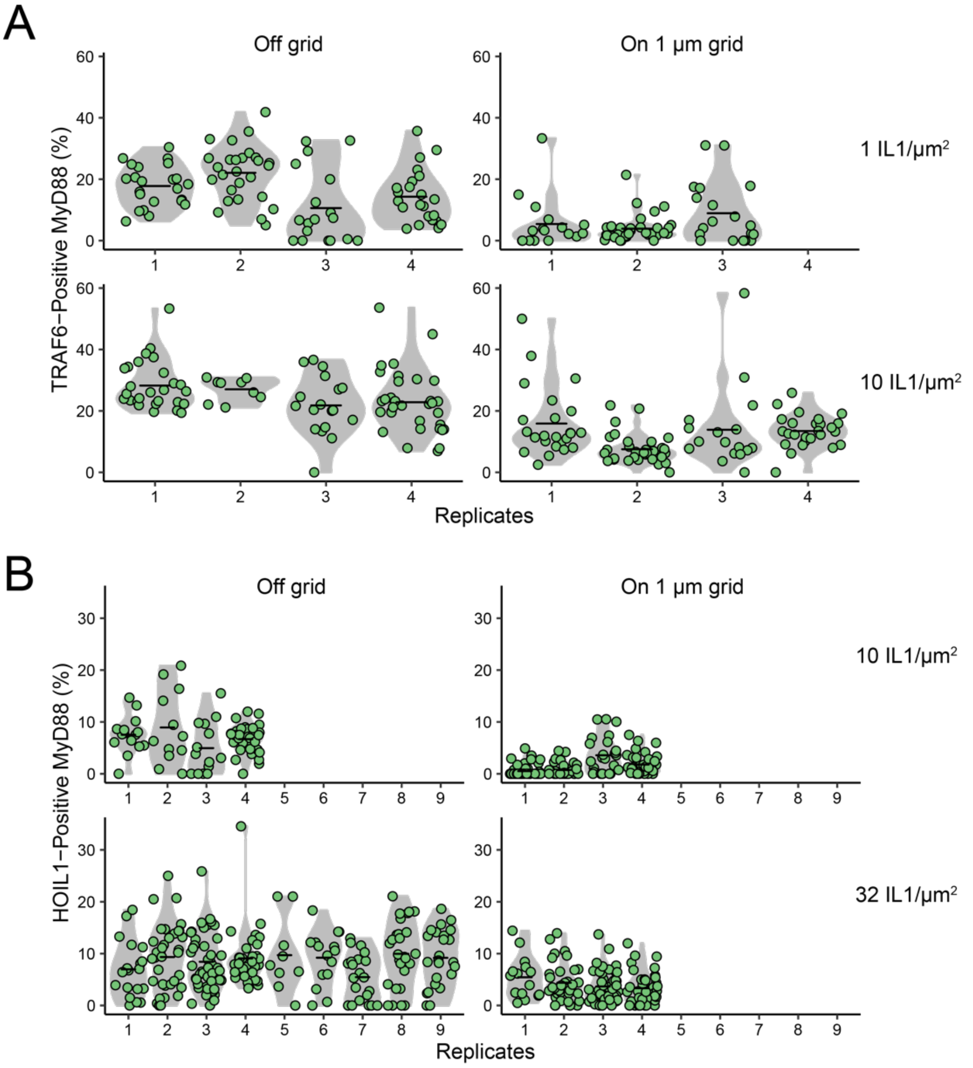

Appendix Figure S5: Biological replicate data from the characterization of dynamics of MyD88-GFP/mScarlet-TRAF6 cells off grids and on 1 µm grids.

A. Percentage of MyD88-GFP puncta that colocalizes with TRAF6 off grids and on 1 µm grids at 1 or 10 IL1/µm^2 per cell across biological replicates shown in Fig. 6C and 6F. Violin plots indicate the distribution of individual cell measurements. Colored dots superimposed on the violin plots are the averages from individual cell measurements. Bars represent means.

B. Percentage of MyD88-GFP puncta that colocalizes with TRAF6 off grids and on 1 µm grids at 1 or 10 IL1/µm^2 per cell across biological replicates in Fig. 6I and 6L. Violin plots indicate the distribution of individual cell measurements. Colored dots superimposed on the violin plots are the averages from individual cell measurements. Bars represent means.
