# Supplementary figures and images for: Myddosome clustering in IL‐1 receptor signaling regulates the formation of an NF‐kB activating signalosome (part 1 of 3)
Source: EMBO Rep. 2023 Aug 21;24(10):e57233. doi: 10.15252/embr.202357233 (PMC10561168; doi:10.15252/embr.202357233)

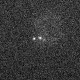

Supplement: Supplementary file 13 — Source Data for Figure 1 [file EMBR-24-e57233-s014.zip › Figure 1/1A/20211217 MyD88-GFP_RawImage.tif]

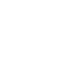

Supplement: Supplementary file 13 — Source Data for Figure 1 [file EMBR-24-e57233-s014.zip › Figure 1/1A/20211217 MyD88-GFP_BackgroundSubtracted.tif]

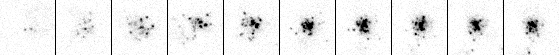

Supplement: Supplementary file 13 — Source Data for Figure 1 [file EMBR-24-e57233-s014.zip › Figure 1/1A/Montage_MyD88.tif]

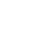

Supplement: Supplementary file 13 — Source Data for Figure 1 [file EMBR-24-e57233-s014.zip › Figure 1/1A/F1_8_15_22_29_34_39_44_49_54.tif]

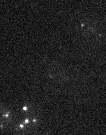

Supplement: Supplementary file 13 — Source Data for Figure 1 [file EMBR-24-e57233-s014.zip › Figure 1/1D-E/20210528 MyD88-GFP_RawImage.tif]

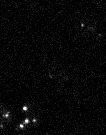

Supplement: Supplementary file 13 — Source Data for Figure 1 [file EMBR-24-e57233-s014.zip › Figure 1/1D-E/20210528 MyD88-GFP_BackgroundSubtracted.tif]

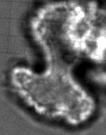

Supplement: Supplementary file 13 — Source Data for Figure 1 [file EMBR-24-e57233-s014.zip › Figure 1/1D-E/BF_F51_rgb.tif]

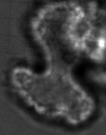

Supplement: Supplementary file 13 — Source Data for Figure 1 [file EMBR-24-e57233-s014.zip › Figure 1/1D-E/BF_F51.tif]

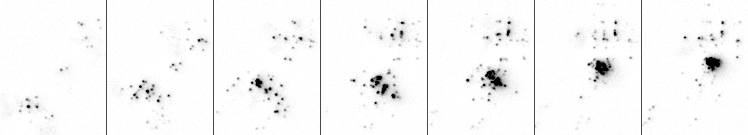

Supplement: Supplementary file 13 — Source Data for Figure 1 [file EMBR-24-e57233-s014.zip › Figure 1/1D-E/Montage_increment15_startfromF15_invertLUT_rgb.tif]

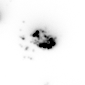

Supplement: Supplementary file 13 — Source Data for Figure 1 [file EMBR-24-e57233-s014.zip › Figure 1/1C/MyD88_Off_2.5um_1um_inverted.tif]

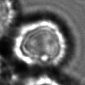

Supplement: Supplementary file 13 — Source Data for Figure 1 [file EMBR-24-e57233-s014.zip › Figure 1/1C/Brighfield_Off_2.5um_1um.tif]

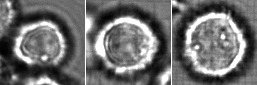

Supplement: Supplementary file 13 — Source Data for Figure 1 [file EMBR-24-e57233-s014.zip › Figure 1/1C/Montage_gridBF.tif]

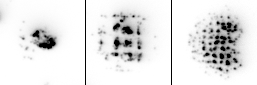

Supplement: Supplementary file 13 — Source Data for Figure 1 [file EMBR-24-e57233-s014.zip › Figure 1/1C/Montage_invert.tif]

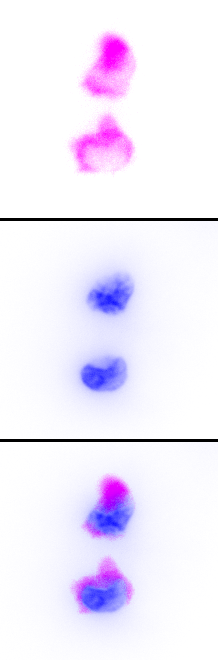

Supplement: Supplementary file 13 — Source Data for Figure 1 [file EMBR-24-e57233-s014.zip › Figure 1/1G/Unstimulated/Unstimulated_Montage.tif]

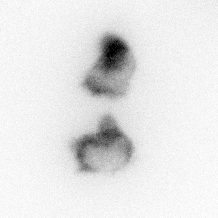

Supplement: Supplementary file 13 — Source Data for Figure 1 [file EMBR-24-e57233-s014.zip › Figure 1/1G/Unstimulated/20220222 568RelA_DAPI_unsti.tif]

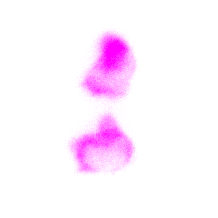

Supplement: Supplementary file 13 — Source Data for Figure 1 [file EMBR-24-e57233-s014.zip › Figure 1/1G/Unstimulated/20220222 568RelA_DAPI_unsti (RGB).tif]

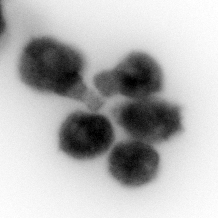

Supplement: Supplementary file 13 — Source Data for Figure 1 [file EMBR-24-e57233-s014.zip › Figure 1/1G/Stimulated/20220315 568RelA_DAPI_Off.tif]

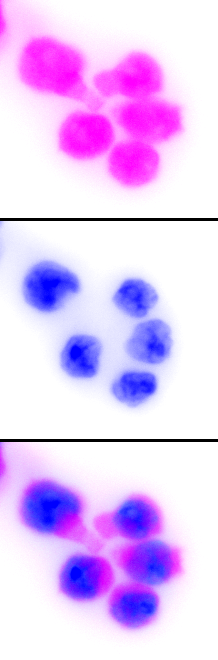

Supplement: Supplementary file 13 — Source Data for Figure 1 [file EMBR-24-e57233-s014.zip › Figure 1/1G/Stimulated/Montage.tif]

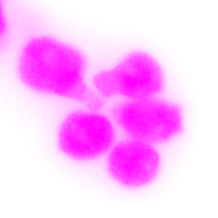

Supplement: Supplementary file 13 — Source Data for Figure 1 [file EMBR-24-e57233-s014.zip › Figure 1/1G/Stimulated/20220315 568RelA_DAPI_Off (RGB).tif]

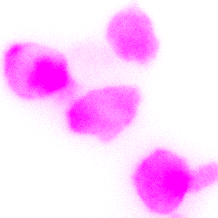

Supplement: Supplementary file 13 — Source Data for Figure 1 [file EMBR-24-e57233-s014.zip › Figure 1/1G/2.5um grid/20220222_568RelA_DAPI_2.5um (RGB).tif]

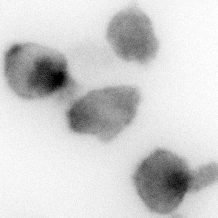

Supplement: Supplementary file 13 — Source Data for Figure 1 [file EMBR-24-e57233-s014.zip › Figure 1/1G/2.5um grid/20220222_568RelA_DAPI_2.5um.tif]

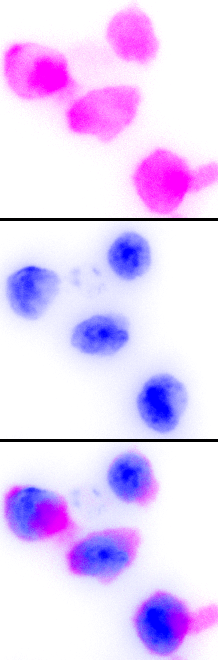

Supplement: Supplementary file 13 — Source Data for Figure 1 [file EMBR-24-e57233-s014.zip › Figure 1/1G/2.5um grid/2.5um_Montage.tif]

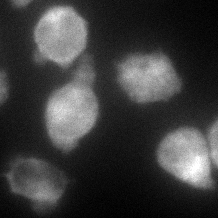

Supplement: Supplementary file 13 — Source Data for Figure 1 [file EMBR-24-e57233-s014.zip › Figure 1/1G/1um grid/20220222 568RelA_DAPI 1um.tif]

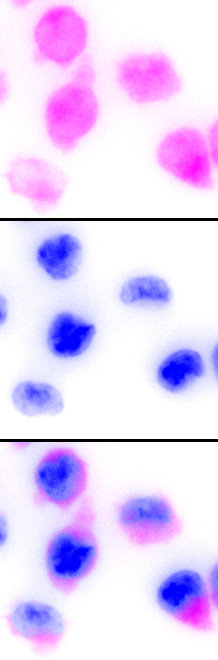

Supplement: Supplementary file 13 — Source Data for Figure 1 [file EMBR-24-e57233-s014.zip › Figure 1/1G/1um grid/1um_grid_Montage.tif]

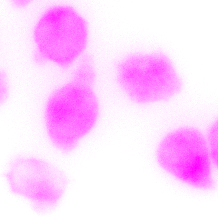

Supplement: Supplementary file 13 — Source Data for Figure 1 [file EMBR-24-e57233-s014.zip › Figure 1/1G/1um grid/20220222 568RelA_DAPI 1um (RGB).tif]

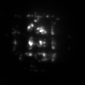

Supplement: Supplementary file 13 — Source Data for Figure 1 [file EMBR-24-e57233-s014.zip › Figure 1/1C/2.5┬╡m/2.5┬╡m.tif]

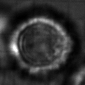

Supplement: Supplementary file 13 — Source Data for Figure 1 [file EMBR-24-e57233-s014.zip › Figure 1/1C/2.5┬╡m/2.5┬╡m-BF.tif]

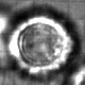

Supplement: Supplementary file 13 — Source Data for Figure 1 [file EMBR-24-e57233-s014.zip › Figure 1/1C/2.5┬╡m/2.5┬╡m-BF_rgb.tif]

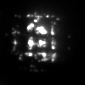

Supplement: Supplementary file 13 — Source Data for Figure 1 [file EMBR-24-e57233-s014.zip › Figure 1/1C/2.5┬╡m/2.5┬╡m_rgb.tif]

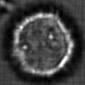

Supplement: Supplementary file 13 — Source Data for Figure 1 [file EMBR-24-e57233-s014.zip › Figure 1/1C/1┬╡m/1┬╡m-BF.tif]

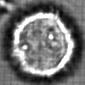

Supplement: Supplementary file 13 — Source Data for Figure 1 [file EMBR-24-e57233-s014.zip › Figure 1/1C/1┬╡m/1┬╡m-BF_rgb.tif]

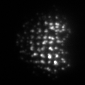

Supplement: Supplementary file 13 — Source Data for Figure 1 [file EMBR-24-e57233-s014.zip › Figure 1/1C/1┬╡m/1┬╡m.tif]

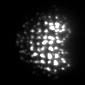

Supplement: Supplementary file 13 — Source Data for Figure 1 [file EMBR-24-e57233-s014.zip › Figure 1/1C/1┬╡m/1┬╡m_rgb.tif]

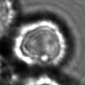

Supplement: Supplementary file 13 — Source Data for Figure 1 [file EMBR-24-e57233-s014.zip › Figure 1/1C/Off/off grid_BF_rgb.tif]

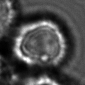

Supplement: Supplementary file 13 — Source Data for Figure 1 [file EMBR-24-e57233-s014.zip › Figure 1/1C/Off/off grid_BF.tif]

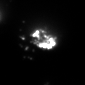

Supplement: Supplementary file 13 — Source Data for Figure 1 [file EMBR-24-e57233-s014.zip › Figure 1/1C/Off/off grid_rgb.tif]

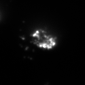

Supplement: Supplementary file 13 — Source Data for Figure 1 [file EMBR-24-e57233-s014.zip › Figure 1/1C/Off/off grid.tif]

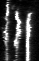

Supplement: Supplementary file 13 — Source Data for Figure 1 [file EMBR-24-e57233-s014.zip › Figure 1/1D-E/Kymograph/on grid/Reslice of on F40-100 rgb.tif]

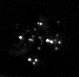

Supplement: Supplementary file 13 — Source Data for Figure 1 [file EMBR-24-e57233-s014.zip › Figure 1/1D-E/Kymograph/on grid/on F40-100.tif]

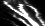

Supplement: Supplementary file 13 — Source Data for Figure 1 [file EMBR-24-e57233-s014.zip › Figure 1/1D-E/Kymograph/off grid/Reslice of F30-55_rgb.tif]

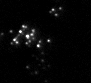

Supplement: Supplementary file 13 — Source Data for Figure 1 [file EMBR-24-e57233-s014.zip › Figure 1/1D-E/Kymograph/off grid/F30-55.tif]

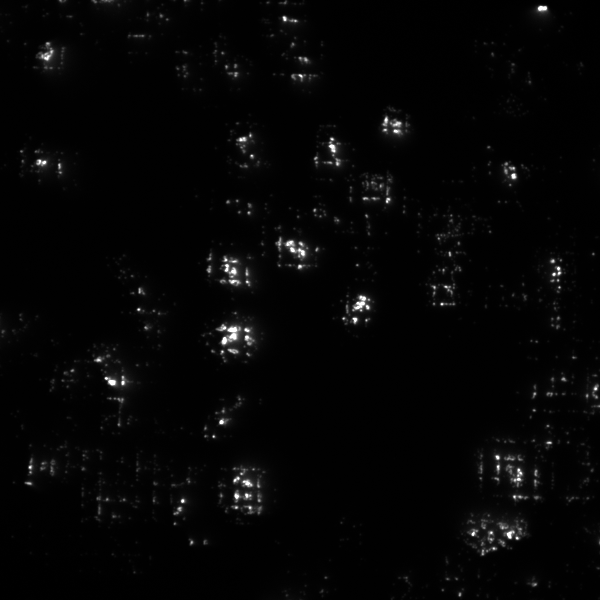

Supplement: Supplementary file 13 — Source Data for Figure 1 [file EMBR-24-e57233-s014.zip › Figure 1/1C/2.5┬╡m/crop the cell/20210731_MyD88_Brightfield_2.5um.tif]

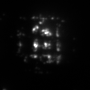

Supplement: Supplementary file 13 — Source Data for Figure 1 [file EMBR-24-e57233-s014.zip › Figure 1/1C/2.5┬╡m/crop the cell/crop the cell.tif]

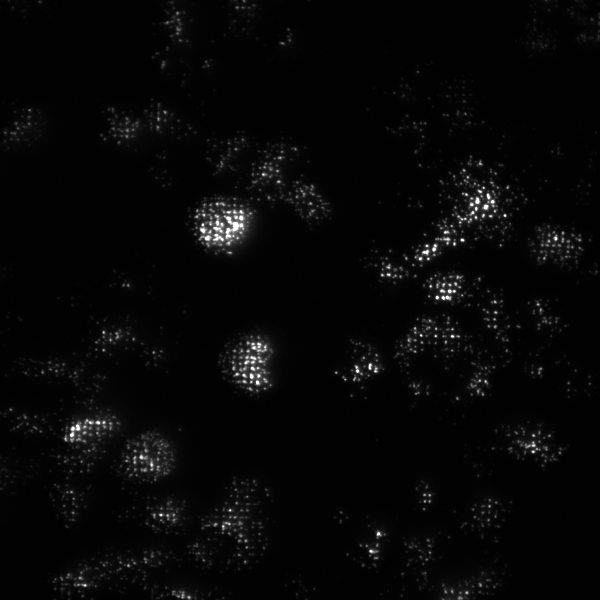

Supplement: Supplementary file 13 — Source Data for Figure 1 [file EMBR-24-e57233-s014.zip › Figure 1/1C/1┬╡m/crop the cell/20210731_MyD88_Brightfield_1um.tif]

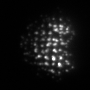

Supplement: Supplementary file 13 — Source Data for Figure 1 [file EMBR-24-e57233-s014.zip › Figure 1/1C/1┬╡m/crop the cell/crop the cell.tif]

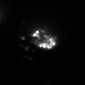

Supplement: Supplementary file 13 — Source Data for Figure 1 [file EMBR-24-e57233-s014.zip › Figure 1/1C/Off/crop the cell/crop the cell.tif]

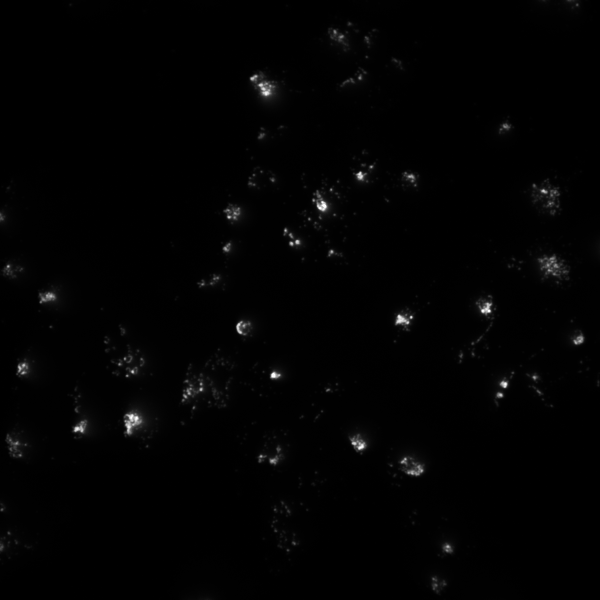

Supplement: Supplementary file 13 — Source Data for Figure 1 [file EMBR-24-e57233-s014.zip › Figure 1/1C/Off/crop the cell/20210731_MyD88_Brightfield_Off.tif]

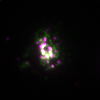

Supplement: Supplementary file 14 — Source Data for Figure 2 [file EMBR-24-e57233-s005.zip › Figure 2/2A/pIKK/Composite.tif]

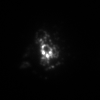

Supplement: Supplementary file 14 — Source Data for Figure 2 [file EMBR-24-e57233-s005.zip › Figure 2/2A/pIKK/20210301_028-3E10_gfpMyD88_647pIKK_Brightfield.tif]

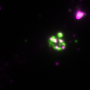

Supplement: Supplementary file 14 — Source Data for Figure 2 [file EMBR-24-e57233-s005.zip › Figure 2/2A/M1/Composite.tif]

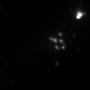

Supplement: Supplementary file 14 — Source Data for Figure 2 [file EMBR-24-e57233-s005.zip › Figure 2/2A/M1/20200929_028-3E10_AF647-M1_MyD88-GFP_Brightfield.tif]

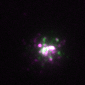

Supplement: Supplementary file 14 — Source Data for Figure 2 [file EMBR-24-e57233-s005.zip › Figure 2/2A/pp65/Composite.tif]

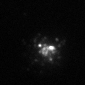

Supplement: Supplementary file 14 — Source Data for Figure 2 [file EMBR-24-e57233-s005.zip › Figure 2/2A/pp65/20220209 568pp65_gfpMyD88_Brightfield.tif]

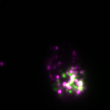

Supplement: Supplementary file 14 — Source Data for Figure 2 [file EMBR-24-e57233-s005.zip › Figure 2/2A/K63/Composite.tif]

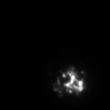

Supplement: Supplementary file 14 — Source Data for Figure 2 [file EMBR-24-e57233-s005.zip › Figure 2/2A/K63/20210301_028-3E10_gfpMyD88_647K63_Brightfield.tif]

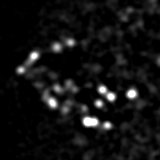

Supplement: Supplementary file 14 — Source Data for Figure 2 [file EMBR-24-e57233-s005.zip › Figure 2/2C/xy view/pIKK_MyD88_Merge_160*160 (RGB).tif]

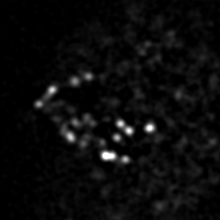

Supplement: Supplementary file 14 — Source Data for Figure 2 [file EMBR-24-e57233-s005.zip › Figure 2/2C/xy view/20210820_028-3E10_568pIKK_gfpMyD88_DAPI_SIM3Dleap_220*220.tif]

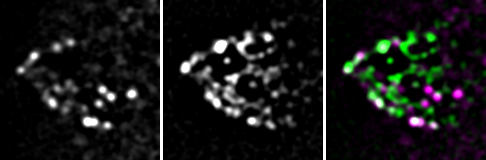

Supplement: Supplementary file 14 — Source Data for Figure 2 [file EMBR-24-e57233-s005.zip › Figure 2/2C/xy view/Montage_pIKK_MyD88_Merge.tif]

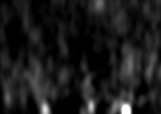

Supplement: Supplementary file 14 — Source Data for Figure 2 [file EMBR-24-e57233-s005.zip › Figure 2/2C/xz view/Reslice-flipped (RGB).tif]

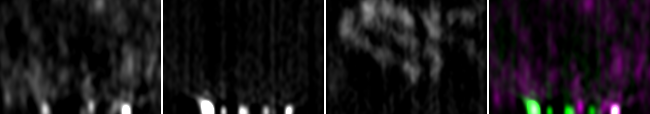

Supplement: Supplementary file 14 — Source Data for Figure 2 [file EMBR-24-e57233-s005.zip › Figure 2/2C/xz view/Montage_reslice.tif]

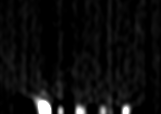

Supplement: Supplementary file 14 — Source Data for Figure 2 [file EMBR-24-e57233-s005.zip › Figure 2/2C/xz view/C2-Reslice-flipped.tif]

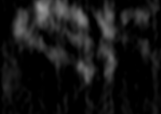

Supplement: Supplementary file 14 — Source Data for Figure 2 [file EMBR-24-e57233-s005.zip › Figure 2/2C/xz view/C3-Reslice-flipped.tif]

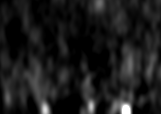

Supplement: Supplementary file 14 — Source Data for Figure 2 [file EMBR-24-e57233-s005.zip › Figure 2/2C/xz view/C1-Reslice-flipped.tif]

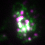

Supplement: Supplementary file 14 — Source Data for Figure 2 [file EMBR-24-e57233-s005.zip › Figure 2/2B/pIKK/Inset_Composite.tif]

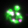

Supplement: Supplementary file 14 — Source Data for Figure 2 [file EMBR-24-e57233-s005.zip › Figure 2/2B/M1/Composite (RGB).tif]

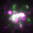

Supplement: Supplementary file 14 — Source Data for Figure 2 [file EMBR-24-e57233-s005.zip › Figure 2/2B/pp65/Composite.tif]

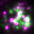

Supplement: Supplementary file 14 — Source Data for Figure 2 [file EMBR-24-e57233-s005.zip › Figure 2/2B/K63/Inset_Composite (RGB).tif]

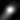

Supplement: Supplementary file 14 — Source Data for Figure 2 [file EMBR-24-e57233-s005.zip › Figure 2/2C/xy view/crop spots/crop1.tif]

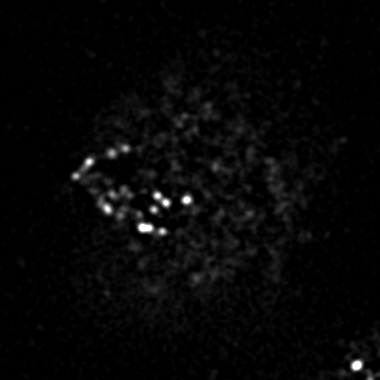

Supplement: Supplementary file 14 — Source Data for Figure 2 [file EMBR-24-e57233-s005.zip › Figure 2/2C/xy view/crop spots/crop2.tif]

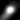

Supplement: Supplementary file 14 — Source Data for Figure 2 [file EMBR-24-e57233-s005.zip › Figure 2/2C/xy view/crop spots/crop1 (RGB).tif]

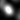

Supplement: Supplementary file 14 — Source Data for Figure 2 [file EMBR-24-e57233-s005.zip › Figure 2/2C/xy view/crop spots/crop2 (RGB).tif]

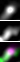

Supplement: Supplementary file 14 — Source Data for Figure 2 [file EMBR-24-e57233-s005.zip › Figure 2/2C/xy view/crop spots/Montage_crop1.tif]

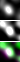

Supplement: Supplementary file 14 — Source Data for Figure 2 [file EMBR-24-e57233-s005.zip › Figure 2/2C/xy view/crop spots/Montage_crop2.tif]

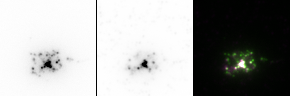

Supplement: Supplementary file 15 — Source Data for Figure 3 [file EMBR-24-e57233-s018.zip › Figure 3/3B/pIKK_off grid/montage_myd88_pikk_merge-rgb.tif]

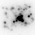

Supplement: Supplementary file 15 — Source Data for Figure 3 [file EMBR-24-e57233-s018.zip › Figure 3/3B/pIKK_off grid/Inset MyD88-rgb.tif]

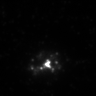

Supplement: Supplementary file 15 — Source Data for Figure 3 [file EMBR-24-e57233-s018.zip › Figure 3/3B/pIKK_off grid/20220513 568pIKK_gfpMyD88_NC1 005_ggggg_crop.tif]

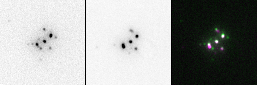

Supplement: Supplementary file 15 — Source Data for Figure 3 [file EMBR-24-e57233-s018.zip › Figure 3/3C/on 1um grid/montage_myd88_pp65_merge.tif]

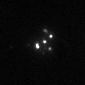

Supplement: Supplementary file 15 — Source Data for Figure 3 [file EMBR-24-e57233-s018.zip › Figure 3/3C/on 1um grid/20220209 568pp65_gfpMyD88_grid3_1um 045_ggg-crop.tif]

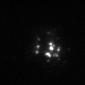

Supplement: Supplementary file 15 — Source Data for Figure 3 [file EMBR-24-e57233-s018.zip › Figure 3/3C/on 2.5um grid/20220216 568pp65_gfpMyD88_grid3_2p5um 009_ggggg_crop.tif]

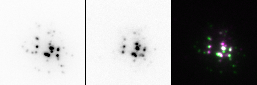

Supplement: Supplementary file 15 — Source Data for Figure 3 [file EMBR-24-e57233-s018.zip › Figure 3/3C/on 2.5um grid/myd88_pp65_merge.tif]

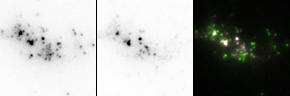

Supplement: Supplementary file 15 — Source Data for Figure 3 [file EMBR-24-e57233-s018.zip › Figure 3/3D/2.5um grid/montage_myd88+pikk+merge-rgb.tif]

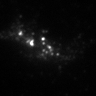

Supplement: Supplementary file 15 — Source Data for Figure 3 [file EMBR-24-e57233-s018.zip › Figure 3/3D/2.5um grid/20220513 568pIKK_gfpMyD88_Grid2_2p5um 041_gg_crop.tif]

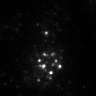

Supplement: Supplementary file 15 — Source Data for Figure 3 [file EMBR-24-e57233-s018.zip › Figure 3/3D/1um grid/20220513 568pIKK_gfpMyD88_Grid3_1um 025_gg_crop.tif]

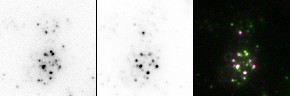

Supplement: Supplementary file 15 — Source Data for Figure 3 [file EMBR-24-e57233-s018.zip › Figure 3/3D/1um grid/montage_myd88_pikk_merge-rgb.tif]

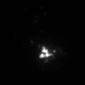

Supplement: Supplementary file 15 — Source Data for Figure 3 [file EMBR-24-e57233-s018.zip › Figure 3/3A/pp65_off grid/20220216 568pp65_gfpMyD88_NC4 008_ggg_crop.tif]

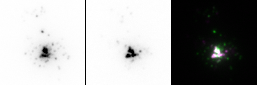

Supplement: Supplementary file 15 — Source Data for Figure 3 [file EMBR-24-e57233-s018.zip › Figure 3/3A/pp65_off grid/montage_myd88_pp65_merge.tif]

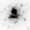

Supplement: Supplementary file 15 — Source Data for Figure 3 [file EMBR-24-e57233-s018.zip › Figure 3/3A/pp65_off grid/Inset MyD88-rgb.tif]

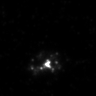

Supplement: Supplementary file 15 — Source Data for Figure 3 [file EMBR-24-e57233-s018.zip › Figure 3/3B/pIKK_off grid/Background subtracted pIKK_fire LUT/fire_0-15000-crop.tif]

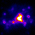

Supplement: Supplementary file 15 — Source Data for Figure 3 [file EMBR-24-e57233-s018.zip › Figure 3/3B/pIKK_off grid/Background subtracted pIKK_fire LUT/fire_0-15000-crop-crop-rgb.tif]

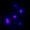

Supplement: Supplementary file 15 — Source Data for Figure 3 [file EMBR-24-e57233-s018.zip › Figure 3/3C/on 1um grid/Background subtracted pp65_fire LUT/1um crop 0-4000_crop rgb-crop.tif]

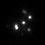

Supplement: Supplementary file 15 — Source Data for Figure 3 [file EMBR-24-e57233-s018.zip › Figure 3/3C/on 1um grid/Background subtracted pp65_fire LUT/1um crop 0-4000_crop.tif]

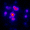

Supplement: Supplementary file 15 — Source Data for Figure 3 [file EMBR-24-e57233-s018.zip › Figure 3/3C/on 2.5um grid/Background subtracted pp65_fire LUT/2.5um fire 0-4000_crop rgb-crop.tif]

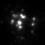

Supplement: Supplementary file 15 — Source Data for Figure 3 [file EMBR-24-e57233-s018.zip › Figure 3/3C/on 2.5um grid/Background subtracted pp65_fire LUT/2.5um fire 0-4000_crop.tif]

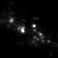

Supplement: Supplementary file 15 — Source Data for Figure 3 [file EMBR-24-e57233-s018.zip › Figure 3/3D/2.5um grid/Background subtracted pIKK_fire LUT/crop_0-15000.tif]

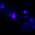

Supplement: Supplementary file 15 — Source Data for Figure 3 [file EMBR-24-e57233-s018.zip › Figure 3/3D/2.5um grid/Background subtracted pIKK_fire LUT/crop_0-15000-rgb-crop.tif]

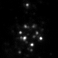

Supplement: Supplementary file 15 — Source Data for Figure 3 [file EMBR-24-e57233-s018.zip › Figure 3/3D/1um grid/Background subtracted pIKK_fire LUT/crop 0-15000.tif]

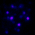

Supplement: Supplementary file 15 — Source Data for Figure 3 [file EMBR-24-e57233-s018.zip › Figure 3/3D/1um grid/Background subtracted pIKK_fire LUT/crop 0-15000-rgb-crop.tif]

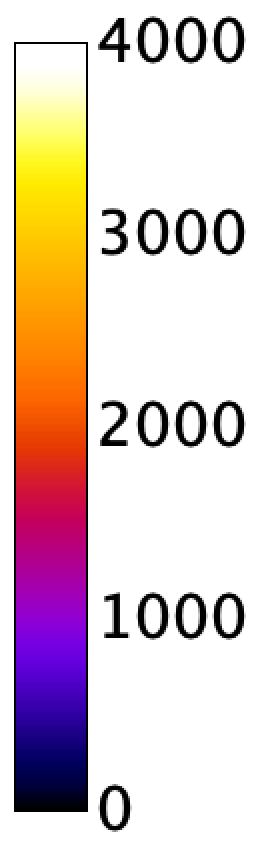

Supplement: Supplementary file 15 — Source Data for Figure 3 [file EMBR-24-e57233-s018.zip › Figure 3/3A/pp65_off grid/Background subtracted pp65_fire LUT/Screen Shot 2022-05-27 at 15.15.53.png]

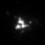

Supplement: Supplementary file 15 — Source Data for Figure 3 [file EMBR-24-e57233-s018.zip › Figure 3/3A/pp65_off grid/Background subtracted pp65_fire LUT/off fire 0-4000_crop.tif]

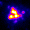

Supplement: Supplementary file 15 — Source Data for Figure 3 [file EMBR-24-e57233-s018.zip › Figure 3/3A/pp65_off grid/Background subtracted pp65_fire LUT/off fire 0-4000_crop rgb_crop.tif]
