# Supplementary figures and images for: Myddosome clustering in IL‐1 receptor signaling regulates the formation of an NF‐kB activating signalosome (part 2 of 3)
Source: EMBO Rep. 2023 Aug 21;24(10):e57233. doi: 10.15252/embr.202357233 (PMC10561168; doi:10.15252/embr.202357233)

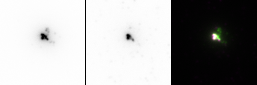

Supplement: Supplementary file 16 — Source Data for Figure 4 [file EMBR-24-e57233-s017.zip › Figure 4/4B/M1_off grid/Montage_myd88-m1-merge-rgb.tif]

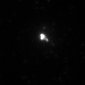

Supplement: Supplementary file 16 — Source Data for Figure 4 [file EMBR-24-e57233-s017.zip › Figure 4/4B/M1_off grid/20220802 568M1_gfpMyD88_NC4 028_crop.tif]

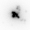

Supplement: Supplementary file 16 — Source Data for Figure 4 [file EMBR-24-e57233-s017.zip › Figure 4/4B/M1_off grid/Inset_MyD88-rgb.tif]

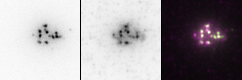

Supplement: Supplementary file 16 — Source Data for Figure 4 [file EMBR-24-e57233-s017.zip › Figure 4/4C/on 1um grid/Montage_myd88_k63_merge-rgb.tif]

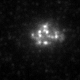

Supplement: Supplementary file 16 — Source Data for Figure 4 [file EMBR-24-e57233-s017.zip › Figure 4/4C/on 1um grid/20220520 568K63_gfpMyD88_grid5 1um 015_gg_crop.tif]

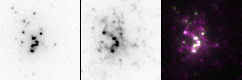

Supplement: Supplementary file 16 — Source Data for Figure 4 [file EMBR-24-e57233-s017.zip › Figure 4/4C/on 2.5um grid/Montage_myd88_k63_merge-rgb.tif]

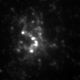

Supplement: Supplementary file 16 — Source Data for Figure 4 [file EMBR-24-e57233-s017.zip › Figure 4/4C/on 2.5um grid/20220309 568K63_gfpMyD88_Grid5_2p5um 037_ggg_crop.tif]

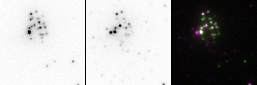

Supplement: Supplementary file 16 — Source Data for Figure 4 [file EMBR-24-e57233-s017.zip › Figure 4/4D/on 1um grid/montage_myd88_m1_merge-rgb.tif]

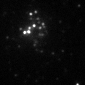

Supplement: Supplementary file 16 — Source Data for Figure 4 [file EMBR-24-e57233-s017.zip › Figure 4/4D/on 1um grid/20220309 568M1_gfpMyD88_Grid3_1um 025_gg_crop.tif]

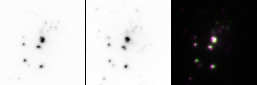

Supplement: Supplementary file 16 — Source Data for Figure 4 [file EMBR-24-e57233-s017.zip › Figure 4/4D/on 2.5um grid/Montage_myd88_m1_merge-rgb.tif]

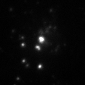

Supplement: Supplementary file 16 — Source Data for Figure 4 [file EMBR-24-e57233-s017.zip › Figure 4/4D/on 2.5um grid/20220309 568M1_gfpMyD88_Grid3_2p5um 021_ggg_crop.tif]

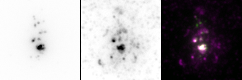

Supplement: Supplementary file 16 — Source Data for Figure 4 [file EMBR-24-e57233-s017.zip › Figure 4/4A/K63_off grid/montage_myd88_k63_merge-rgb.tif]

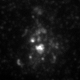

Supplement: Supplementary file 16 — Source Data for Figure 4 [file EMBR-24-e57233-s017.zip › Figure 4/4A/K63_off grid/20220309 568K63_gfpMyD88_NC4 011_g_crop.tif]

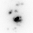

Supplement: Supplementary file 16 — Source Data for Figure 4 [file EMBR-24-e57233-s017.zip › Figure 4/4A/K63_off grid/Inset_MyD88-rgb.tif]

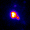

Supplement: Supplementary file 16 — Source Data for Figure 4 [file EMBR-24-e57233-s017.zip › Figure 4/4B/M1_off grid/Background subtracted M1_fire LUT/C1_M1-crop the cell_fire_0-4400_crop-rgb.tif]

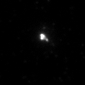

Supplement: Supplementary file 16 — Source Data for Figure 4 [file EMBR-24-e57233-s017.zip › Figure 4/4B/M1_off grid/Background subtracted M1_fire LUT/C1_M1-crop the cell_fire_0-4400.tif]

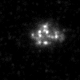

Supplement: Supplementary file 16 — Source Data for Figure 4 [file EMBR-24-e57233-s017.zip › Figure 4/4C/on 1um grid/Background subtracted K63_fire LUT/fire 0-4000-crop.tif]

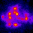

Supplement: Supplementary file 16 — Source Data for Figure 4 [file EMBR-24-e57233-s017.zip › Figure 4/4C/on 1um grid/Background subtracted K63_fire LUT/fire 0-4000-crop-crop-rgb.tif]

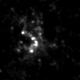

Supplement: Supplementary file 16 — Source Data for Figure 4 [file EMBR-24-e57233-s017.zip › Figure 4/4C/on 2.5um grid/Background subtracted K63_fire LUT/fire 0-4000-crop.tif]

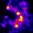

Supplement: Supplementary file 16 — Source Data for Figure 4 [file EMBR-24-e57233-s017.zip › Figure 4/4C/on 2.5um grid/Background subtracted K63_fire LUT/fire 0-4000-crop-crop-rgb.tif]

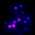

Supplement: Supplementary file 16 — Source Data for Figure 4 [file EMBR-24-e57233-s017.zip › Figure 4/4D/on 1um grid/Background subtracted M1_fire LUT/fire 0-4400-crop-crop-rgb.tif]

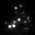

Supplement: Supplementary file 16 — Source Data for Figure 4 [file EMBR-24-e57233-s017.zip › Figure 4/4D/on 1um grid/Background subtracted M1_fire LUT/fire 0-4400-crop-crop.tif]

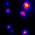

Supplement: Supplementary file 16 — Source Data for Figure 4 [file EMBR-24-e57233-s017.zip › Figure 4/4D/on 2.5um grid/Background subtracted M1_fire LUT/fire 0-4400-crop-crop-rgb.tif]

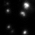

Supplement: Supplementary file 16 — Source Data for Figure 4 [file EMBR-24-e57233-s017.zip › Figure 4/4D/on 2.5um grid/Background subtracted M1_fire LUT/fire 0-4400-crop-crop.tif]

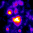

Supplement: Supplementary file 16 — Source Data for Figure 4 [file EMBR-24-e57233-s017.zip › Figure 4/4A/K63_off grid/Background subtracted K63_fire LUT/crop fire 0-4000-crop-rgb.tif]

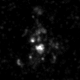

Supplement: Supplementary file 16 — Source Data for Figure 4 [file EMBR-24-e57233-s017.zip › Figure 4/4A/K63_off grid/Background subtracted K63_fire LUT/crop fire 0-4000.tif]

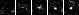

Supplement: Supplementary file 17 — Source Data for Figure 5 [file EMBR-24-e57233-s012.zip › Figure 5/5A/Montage_traf6_F44:55:59:63:67.tif]

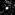

Supplement: Supplementary file 17 — Source Data for Figure 5 [file EMBR-24-e57233-s012.zip › Figure 5/5A/TRAF6_F67.tif]

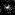

Supplement: Supplementary file 17 — Source Data for Figure 5 [file EMBR-24-e57233-s012.zip › Figure 5/5A/TRAF6_F59.tif]

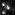

Supplement: Supplementary file 17 — Source Data for Figure 5 [file EMBR-24-e57233-s012.zip › Figure 5/5A/MyD88_F55.tif]

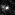

Supplement: Supplementary file 17 — Source Data for Figure 5 [file EMBR-24-e57233-s012.zip › Figure 5/5A/MyD88_F44.tif]

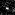

Supplement: Supplementary file 17 — Source Data for Figure 5 [file EMBR-24-e57233-s012.zip › Figure 5/5A/TRAF6_F63.tif]

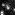

Supplement: Supplementary file 17 — Source Data for Figure 5 [file EMBR-24-e57233-s012.zip › Figure 5/5A/MyD88_F63.tif]

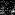

Supplement: Supplementary file 17 — Source Data for Figure 5 [file EMBR-24-e57233-s012.zip › Figure 5/5A/TRAF6_F44.tif]

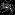

Supplement: Supplementary file 17 — Source Data for Figure 5 [file EMBR-24-e57233-s012.zip › Figure 5/5A/TRAF6_F55.tif]

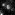

Supplement: Supplementary file 17 — Source Data for Figure 5 [file EMBR-24-e57233-s012.zip › Figure 5/5A/MyD88_F59.tif]

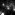

Supplement: Supplementary file 17 — Source Data for Figure 5 [file EMBR-24-e57233-s012.zip › Figure 5/5A/MyD88_F67.tif]

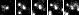

Supplement: Supplementary file 17 — Source Data for Figure 5 [file EMBR-24-e57233-s012.zip › Figure 5/5A/Montage_myd88_F44:55:59:63:67.tif]

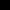

Supplement: Supplementary file 17 — Source Data for Figure 5 [file EMBR-24-e57233-s012.zip › Figure 5/5F/C2-F26.tif]

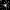

Supplement: Supplementary file 17 — Source Data for Figure 5 [file EMBR-24-e57233-s012.zip › Figure 5/5F/C1-F4.tif]

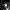

Supplement: Supplementary file 17 — Source Data for Figure 5 [file EMBR-24-e57233-s012.zip › Figure 5/5F/C1-F26.tif]

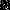

Supplement: Supplementary file 17 — Source Data for Figure 5 [file EMBR-24-e57233-s012.zip › Figure 5/5F/C2-F4.tif]

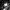

Supplement: Supplementary file 17 — Source Data for Figure 5 [file EMBR-24-e57233-s012.zip › Figure 5/5F/C1-F28.tif]

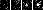

Supplement: Supplementary file 17 — Source Data for Figure 5 [file EMBR-24-e57233-s012.zip › Figure 5/5F/Montage_traf6.tif]

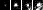

Supplement: Supplementary file 17 — Source Data for Figure 5 [file EMBR-24-e57233-s012.zip › Figure 5/5F/Montage_myd88.tif]

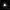

Supplement: Supplementary file 17 — Source Data for Figure 5 [file EMBR-24-e57233-s012.zip › Figure 5/5F/C1-F15.tif]

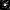

Supplement: Supplementary file 17 — Source Data for Figure 5 [file EMBR-24-e57233-s012.zip › Figure 5/5F/C2-F28.tif]

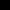

Supplement: Supplementary file 17 — Source Data for Figure 5 [file EMBR-24-e57233-s012.zip › Figure 5/5F/C2-F15.tif]

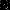

Supplement: Supplementary file 17 — Source Data for Figure 5 [file EMBR-24-e57233-s012.zip › Figure 5/5F/C2-RGB-TRAF6.tif]

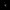

Supplement: Supplementary file 17 — Source Data for Figure 5 [file EMBR-24-e57233-s012.zip › Figure 5/5F/C1-RGB_myd88.tif]

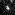

Supplement: Supplementary file 17 — Source Data for Figure 5 [file EMBR-24-e57233-s012.zip › Figure 5/5B/F218-HOIL1.tif]

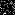

Supplement: Supplementary file 17 — Source Data for Figure 5 [file EMBR-24-e57233-s012.zip › Figure 5/5B/F180-HOIL1.tif]

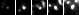

Supplement: Supplementary file 17 — Source Data for Figure 5 [file EMBR-24-e57233-s012.zip › Figure 5/5B/Montage_MyD88_180:190:200:209:218.tif]

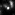

Supplement: Supplementary file 17 — Source Data for Figure 5 [file EMBR-24-e57233-s012.zip › Figure 5/5B/F200-MyD88.tif]

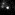

Supplement: Supplementary file 17 — Source Data for Figure 5 [file EMBR-24-e57233-s012.zip › Figure 5/5B/F180-MyD88.tif]

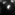

Supplement: Supplementary file 17 — Source Data for Figure 5 [file EMBR-24-e57233-s012.zip › Figure 5/5B/F218-MyD88.tif]

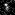

Supplement: Supplementary file 17 — Source Data for Figure 5 [file EMBR-24-e57233-s012.zip › Figure 5/5B/F200-HOIL1.tif]

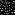

Supplement: Supplementary file 17 — Source Data for Figure 5 [file EMBR-24-e57233-s012.zip › Figure 5/5B/F190-HOIL1.tif]

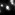

Supplement: Supplementary file 17 — Source Data for Figure 5 [file EMBR-24-e57233-s012.zip › Figure 5/5B/F209-MyD88.tif]

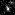

Supplement: Supplementary file 17 — Source Data for Figure 5 [file EMBR-24-e57233-s012.zip › Figure 5/5B/F209-HOIL1.tif]

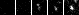

Supplement: Supplementary file 17 — Source Data for Figure 5 [file EMBR-24-e57233-s012.zip › Figure 5/5B/Montage_HOIL1_180:190:200:209:218.tif]

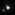

Supplement: Supplementary file 17 — Source Data for Figure 5 [file EMBR-24-e57233-s012.zip › Figure 5/5B/F190-MyD88.tif]

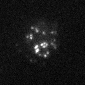

Supplement: Supplementary file 17 — Source Data for Figure 5 [file EMBR-24-e57233-s012.zip › Figure 5/5A/F63/20200123_MyD88_TRAF6_F63_RawImage.tif]

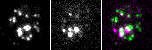

Supplement: Supplementary file 17 — Source Data for Figure 5 [file EMBR-24-e57233-s012.zip › Figure 5/5A/F63/Montage.tif]

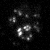

Supplement: Supplementary file 17 — Source Data for Figure 5 [file EMBR-24-e57233-s012.zip › Figure 5/5A/F63/F63_background subtracted.tif]

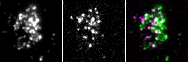

Supplement: Supplementary file 17 — Source Data for Figure 5 [file EMBR-24-e57233-s012.zip › Figure 5/5B/F227/Montage.tif]

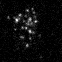

Supplement: Supplementary file 17 — Source Data for Figure 5 [file EMBR-24-e57233-s012.zip › Figure 5/5B/F227/F227_background subtracted.tif]

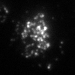

Supplement: Supplementary file 17 — Source Data for Figure 5 [file EMBR-24-e57233-s012.zip › Figure 5/5B/F227/20191205_MyD88_HOIL1_F227_RawImage.tif]

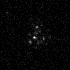

Supplement: Supplementary file 18 — Source Data for Figure 6 [file EMBR-24-e57233-s011.zip › Figure 6/6A/F103off/20211214_MyD88_TRAF6_F103_Background subtracted.tif]

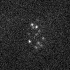

Supplement: Supplementary file 18 — Source Data for Figure 6 [file EMBR-24-e57233-s011.zip › Figure 6/6A/F103off/20211214_TRAF6_MyD88_F103_RawImage.tif]

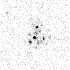

Supplement: Supplementary file 18 — Source Data for Figure 6 [file EMBR-24-e57233-s011.zip › Figure 6/6A/F103off/F103off all rgb.tif]

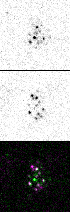

Supplement: Supplementary file 18 — Source Data for Figure 6 [file EMBR-24-e57233-s011.zip › Figure 6/6A/F103off/Montage.tif]

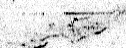

Supplement: Supplementary file 18 — Source Data for Figure 6 [file EMBR-24-e57233-s011.zip › Figure 6/6A/kymograph off/reslice all rgb.tif]

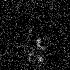

Supplement: Supplementary file 18 — Source Data for Figure 6 [file EMBR-24-e57233-s011.zip › Figure 6/6A/kymograph off/20211214_MyD88_TRAF6.tif]

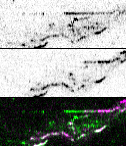

Supplement: Supplementary file 18 — Source Data for Figure 6 [file EMBR-24-e57233-s011.zip › Figure 6/6A/kymograph off/Montage.tif]

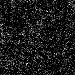

Supplement: Supplementary file 18 — Source Data for Figure 6 [file EMBR-24-e57233-s011.zip › Figure 6/6H/kympgraph/C0-F10-135_hoil1.tif]

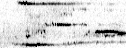

Supplement: Supplementary file 18 — Source Data for Figure 6 [file EMBR-24-e57233-s011.zip › Figure 6/6H/kympgraph/Reslice of F10-135 (invertLUT)-rgb-rotated.tif]

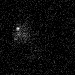

Supplement: Supplementary file 18 — Source Data for Figure 6 [file EMBR-24-e57233-s011.zip › Figure 6/6H/kympgraph/C1-F10-135_myd88.tif]

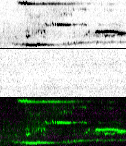

Supplement: Supplementary file 18 — Source Data for Figure 6 [file EMBR-24-e57233-s011.zip › Figure 6/6H/kympgraph/Montage_reslice (invertLUT)-rotated.tif]

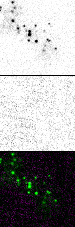

Supplement: Supplementary file 18 — Source Data for Figure 6 [file EMBR-24-e57233-s011.zip › Figure 6/6H/F61/Montage_F61.tif]

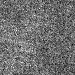

Supplement: Supplementary file 18 — Source Data for Figure 6 [file EMBR-24-e57233-s011.zip › Figure 6/6H/F61/20211217 HOIL1_MyD88_Raw Image.tif]

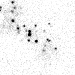

Supplement: Supplementary file 18 — Source Data for Figure 6 [file EMBR-24-e57233-s011.zip › Figure 6/6H/F61/F61.tif]

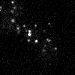

Supplement: Supplementary file 18 — Source Data for Figure 6 [file EMBR-24-e57233-s011.zip › Figure 6/6H/F61/20211217 MyD88_HOIL1_Background subtracted.tif]

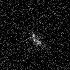

Supplement: Supplementary file 18 — Source Data for Figure 6 [file EMBR-24-e57233-s011.zip › Figure 6/6G/Kymograph/MyD88_F181-306.tif]

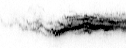

Supplement: Supplementary file 18 — Source Data for Figure 6 [file EMBR-24-e57233-s011.zip › Figure 6/6G/Kymograph/Reslice of 3chs_F181-306_invertLUT-rgb_rotated.tif]

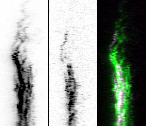

Supplement: Supplementary file 18 — Source Data for Figure 6 [file EMBR-24-e57233-s011.zip › Figure 6/6G/Kymograph/Montage_reslice_F181-306 (invertLUT)-rgb.tif]

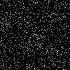

Supplement: Supplementary file 18 — Source Data for Figure 6 [file EMBR-24-e57233-s011.zip › Figure 6/6G/Kymograph/HOIL1_F181-306.tif]

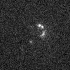

Supplement: Supplementary file 18 — Source Data for Figure 6 [file EMBR-24-e57233-s011.zip › Figure 6/6G/F61/20210528_HOIL1_MyD88_Raw Image.tif]

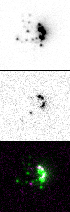

Supplement: Supplementary file 18 — Source Data for Figure 6 [file EMBR-24-e57233-s011.zip › Figure 6/6G/F61/Montage_F61.tif]

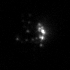

Supplement: Supplementary file 18 — Source Data for Figure 6 [file EMBR-24-e57233-s011.zip › Figure 6/6G/F61/20210528_MyD88_HOIL1_Background subtracted.tif]

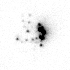

Supplement: Supplementary file 18 — Source Data for Figure 6 [file EMBR-24-e57233-s011.zip › Figure 6/6G/F61/F61-rgb.tif]

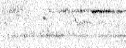

Supplement: Supplementary file 18 — Source Data for Figure 6 [file EMBR-24-e57233-s011.zip › Figure 6/6B/kymograph/reslice all rgb.tif]

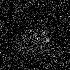

Supplement: Supplementary file 18 — Source Data for Figure 6 [file EMBR-24-e57233-s011.zip › Figure 6/6B/kymograph/20211213_MyD88_TRAF6.tif]

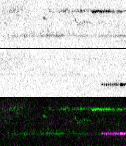

Supplement: Supplementary file 18 — Source Data for Figure 6 [file EMBR-24-e57233-s011.zip › Figure 6/6B/kymograph/Montage.tif]

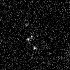

Supplement: Supplementary file 18 — Source Data for Figure 6 [file EMBR-24-e57233-s011.zip › Figure 6/6B/F103/20211213_MyD88_TRAF6_Background subtracted.tif]

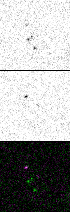

Supplement: Supplementary file 18 — Source Data for Figure 6 [file EMBR-24-e57233-s011.zip › Figure 6/6B/F103/Montage.tif]

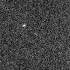

Supplement: Supplementary file 18 — Source Data for Figure 6 [file EMBR-24-e57233-s011.zip › Figure 6/6B/F103/20211213_TRAF6_MyD88_RawImage.tif]

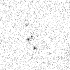

Supplement: Supplementary file 18 — Source Data for Figure 6 [file EMBR-24-e57233-s011.zip › Figure 6/6B/F103/F103-all rgb.tif]

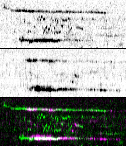

Supplement: Supplementary file 18 — Source Data for Figure 6 [file EMBR-24-e57233-s011.zip › Figure 6/6E/Kymograph/Montage_rotated.tif]

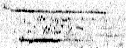

Supplement: Supplementary file 18 — Source Data for Figure 6 [file EMBR-24-e57233-s011.zip › Figure 6/6E/Kymograph/Reslice of F81-206 (invertLUT)-rgb_rotate.tif]
