# Supplementary figures and images for: Myddosome clustering in IL‐1 receptor signaling regulates the formation of an NF‐kB activating signalosome (part 3 of 3)
Source: EMBO Rep. 2023 Aug 21;24(10):e57233. doi: 10.15252/embr.202357233 (PMC10561168; doi:10.15252/embr.202357233)

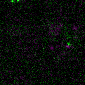

Supplement: Supplementary file 18 — Source Data for Figure 6 [file EMBR-24-e57233-s011.zip › Figure 6/6E/Kymograph/F81-206_merge_rgb.tif]

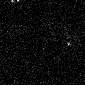

Supplement: Supplementary file 18 — Source Data for Figure 6 [file EMBR-24-e57233-s011.zip › Figure 6/6E/Kymograph/F81-206_MyD88.tif]

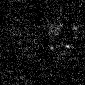

Supplement: Supplementary file 18 — Source Data for Figure 6 [file EMBR-24-e57233-s011.zip › Figure 6/6E/Kymograph/F81-206_TRAF6.tif]

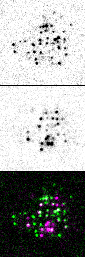

Supplement: Supplementary file 18 — Source Data for Figure 6 [file EMBR-24-e57233-s011.zip › Figure 6/6E/F61/Montage_F61.tif]

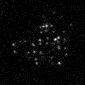

Supplement: Supplementary file 18 — Source Data for Figure 6 [file EMBR-24-e57233-s011.zip › Figure 6/6E/F61/20211218 MyD88_TRAF6_background subtracted.tif]

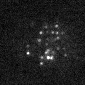

Supplement: Supplementary file 18 — Source Data for Figure 6 [file EMBR-24-e57233-s011.zip › Figure 6/6E/F61/20211218 MyD88_TRAF6_Raw Image.tif]

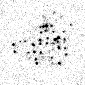

Supplement: Supplementary file 18 — Source Data for Figure 6 [file EMBR-24-e57233-s011.zip › Figure 6/6E/F61/F61.tif]

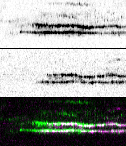

Supplement: Supplementary file 18 — Source Data for Figure 6 [file EMBR-24-e57233-s011.zip › Figure 6/6K/Kymograph/Montage_Reslice of F38-163.tif]

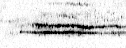

Supplement: Supplementary file 18 — Source Data for Figure 6 [file EMBR-24-e57233-s011.zip › Figure 6/6K/Kymograph/Reslice of F38-163 all rgb.tif]

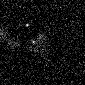

Supplement: Supplementary file 18 — Source Data for Figure 6 [file EMBR-24-e57233-s011.zip › Figure 6/6K/Kymograph/MyD88_HOIL1_F38-163.tif]

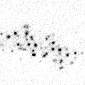

Supplement: Supplementary file 18 — Source Data for Figure 6 [file EMBR-24-e57233-s011.zip › Figure 6/6K/F117/F117 all rgb.tif]

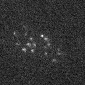

Supplement: Supplementary file 18 — Source Data for Figure 6 [file EMBR-24-e57233-s011.zip › Figure 6/6K/F117/20210804 HOIL1_MyD88_Raw Image.tif]

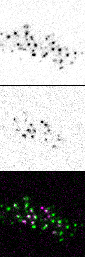

Supplement: Supplementary file 18 — Source Data for Figure 6 [file EMBR-24-e57233-s011.zip › Figure 6/6K/F117/Montage F117.tif]

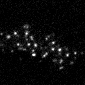

Supplement: Supplementary file 18 — Source Data for Figure 6 [file EMBR-24-e57233-s011.zip › Figure 6/6K/F117/20210804 MyD88_HOIL1_Background subtracted.tif]

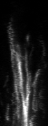

Supplement: Supplementary file 18 — Source Data for Figure 6 [file EMBR-24-e57233-s011.zip › Figure 6/6J/Kymograph/Reslice of F125-250.tif]

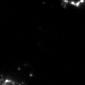

Supplement: Supplementary file 18 — Source Data for Figure 6 [file EMBR-24-e57233-s011.zip › Figure 6/6J/Kymograph/MyD88_HOIL1_F125-250.tif]

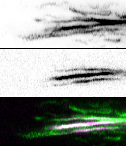

Supplement: Supplementary file 18 — Source Data for Figure 6 [file EMBR-24-e57233-s011.zip › Figure 6/6J/Kymograph/Montage reslice.tif]

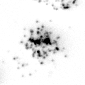

Supplement: Supplementary file 18 — Source Data for Figure 6 [file EMBR-24-e57233-s011.zip › Figure 6/6J/F117/F117 all rgb.tif]

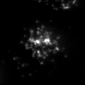

Supplement: Supplementary file 18 — Source Data for Figure 6 [file EMBR-24-e57233-s011.zip › Figure 6/6J/F117/20191205 MyD88_HOIL1_Background subtracted.tif]

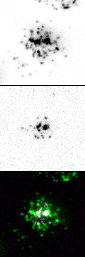

Supplement: Supplementary file 18 — Source Data for Figure 6 [file EMBR-24-e57233-s011.zip › Figure 6/6J/F117/Montage.tif]

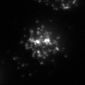

Supplement: Supplementary file 18 — Source Data for Figure 6 [file EMBR-24-e57233-s011.zip › Figure 6/6J/F117/20191205 HOIL1_MyD88_Raw Image.tif]

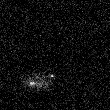

Supplement: Supplementary file 18 — Source Data for Figure 6 [file EMBR-24-e57233-s011.zip › Figure 6/6D/kymograph/20211207 MyD88_TRAF6.tif]

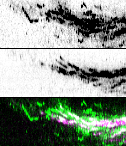

Supplement: Supplementary file 18 — Source Data for Figure 6 [file EMBR-24-e57233-s011.zip › Figure 6/6D/kymograph/Montage_rotated.tif]

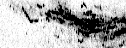

Supplement: Supplementary file 18 — Source Data for Figure 6 [file EMBR-24-e57233-s011.zip › Figure 6/6D/kymograph/Reslice of F1-126_merge_invertLUT-rgb-rotated.tif]

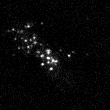

Supplement: Supplementary file 18 — Source Data for Figure 6 [file EMBR-24-e57233-s011.zip › Figure 6/6D/F61/20211207 MyD88_TRAF6_background subtracted.tif]

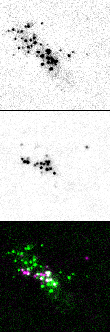

Supplement: Supplementary file 18 — Source Data for Figure 6 [file EMBR-24-e57233-s011.zip › Figure 6/6D/F61/F61_Montage.tif]

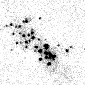

Supplement: Supplementary file 18 — Source Data for Figure 6 [file EMBR-24-e57233-s011.zip › Figure 6/6D/F61/F61.tif]

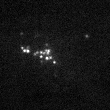

Supplement: Supplementary file 18 — Source Data for Figure 6 [file EMBR-24-e57233-s011.zip › Figure 6/6D/F61/20211207 MyD88_TRAF6_Raw Image.tif]
