# Supplementary material for: Myddosome clustering in IL‐1 receptor signaling regulates the formation of an NF‐kB activating signalosome
Source: EMBO Rep. 2023 Aug 21;24(10):e57233. doi: 10.15252/embr.202357233 (PMC10561168; doi:10.15252/embr.202357233)
Supplement: Supplementary file 20 [file EMBR-24-e57233-s015.pdf]

EMBO reports

Myddosome clustering in IL-1 receptor signaling regulates the formation of an NF-kB signalosome

Fakun Cao, Rafael Deliz-Aguirre, Fenja Gerpott, Elke Ziska, and Marcus Taylor
DOI: 10.15252/embr.202357233

Corresponding author(s): Marcus Taylor (taylor@mpiib-berlin.mpg.de)

Review Timeline:

Submission Date:	24th Mar 23
Editorial Decision:	24th May 23
Revision Received:	1st Jun 23
Editorial Decision:	17th Jul 23
Revision Received:	20th Jul 23
Accepted:	27th Jul 23

Editor: Martina Rembold

Transaction Report:

(Note: With the exception of the correction of typographical or spelling errors that could be a source of ambiguity, letters and reports are not edited. Depending on transfer agreements, referee reports obtained elsewhere may or may not be included in this compilation. Referee reports are anonymous unless the Referee chooses to sign their reports.)

1st Editorial Decision **24th May 2023**

Dear Marcus,

Thank you once more for the submission of your research manuscript to our journal. As you know, we have now received the full set of referee reports and I have already forwarded you the reports (copied again below).

As you will see, the referees acknowledge that the findings are potentially interesting, but they also raise a number of concerns that need to be addressed in the revision.

Given these constructive comments, we would like to invite you to revise your manuscript with the understanding that the referee concerns (as detailed above and in their reports) must be fully addressed and their suggestions taken on board. Please address all referee concerns in a complete point-by-point response. Acceptance of the manuscript will depend on a positive outcome of a second round of review. It is EMBO reports policy to allow a single round of revision only and acceptance or rejection of the manuscript will therefore depend on the completeness of your responses included in the next, final version of the manuscript.

We realize that it is difficult to revise to a specific deadline. In the interest of protecting the conceptual advance provided by the work, we recommend a revision within 3 months (August 24). Please discuss the revision progress ahead of this time with the editor if you require more time to complete the revisions.

I am also happy to discuss the revision further via e-mail or a video call, if you wish.

*****IMPORTANT NOTE:
We perform an initial quality control of all revised manuscripts before re-review. Your manuscript will FAIL this control and the handling will be DELAYED if the following APPLIES:

1) A data availability section providing access to data deposited in public databases is missing. If you have not deposited any data, please add a sentence to the data availability section that explains that.

2) Your manuscript contains statistics and error bars based on n=2. Please use scatter blots in these cases. No statistics should be calculated if n=2.

When submitting your revised manuscript, please carefully review the instructions that follow below. Failure to include requested items will delay the evaluation of your revision.*****

When submitting your revised manuscript, we will require:

1) a .docx formatted version of the manuscript text (including legends for main figures, EV figures and tables). Please make sure that the changes are highlighted to be clearly visible.

2) individual production quality figure files as .eps, .tif, .jpg (one file per figure).
Please download our Figure Preparation Guidelines (figure preparation pdf) from our Author Guidelines pages https://www.embopress.org/page/journal/14693178/authorguide for more info on how to prepare your figures.

3) a .docx formatted letter INCLUDING the reviewers' reports and your detailed point-by-point responses to their comments. As part of the EMBO Press transparent editorial process, the point-by-point response is part of the Review Process File (RPF), which will be published alongside your paper.

4) a complete author checklist, which you can download from our author guidelines (<https://www.embopress.org/page/journal/14693178/authorguide>). Please insert information in the checklist that is also reflected in the manuscript. The completed author checklist will also be part of the RPF.

5) Please note that all corresponding authors are required to supply an ORCID ID for their name upon submission of a revised manuscript (<https://orcid.org/>). Please find instructions on how to link your ORCID ID to your account in our manuscript tracking system in our Author guidelines
(<https://www.embopress.org/page/journal/14693178/authorguide#authorshipguidelines>)

6) We replaced Supplementary Information with Expanded View (EV) Figures and Tables that are collapsible/expandable online. A maximum of 5 EV Figures can be typeset. EV Figures should be cited as 'Figure EV1, Figure EV2" etc... in the text and their respective legends should be included in the main text after the legends of regular figures.

- For the figures that you do NOT wish to display as Expanded View figures, they should be bundled together with their legends in a single PDF file called *Appendix*, which should start with a short Table of Content. Appendix figures should be referred to in the main text as: "Appendix Figure S1, Appendix Figure S2" etc. See detailed instructions regarding expanded view here: <https://www.embopress.org/page/journal/14693178/authorguide#expandedview>

- Additional Tables/Datasets should be labeled and referred to as Table EV1, Dataset EV1, etc. Legends have to be provided in a separate tab in case of .xls files. Alternatively, the legend can be supplied as a separate text file (README) and zipped together with the Table/Dataset file.

7) Please note that a Data Availability section at the end of Materials and Methods is now mandatory. In case you have no data that requires deposition in a public database, please state so instead of refereeing to the database.

=>In your case, please refer to the scripts deposited on github in this section.

See also < https://www.embopress.org/page/journal/14693178/authorguide#dataavailability>). Please note that the Data Availability Section is restricted to new primary data that are part of this study.

8) At EMBO Press we ask authors to provide source data for the main figures. Our source data coordinator will contact you to discuss which figure panels we would need source data for and will also provide you with helpful tips on how to upload and organize the files.

Additional information on source data and instruction on how to label the files are available <https://www.embopress.org/page/journal/14693178/authorguide#sourcedata>.

9) The journal requires a statement specifying whether or not authors have competing interests (defined as all potential or actual interests that could be perceived to influence the presentation or interpretation of an article). In case of competing interests, this must be specified in your disclosure statement. Further information: https://www.embopress.org/competing-interests

10) Figure legends and data quantification:
The following points must be specified in each figure legend:

- the name of the statistical test used to generate error bars and P values,
- the number (n) of independent experiments (please specify technical or biological replicates) underlying each data point,
- the nature of the bars and error bars (s.d., s.e.m.)

- If the data are obtained from n {less than or equal to} 5, show the individual data points in addition to the SD or SEM.
- If the data are obtained from n {less than or equal to} 2, use scatter blots showing the individual data points.

Discussion of statistical methodology can be reported in the materials and methods section, but figure legends should contain a basic description of n, P and the test applied.

See also the guidelines for figure legend preparation:
https://www.embopress.org/page/journal/14693178/authorguide#figureformat

- Please also include scale bars in all microscopy images.

11) Please use our template for the 'Reagents and Tools' table, which you can find on our Guide to Authors, keyword "Structured methods".

12) Our journal encourages inclusion of *data citations in the reference list* to directly cite datasets that were re-used and obtained from public databases. Data citations in the article text are distinct from normal bibliographical citations and should directly link to the database records from which the data can be accessed. In the main text, data citations are formatted as follows: "Data ref: Smith et al, 2001" or "Data ref: NCBI Sequence Read Archive PRJNA342805, 2017". In the Reference list, data citations must be labeled with "[DATASET]". A data reference must provide the database name, accession number/identifiers and a resolvable link to the landing page from which the data can be accessed at the end of the reference. Further instructions are available at <https://www.embopress.org/page/journal/14693178/authorguide#referencesformat>.

13) As part of the EMBO publication's Transparent Editorial Process, EMBO reports publishes online a Review Process File to accompany accepted manuscripts. This File will be published in conjunction with your paper and will include the referee reports, your point-by-point response and all pertinent correspondence relating to the manuscript.

You are able to opt out of this by letting the editorial office know (emboreports@embo.org). If you do opt out, the Review Process File link will point to the following statement: "No Review Process File is available with this article, as the authors have chosen not to make the review process public in this case."

We would also welcome the submission of cover suggestions, or motifs to be used by our Graphics Illustrator in designing a cover.

I look forward to seeing a revised form of your manuscript when it is ready and please let me know if you have questions or comments regarding the revision.

Kind regards,

Martina

Martina Rembold, PhD
Senior Editor
EMBO reports

****

Referee #1:

This is an interesting paper that investigates the role of IL-1R clustering in recruitment of signaling effectors such as MyD88, TRAF6 and HOIL using a supported lipid bilayer model to present IL-1. An argument is made for a threshold cluster size for MyD88 recruitment and separate threshold for recruitment of E3 ligase TRAF6 and HOIL, which generate poly-Ub chains that are required for down-stream signaling, explains the switchlike and analog aspects of IL-1R signaling. Initial Myddosome formation generates a switch-like response whereas the further growth and confluence of clusters is required to recruit HOIL. An important tool in the study is the use of chrome grids to limit the mobility of the bilayer bound IL-1 and thus the mobility of the Myddosomes. The barriers both control the about of IL-1 available to each Myddosome and also limits the ability of the Myddosomes to "diffuse" in the bilayers to collect into larger aggregates. This is an interesting and worthwhile dataset, but I have some questions to clarification.

1. The detailed topology of the system is unclear to me and looking back at the earlier JCB paper didn't address this. The typical interpretation of microcluster formation in interfaces is that small cell projections initiate contacts where receptor and ligands can diffuse into the point of contact and accumulate to form the microcluster . Various factors may control if these clusters can expand in area, move or coalesce. In this system it looks like these points of contact remain discrete, display a mix of random and directed movements, particularly to the center of the cell, and group together. But in this grouping process, it's not clear if they remain as a discrete bundle of protrusion or form a larger, consolidated area of close contact in which all the microclusters effectively merge. I think the only way to address this is electron microscopy, although a 3D super-resolution fluorescence method may provide some insight, I'm speptical that 3D SIM or dSTORM would be fully interpretable. There may also be a process leading to extracellular vesicle formation as an alternative to endocytosis.
2. The authors acknowledge that an important effect of the grids is to limit the ability to IL-1 to diffuse into the microclusters to allow growth. They show that effects of HOIL recruitment can be overcome by increasing the density to 32 molecules/µm2. A limitation of the data set is that they don't track the IL-1 at all, as they did in the original study in JCB. It would be important to know how many IL-1 are recruited to the microclusters off grid vs on grid. In the JCB paper that authors put up 300+ IL-1 /µm2 , which might saturate the Myddosomes even on the 1x1 µm grids. I suspect this would fully overcome the need for the Myddosomes to coalesce to achieve the stronger NFkB activation. Therefore, would the authors conclude that the ability of the clusters to coalesce to a single central point is not important as the grid prevents this and any defects in signaling can be overcome by providing more IL-1 per grid compartment to enable formation of larger myddosomes?

Referee #2:

In this MS the authors provide evidence to show that the clustering behavior of Myddosomes provides a basis for digital and analog IL-1R signaling. They provide novel technical evidence to show, for the first time, that Myddosomes are tethered to the cell
surface via direct interaction with the IL-1R bound to extracellular IL-1 and that spatial organization of Myddosomes encodes responses proportional to the amount of IL-1 stimulation.

The authors should address the following issues
1. Fig 1G IL1b off grid is not very clear and therefore does not support the authors conclusions as strongly as it should: can the authors provide a clearer image? The quantitation clearly provides the supporting evidence, but the representative images

should be improved
2. Fig2 A presumably these images were taken off grids? Please can the authors clarify. What time point was used to visualise the Myddosomes? Does the composition of proteins chance depending upon when the complexes are imaged? Time course analysis would be interesting here (although this is addressed to a degree later in the MS)
3. "Interestingly, single Myddosomes off grid had statistically greater mean pp65/pIKK intensity than single Myddosomes formed on grids. We noticed that off grid some of these single Myddosomes with high pp65/pIKK staining intensity were closely associated with Myddosome clusters, this suggested Myddosome clusters could enhance signaling output for adjacent complexes." How confident are the authors that these are individual MyDDosomes? Is it possible that because these complexes have been imaged with TIRF microscopy that there are bridging Myddsome complexes out of the field of view which could explain the difference between off grid and on grid observations? It is important to comment on this in the MS as it's a limitation of the work.

Referee #3:

Overall, I found the manuscript interesting and the two types of experiments presented here are elegant: the reduction of clustering by the nano-patterns and the kinetics of recruitment of Traf6. I was not very convinced by the first part of the manuscript as the clustering data don't show a strong effect, if we take into account a normalization by the number of MyD88 molecules- btu the kinetics data show a clear difference between off-grid and on-grid recruitment and demonstrate a "threshold" in size for activation.

Going through the manuscript and the figures I would like to make the following comments:

The experiment described in Figure 1 is not properly introduced. What triggers the binding and clustering at time 0? Is it cell adhesion at the bottom of the well and contacting the IL-1? Images in Fig1 D suggests that's the case as the cell changes dramatically shape during the 360s of the experiment. If that is the case, it needs to be stated clearly as it seems a major limitation of the setup.

I like the idea of individual "corrals" to limit the diffusion of the MyD88, it is a clever way of demonstrating the importance of clustering. But I do wonder if there is an interaction between the MyD88 proteins or if it is just a physical coupling via the membrane-membrane binding, pushing together the adhesion points independently of the MyD88 attachments. The authors do not seem to discuss why the MyD88 clusters would be attracted to each other...

The experiment in 1G-1H shows a small difference in RelA translocation to the nucleus between cells stimulated on different grids- the difference is not massive and the conclusion from this experiment needs to be taken with a grain of salt.

Experiments in Figure 2C show the colocalization of MyD88 with pIKKa/b
There are some regions with very high MyD88 clustering (especially top right corner) with almost no pIKKa/b. Also, inversely, there are a few clusters of pIKKa/b with almost no MyD88 clustering- so if there is clear co-localisation in some patches, the data are not very convincing.

Experiments in Figure 3 show that pp65 staining or pIKK staining is reduced when the size of the MyD88 cluster is reduced- which makes sense (and in a way the data should be normalized by size of the cluster). What is interesting is the statement that "single Myddosomes off grid had statistically greater mean pp65/pIKK intensity than single Myddosomes formed on grids." I think that the authors should explore this by going to much lower density of functionalised IL-1 SLBs.

Do the authors suggest that larger MyD88 clusters lead to larger recruitment of p65, IKK and K63-Ub? In that case the plot of recruitment vs MyD88 integrated intensity should not be linear but should show a clear inflexion, with higher MyD88 clustering leading to much higher recruitment- this is not obvious from the data. To me it seems that a larger number of MyD88 molecules leads to a higher number of binders, but that the number of recruited protein PER MyD88 DOES NOT CHANGE.
I think this is a strong point of the paper if that's true, but it needs to be investigated in more depth and normalised properly by the signal/number of MyD88 present.

These criticisms are partially erased by the data presented in Figure 5 and Figure 6, Now it becomes obvious that there is a difference between off-grid and on-grid events.

I find the kinetics experiments especially elegant and the results are this time more black and white than the previous col-localization data. There is also data in the supplementary figures on dependence on IL-1 density and the effects are clearer for this part.

The discussion is appropriate and overall the manuscript is well written.

Response to the Referees

We thank the referees for their time, generous comments and helpful suggestions on the manuscript. We are glad that the referees find the dataset and manuscript worthwhile. Below we address the specific referee comments.

Referee #1

This is an interesting paper that investigates the role of IL-1R clustering in recruitment of signaling effectors such as MyD88, TRAF6 and HOIL using a supported lipid bilayer model to present IL-1. An argument is made for a threshold cluster size for MyD88 recruitment and separate threshold for recruitment of E3 ligase TRAF6 and HOIL, which generate poly-Ub chains that are required for down-stream signaling, explains the switchlike and analog aspects of IL-1R signaling. Initial Myddosome formation generates a switch-like response whereas the further growth and confluence of clusters is required to recruit HOIL. An important tool in the study is the use of chrome grids to limit the mobility of the bilayer bound IL-1 and thus the mobility of the Myddosomes. The barriers both control the about of IL-1 available to each Myddosome and also limits the ability of the Myddosomes to "diffuse" in the bilayers to collect into larger aggregates. This is an interesting and worthwhile dataset, but I have some questions to clarification.

We thank the Referee for their insightful comments on the manuscript. We address the specific points below.

1. The detailed topology of the system is unclear to me and looking back at the earlier JCB paper didn't address this. The typical interpretation of microcluster formation in interfaces is that small cell projections initiate contacts where receptor and ligands can diffuse into the point of contact and accumulate to form the microcluster . Various factors may control if these clusters can expand in area, move or coalesce. In this system it looks like these points of contact remain discrete, display a mix of random and directed movements, particularly to the center of the cell, and group together. But in this grouping process, it's not clear if they remain as a discrete bundle of protrusion or form a larger, consolidated area of close contact in which all the microclusters effectively merge. I think the only way to address this is electron microscopy, although a 3D super-resolution fluorescence method may provide some insight, I'm speptical that 3D SIM or dSTORM would be fully interpretable. There may also be a process leading to extracellular vesicle formation as an alternative to endocytosis.

We agree with this comment: the membrane topology relative to these myddosome clusters remains unknown. As pointed out by the referee, an outstanding question is whether it is a consolidated interface or discrete multiple protrusions. Based on our observations, we believe that it is most likely a mixture of both topologies. Due to their high stability, we believe larger clusters are most likely consolidated continuous contact areas with the SLB (like the schematic shown in Fig 1B).

To help clarify this point, we have included an example in Figure EV1 that shows Myddosomes merging and then subsequently splitting (Figure EV1A). We have also produced a schematic that illustrates possible alternative membrane topology (Figure EV1B)

to that shown in Fig 1 and that suggests that at least some clusters are composed of Myddosomes on separate membrane protrusions.
The new panels in Fig EV1A-B:

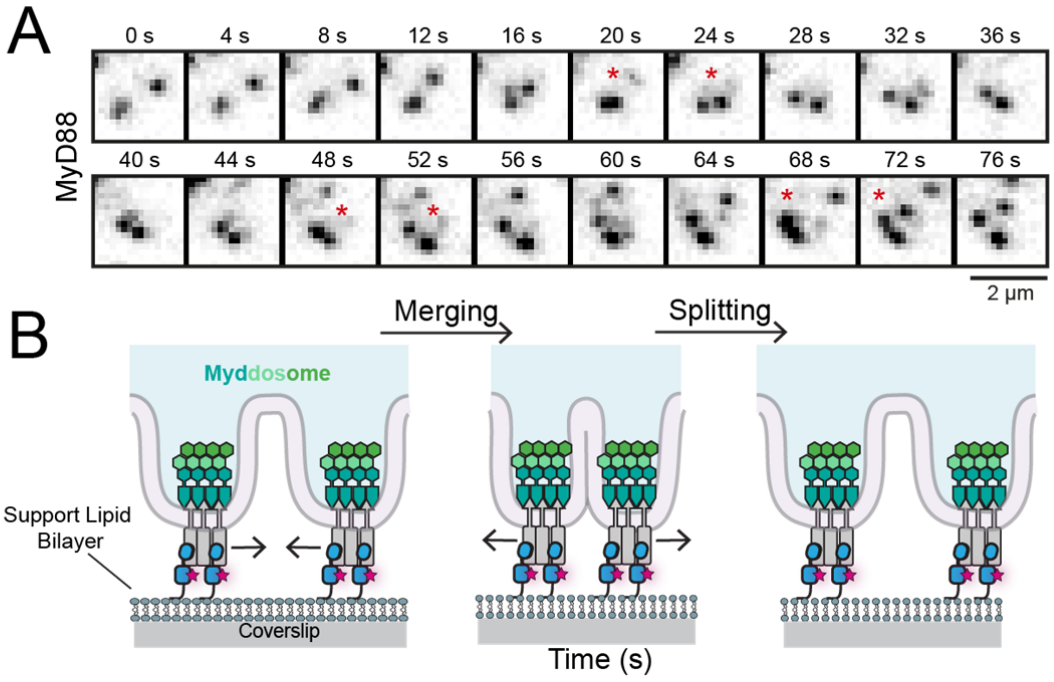

To further clarify in the result section (1st paragraph), we have added the following text.

While many Myddosome clusters were highly stable, in some cases, we observe that Myddosome clustering was not unidirectional, with discrete Myddosomes undergoing merging and subsequent splitting (Fig. EV1B). This unstable nature of a subset of clusters might be because they are composed of discrete Myddosomes confined to separate membrane protrusions and contact zones with the SLB (Fig. EV1B), thus allowing them to split and move apart. Alternatively, more stable Myddosome clusters might be confined to a continuous contact zone with the SLB (Fig. 1B).

We are currently investigating the mechanism that drives cluster formation and the surrounding membrane topology. In future studies we hope to understand the internal physical structure, composition and the surrounding membrane topology and how these regulate the nucleation of the NF-kB signalosome we describe here. We also agree with the reviewer that these clusters might have other functional roles beyond regulating NF-kB signalosome assembly. As pointed out, these could be sites of membrane trafficking, such as vesicle formation or endocytosis. We have added the following text to the discussion to highlight this point (see below under the 2nd point raised by the referee).

2. The authors acknowledge that an important effect of the grids is to limit the ability to IL-1 to diffuse into the microclusters to allow growth. They show that effects of HOIL recruitment can be overcome by increasing the density to 32 molecules/µm2. A limitation of the data set

is that they don't track the IL-1 at all, as they did in the original study in JCB. It would be important to know how many IL-1 are recruited to the microclusters off grid vs on grid. In the JCB paper that authors put up 300+ IL-1 /µm2 , which might saturate the Myddosomes even on the 1x1 µm grids. I suspect this would fully overcome the need for the Myddosomes to coalesce to achieve the stronger NFkB activation. Therefore, would the authors conclude that the ability of the clusters to coalesce to a single central point is not important as the grid prevents this and any defects in signaling can be overcome by providing more IL-1 per grid compartment to enable formation of larger myddosomes?

We agree that higher IL-1 density per grid will induce stronger NF-kB activation. Our interpretation of this is that a greater number of IL-1 per grid allows more Myddosomes to assemble within individual grids, and as they are mobile within the corral they can coalesce into a cluster. This is our interpretation of the data in Figure 7G-L: that changing the IL-1 density can rescue HOIL1 recruitment on grid because there is a sufficient quantity of IL-1 to induce Myddosome that can cluster within the grid corral. In other words the defect that the grids impose on signalling can be overcome by increasing the amount of IL-1.

We agree that we are unable to track IL-1, and this makes it difficult to accurately discern the input/output relationship between the bound IL-1, and the number of assembled Myddosomes that can nucleate signalosomes leading to downstream signalling. We also agree that the ability to form one focal point, or central cluster (as observed off grid), is not as important as forming a cluster when it comes to HOIL1/TRAF6 recruitment.

However, we cannot rule out that the central focal point of Myddosomes observed off grid could have other functional roles that we haven't assayed for (e.g. endocytosis, extracellular vesicle formation or signalling attenuation). To clarify we have added the following text to the discussion:

Myddosome clusters may have other functional roles beyond enhancing NF-kB signalosome formation. Like previous studies (Latty et al. 2018), Myddosomes off grid tend to form a large central focal point (Fig 1A and Fig 2A). As experiments with the nano grids demonstrate (Fig 7), Myddosome clusters can form on grids at higher IL-1 densities, and these clusters can recruit HOIL1 and TRAF6. Despite this rescue of TRAF6/HOIL1 recruitment, the grid still prevents the formation of this large patch-like structure. This structure observed in cells off grid (Fig 1A) possibly plays a role in other downstream signaling processes, such as the internalization of IL-1R-Myddosome complexes by endocytosis or terminating signal transduction. How the spatial organization of Myddosomes regulates other signaling reactions and cellular processes is an avenue for future investigations.

Referee #2

In this MS the authors provide evidence to show that the clustering behavior of Myddosomes provides a basis for digital and analog IL-1R signaling. They provide novel technical evidence to show, for the first time, that Myddosomes are tethered to the cell surface via direct interaction with the IL-1R bound to extracellular IL-1 and that spatial organization of Myddosomes encodes responses proportional to the amount of IL-1 stimulation.

The authors should address the following issues
1. Fig 1G IL1b off grid is not very clear and therefore does not support the authors conclusions as strongly as it should: can the authors provide a clearer image? The quantitation clearly provides the supporting evidence, but the representative images should be improved

We have changed this panel and now present a clearer image that indicates off grid RelA nucleus translocation (see the revised panel in Fig 1G, below and in the manuscript). Please note the inverted colour LUT used in the image was selected to highlight the unactivated cells (e.g. the unstimulated cell in the on/off grid conditions). The invert LUT can make it difficult to discern the colocalization between the DAPI and RelA stain.

Previous panel (note different example image for off grid):

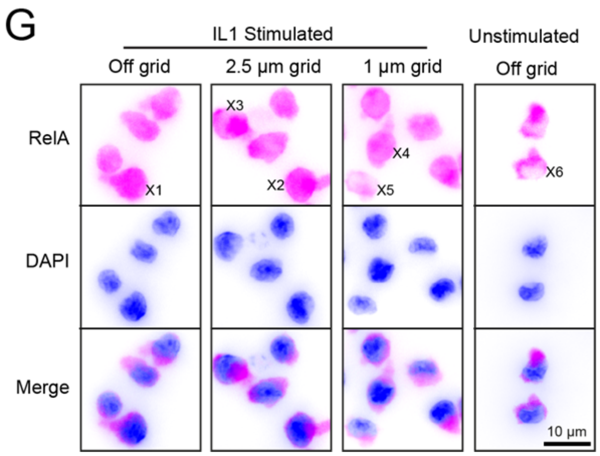

New panel:

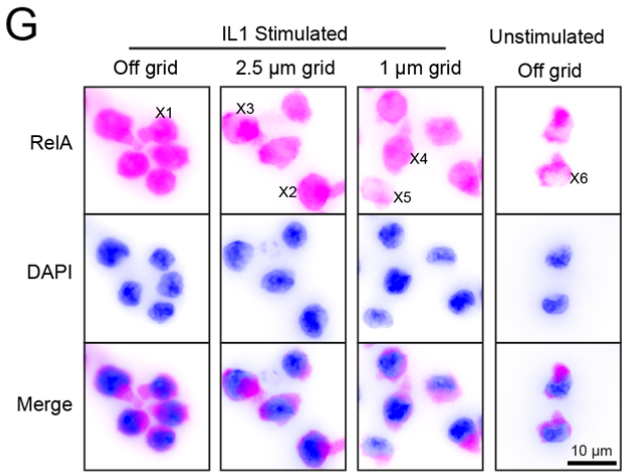

2. Fig2 A presumably these images were taken off grids? Please can the authors clarify. What time point was used to visualise the Myddosomes? Does the composition of proteins chance depending upon when the complexes are imaged? Time course analysis would be interesting here (although this is addressed to a degree later in the MS)

We thank the reviewer for bringing this to our attention. The images in Figure 2 were taken off grid at 30 mins post seeding the cells on the SLB. We have clarified this in the figure 2 legend. The time point of fixation is also indicated in the methods session "Immunofluorescence staining of phospho-p65, phospho-IKK, K63-Ub and M1-Ub" on page 23.

We agree with the reviewer that time course analysis is critical to understand the mechanism of signalosome assembly, and hence the 2nd half of the manuscript focuses on the dynamics of TRAF6 and HOIL1 (as pointed out by the referee).

One technical limitation of Fig 2-4 is the use of fixation to assay signalling read outs. The myddosome and associated signalling complexes in fixed cells represent a mixture of complexes at different time points of assembly (and possibly disassembly). This is because cells have different landing times on the SLB. We believe that a time course analysis to address the question raised requires live cell analysis of labelled subunits of the IKK complex and NF-kB (e.g., NEMO and RelA). Live cell analysis can connect the lifetime and behaviour of Myddosomes to the recruitment and activation of the IKK and NF-kB complexes. In conclusion we believe this analysis would form the basis for future manuscripts and is beyond the scope of the current manuscript.

To be clear about the limitation of the study we have added the following text (start of paragraph under the section of **Larger Myddosome clusters have enhanced TRAF6 and LUBAC recruitment** page 7)**:**

One limitation of immunofluorescence analysis (Fig. 3, and 4) is that the spatial temporal relationship between Myddosome formation, clustering and NF-kB signalosome formation cannot be resolved.

3. "Interestingly, single Myddosomes off grid had statistically greater mean pp65/pIKK intensity than single Myddosomes formed on grids. We noticed that off grid some of these single Myddosomes with high pp65/pIKK staining intensity were closely associated with Myddosome clusters, this suggested Myddosome clusters could enhance signalling output for adjacent complexes." How confident are the authors that these are individual MyDDosomes? Is it possible that because these complexes have been imaged with TIRF microscopy that there are bridging Myddosome complexes out of the field of view which could explain the difference between off grid and on grid observations? It is important to comment on this in the MS as it's a limitation of the work.

We agree with the reviewer and thank them for their insight and highlighting this limitation of the study. We have added this qualifier (new text underlined):

Interestingly, puncta classified as single Myddosomes off grid had statistically greater mean pp65/pIKK intensity than single Myddosomes formed on grids. We noticed that off grid some of these single Myddosomes with high pp65/pIKK staining intensity were closely associated with Myddosome clusters, this suggested Myddosome clusters could enhance signalling output for adjacent complexes. Alternatively these single Myddosomes could be linked or

connected to Myddosome complexes deeper in the cell, that are not illuminated by the TIRF field, and this could also explain the greater staining intensity.

Referee #3

Overall, I found the manuscript interesting and the two types of experiments presented here are elegant: the reduction of clustering by the nano-patterns and the kinetics of recruitment of Traf6. I was not very convinced by the first part of the manuscript as the clustering data don't show a strong effect, if we take into account a normalization by the number of MyD88 molecules- btu the kinetics data show a clear difference between off-grid and on-grid recruitment and demonstrate a "threshold" in size for activation. Going through the manuscript and the figures I would like to make the following comments:

We thank the referee for the thorough review of our manuscript and the helpful comments. We address the specific comments below.

The experiment described in Figure 1 is not properly introduced. What triggers the binding and clustering at time 0? Is it cell adhesion at the bottom of the well and contacting the IL-1? Images in Fig1 D suggests that's the case as the cell changes dramatically shape during the 360s of the experiment. If that is the case, it needs to be stated clearly as it seems a major limitation of the setup.

The initiation of signalling occurs when the cell binds to the IL-1 presented on the SLB. The SLB system we have developed (see Deliz-Aguirre et al. 2021, DOI: 10.1083/jcb.202012071) contains only IL-1, and in this previous manuscript we have imaged the binding of IL-1 by monitoring its mobility and clustering on SLBs. To clarify and properly explain the experimental set up we have added the following text to the first paragraph of the results (new text underlined):

We pipetted EL4 cells expressing MyD88-GFP into chambers containing SLBs and imaged them as they land on this surface and bind to IL-1. We found that MyD88-GFP assembles into puncta at the cell surface (Fig. 1A).

We agree with the referee that IL-1 in this context has an adhesive property and 'tethers' the cell to the SLB. However, we disagree that this is a limitation of the setup. IL-1 signals as both a soluble cytokine and cell surface presented cytokine (see the following references (among others) where cell surface IL-1 is described: (Kaplanski et al. 1994; Kurt-Jones et al. 1985)). Therefore our experimental setup uses SLBs as a biomimetic surface to reconstitute IL-1R signalling at cell:cell junctions. That the SLB only contains IL-1 is a strength of the system as it allows the analysis of IL-1 signal transduction in isolation. In our earlier manuscript, we reported that IL-1 binding precedes the recruitment and assembly of MyD88. Furthermore, while the cell shape may change dramatically (as read out by the brightfield image), the SLB:cell interface and Myddosomes that assemble at it, are stable. This allows us to follow the temporal behaviour of the complexes through time with TIRF microscopy.

t=0 in Fig 1D (and the associated movie) illustrates the beginning of observation of that specific cell, and is used to give the reader the time scale of Myddosome clustering. From

our previous work we know that IL-1 binding preceded this point. We have amended the figure legends to clarify this point. The legend for Figure 1D now reads (new text underlined):

(D) Time series showing Myddosome formation in an EL4 cell interacting with both continuous and 2.5 µm gridded partitioned SLBs. t = 0s denotes the start of cellular observation. Scale bar, 5 µm.

I like the idea of individual "corrals" to limit the diffusion of the MyD88, it is a clever way of demonstrating the importance of clustering. But I do wonder if there is an interaction between the MyD88 proteins or if it is just a physical coupling via the membrane-membrane binding, pushing together the adhesion points independently of the MyD88 attachments. The authors do not seem to discuss why the MyD88 clusters would be attracted to each other...

To clarify, the biochemical basis for MyD88 multimerization is well characterised (see (Lin et al. 2010)). What we address here is the clustering of Myddosomes - the oligomeric complex of MyD88, IRAK4 and IRAK1. To clarify we have added the following to the introduction (new text underlined):

The Myddosome is an oligomeric complex, composed of MyD88, IRAK4 and IRAK1 (Lin et al. 2010), that is crucial for IL-1R signal transduction and an inflammatory innate immune response.

We agree with the referee that an outstanding question that our work raises is what drives clustering and how membrane tethered Myddosomes are attracted to each other. This is an area of ongoing research in the lab, and will form the basis for future publications.

However, before pursuing this question, we wanted to establish the functional role of Myddosome clustering on IL-1 signal transduction, and this is the focus of the current manuscript. We speculate that the attractive force that drives Myddosome clusters could be multifactorial, a mixture of cytoskeleton dynamics or entropy effects from membrane protrusions (similar to that observed in T cell receptor clustering). Therefore, untangling these mechanisms is beyond the scope of the manuscript and will form the basis for future work.

The experiment in 1G-1H shows a small difference in RelA translocation to the nucleus between cells stimulated on different grids- the difference is not massive and the conclusion from this experiment needs to be taken with a grain of salt.

We agree the effect on the RelA translocation to the nucleus is subtle; however, the effect is reproducible across three replicates each containing hundreds of cell measurements. Thus we are confident the result suggests differences in the level of RelA activation. Furthermore our data is consistent with numerous published studies on RelA translocation in innate immune signalling pathways across different cell types. The conclusion of these studies is that subtle changes in the amount of RelA translocation encodes to the level of stimulatory input a cell receives (Son et al. 2022; Son et al. 2021; DeFelice et al. 2019; Tay et al. 2010; Cheng et al. 2021). These subtle changes in nucleus RelA levels have functional consequences at the transcriptional level: small changes can change which genes are

expressed and at what level they are transcribed (see (Cheng et al. 2021; Tay et al. 2010)). In conclusion, we believe this data is consistent with previous analysis on the digital/analog nature of NF-kB signalling in innate immune signalling (including in IL-1 signalling, see (DeFelice et al. 2019; Son et al. 2022)).

Experiments in Figure 2C show the colocalization of MyD88 with pIKKa/b There are some regions with very high MyD88 clustering (especially top right corner) with almost no pIKKa/b. Also, inversely, there are a few clusters of pIKKa/b with almost no MyD88 clustering- so if there is clear co-localisation in some patches, the data are not very convincing.

We believe the referee refers to the SIM images in figure 2C. Since this is fixed cell data and immunofluorescence, this is a snapshot in time. Therefore we cannot know whether these clusters in the top right corner would recruit pIKKa/b or not. However, we presented this image because it shows that pIKKa/b are associated with Myddosome at the cell surface. Furthermore, in some cases the puncta of pIKK only partially overlap with Myddosome. We believe that this data the model of Myddosome as a scaffold that nucleates a NF-kB signalosome. We believe the data in Fig 2C illustrates this insight. Nonetheless, we observe in many cells show strong colocalization of the pIKK puncta and Myddosome, and we have included another SIM image in Appendix 1 to emphasise this point.

New image included in Appendix 1B:

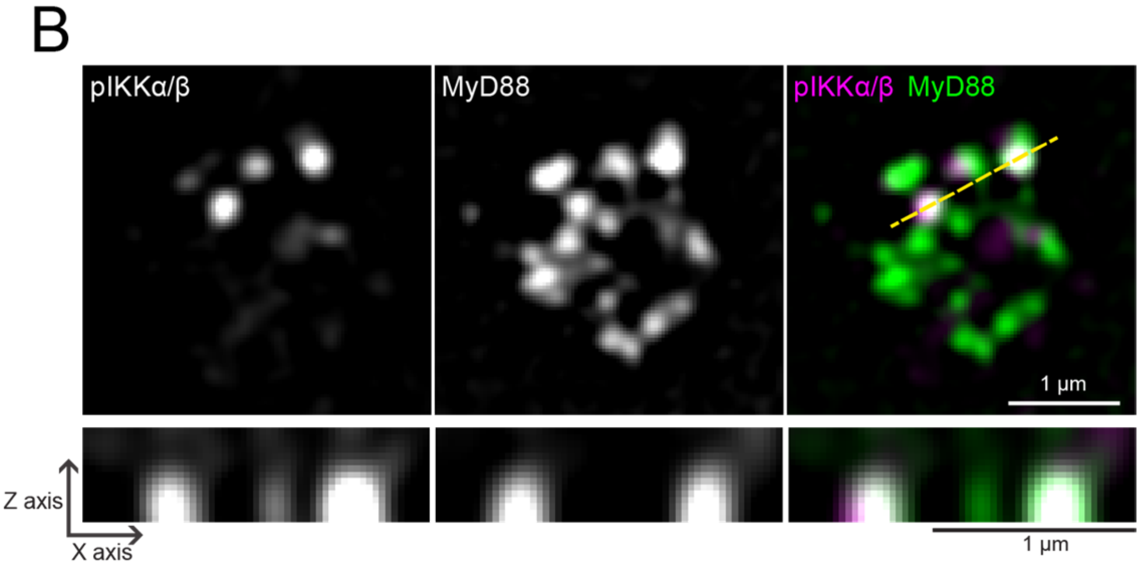

Experiments in Figure 3 show that pp65 staining or pIKK staining is reduced when the size of the MyD88 cluster is reduced- which makes sense (and in a way the data should be normalized by size of the cluster). What is interesting is the statement that "single Myddosomes off grid had statistically greater mean pp65/pIKK intensity than single Myddosomes formed on grids." I think that the authors should explore this by going to much lower density of functionalised IL-1 SLBs.

We agree that it is interesting that single Myddosomes have a greater pp65/pIKK staining off grids, than single Myddosome structures on grid. We observed this effect routinely, and

although we don't have a clear mechanistic explanation, we feel it should be reported in the text. We also note Referee #2's comment that the cause of this could be due to the technical limitation of our experimental setup and have amended the text to clarify this point (see above).

Finally, we believe that the limited temporal resolution of fixation and immunofluorescence staining means it is hard to know the lifecycle of these complexes. The variable landing time of cells on the SLB means that immunofluorescence images consist of Myddosomes that are nucleated at different time points during the 30 mins leading up to fixation. Therefore, it is hard to discern the cause and effect relationship. For example we can not rule out the possibility these small Myddosomes could have possibly split off larger clusters (see response to reviewer 1). Alternatively they could reflect the disassembly of Myddosomes within clusters. Given these technical limitations of immunofluorescence, the mechanistic basis can not be addressed by the referee's suggestion of assaying lower ligand densities with pp65/pIKK immunofluorescence.

Given these possible mechanistic reasons for the observation (and those raised by Referee #2, now included in the text), we believe the answer is being able to observe the full life cycle of Myddosomes, Myddosome clusters and the recruitment and assembly of downstream signalling effectors (specifically IKK complex subunits). This is critical to understanding how Myddosomes activate signalling, and clustering changes nucleation kinetics and the composition of the NF-kB signalosome. This is why the live cell data in Fig. 5-7 is used to complement the immunofluorescence data (a point noted by referee #2).

To fully address the mechanistic basis for why small myddosomes off grid recruit IKK/NF-kB would require live cell analysis and additional labelled cell lines (for example expressing IKK complex subunits, see response to referee #2 point 2). Therefore, we believe this is beyond the scope of the current manuscript, and will form the basis for future studies in the lab.

To address and clarify the limitations of the immunofluorescence data we have added the following text to the manuscript (under the section entitled **Larger Myddosome clusters have enhanced TRAF6 and LUBAC recruitment**):

One limitation of our immunofluorescence analysis (Fig. 3, and 4) is that the spatial temporal relationship between Myddosome formation, clustering and NF-kB signalosome formation can not be resolved.

Do the authors suggest that larger MyD88 clusters lead to larger recruitment of p65, IKK and K63-Ub? In that case the plot of recruitment vs MyD88 integrated intensity should not be linear but should show a clear inflexion, with higher MyD88 clustering leading to much higher recruitment- this is not obvious from the data. To me it seems that a larger number of MyD88 molecules leads to a higher number of binders, but that the number of recruited protein PER MyD88 DOES NOT CHANGE. I think this is a strong point of the paper if that's true, but it needs to be investigated in more depth and normalised properly by the signal/number of MyD88 present.

We thank the referee for raising these points and suggesting this analysis. We agree these are interesting points raised by our work. We have performed the analysis suggested by the referee (normalising the staining intensity by number of Myddosome complexes per puncta):

Figure EV2

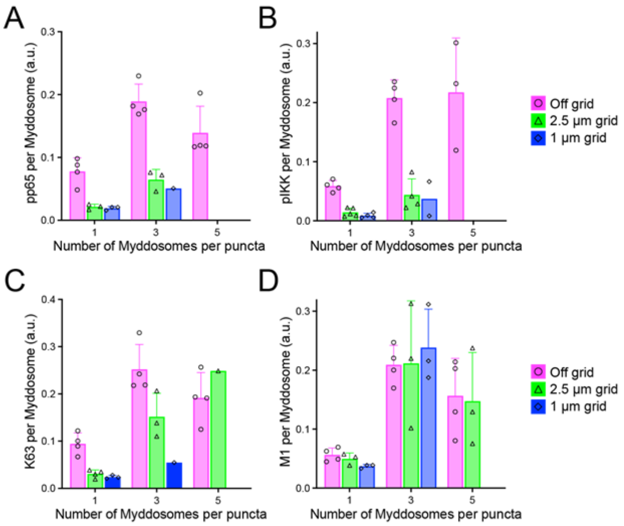

We find that the staining intensity per Myddosome complex increases by approximately 2-fold (or greater) when Myddosomes are organised into clusters. We observe this effect across all four signalling outputs measured, pp65, pIKK, K63-Ub and M1-Ub. One confusion the referee might have (that lead to the assumption that the staining intensity per Myddosome is invariant) is that the scatter plot data points presented in Figure 3A-B and Figure4A-B are in log scale. Thus the vast majority of single Myddosome complexes have very low staining intensity.

This new analysis suggests that the signalling output per Myddosome is greater when those complexes are organised into clusters. This analysis supports our conclusion that the spatial organisation of Myddosomes regulates signalling output. This analysis is included in Figure EV2. We thank the referee for suggesting this analysis that enhances the biological conclusions of the paper.

These criticisms are partially erased by the data presented in Figure 5 and Figure 6, Now it becomes obvious that there is a difference between off-grid and on-grid events.
I find the kinetics experiments especially elegant and the results are this time more black and white than the previous col-localization data. There is also data in the supplementary figures on dependence on IL-1 density and the effects are clearer for this part.

We thank the reviewer for this kind feedback. We agree that the live cell data supports the colocalization data, and supports the core biological conclusions of the manuscript.

The discussion is appropriate and overall the manuscript is well written.

We thank the reviewer for these kind comments

1st Revision - Editorial Decision 17th Jul 2023

Dear Marcus,

Thank you for the submission of your revised manuscript to EMBO reports. We have now received the full set of referee reports that is copied below.

As you will see, all referees are very positive about the study and support publication.

Browsing through the manuscript myself, I noticed a few editorial things that we need before we can proceed with the official acceptance of your study.

- Please provide up to 5 keywords.

- The manuscript sections are in the wrong order. Please consult our Guide to Authors for the correct order.

- Please add the heading 'Disclosure and competing interests statement' to the Conflict of interest paragraph.

- Please add headings 'Figure Legends' and 'Expanded View Figure Legends'.

- Please remove the Author Contributions from the manuscript file and make sure that the author contributions in our online submission system are correct and up-to-date. The information you specified in the system will be automatically retrieved and typeset into the article. You can enter additional information in the free text box provided, if you wish.

- Reference format: The first 10 authors should be listed, followed by et al. Surname then initials only.

- Figure callouts in the text: callouts to Fig EV2A-D, EV4A&B, Appendix Figs S2A-C, S3A-D are missing. Please correct the appendix callouts to 'Appendix Figure S#'.

- Appendix: Please add page numbers to the table of contents and remove the figure titles from the title page. Please correct the names to 'Appendix Figure S#'.

- Appendix figures: Please remove the scale bar size (numbers) from the images, if possible, and define the size of all scale bars in the legends.

- Appendix Figure S3: It appears that you show the source data for the blots shown in Appendix Figure S2. May I suggest changing the panel labels to (A) and (B) and then describe in the legend that (A) shows the uncropped blot for S2A (left and right blots) and (B) the raw data for S2B. Then the information is easier mapped to the Western blots shown in Fig. S2. Please also clearly state in the figure legend that the cropped blots are the same as those shown in Fig S2. Alternatively, you can upload the raw data as Source Data file, instead of displaying it in the Appendix, but I leave this up to you.

- Please remove all movie information from the manuscript file and provide the legends as simple README.txt files. Then ZIP each movie with its legend.

- Table EV1 should be uploaded individually using the file type 'Expanded View'.

- Please include a statement about blinding even if blinding was not done in the Methods section (see Author Checklist).

- I attach to this email a related manuscript file with comments by our data editors. Please address all comments and upload a revised file with tracked changes with your final manuscript submission.

- Finally, EMBO reports papers are accompanied online by A) a short (1-2 sentences) summary of the findings and their significance, B) 2-3 bullet points highlighting key results and C) a synopsis image that is 550x300-600 pixels large (width x height) in PNG for JPG format. You can either show a model or key data in the synopsis image. Please note that the size is rather small and that text needs to be readable at the final size. Please send us this information along with the revised manuscript.

- On a different note, I would like to alert you that EMBO Press offers a new format for a video-synopsis of work published with us, which essentially is a short, author-generated film explaining the core findings in hand drawings, and, as we believe, can be very useful to increase visibility of the work. This has proven to offer a nice opportunity for exposure i.p. for the first author(s) of the study.
Please see the following link for representative examples and their integration into the article web page:
https://www.embopress.org/video_synopses
https://www.embopress.org/doi/full/10.15252/embj.2019103932

Please let me know, should you be interested to engage in commissioning a similar video synopsis for your work. According operation instructions are available and intuitive.

We look forward to seeing a final version of your manuscript as soon as possible.

With kind regards,

Martina

Martina Rembold, PhD
Senior Editor
EMBO reports

****

Referee #1:

The authors have addressed my questions and added clarifying text and a supplemental schematic. I agree that the authors have reasonably covered the range possible interpretations and that it makes sense to address these questions in future studies. The responses to other reviewers points further strengthen and clarify this important study. The approach provides unique information to the inflammatory cytokine receptor field and will introduce this approach to groups of biologists who might otherwise not encounter it. Thus, it will be helpful to a broad audience of biologists.

Referee #2:

The authors have addressed all my comments

Referee #3:

The authors have not performed additional experiments to address the concerns of the reviewers but their modifications of the text may be considered sufficient. I am in favor of publication at this stage.

The authors have addressed all minor editorial requests.

2nd Revision - Editorial Decision 27th Jul 2023

Dr. Marcus Taylor
MPI for Infection Biology
Chariteplatz 1
Berlin, orcid|||||| 10117
Germany

Dear Marcus,

I am very pleased to accept your manuscript for publication in the next available issue of EMBO reports. Thank you for your contribution to our journal.

At the end of this email I include important information about how to proceed. Please ensure that you take the time to read the information and complete and return the necessary forms to allow us to publish your manuscript as quickly as possible.

As part of the EMBO publication's Transparent Editorial Process, EMBO reports publishes online a Review Process File to accompany accepted manuscripts. As you are aware, this File will be published in conjunction with your paper and will include the referee reports, your point-by-point response and all pertinent correspondence relating to the manuscript.

If you do NOT want this File to be published, please inform the editorial office within 2 days, if you have not done so already, otherwise the File will be published by default [contact: emboreports@embo.org]. If you do opt out, the Review Process File link will point to the following statement: "No Review Process File is available with this article, as the authors have chosen not to make the review process public in this case."

Thank you again for your contribution to EMBO reports and congratulations on a successful publication. Please consider us again in the future for your most exciting work.

Kind regards,

Martina

Martina Rembold, PhD
Senior Editor
EMBO reports

****

THINGS TO DO NOW:

Please note that you will be contacted by Wiley Author Services to complete licensing and payment information. The required 'Page Charges Authorization Form' is available here: https://www.embopress.org/pb-assets/embo-site/er_apc.pdf - please download and complete the form and return to embopressproduction@wiley.com

EMBO Press participates in many Publish and Read agreements that allow authors to publish Open Access with reduced/no publication charges. Check your eligibility: https://authorservices.wiley.com/author-resources/Journal-Authors/open-access/affiliation-policies-payments/index.html

You will receive proofs by e-mail approximately 2-3 weeks after all relevant files have been sent to our Production Office; you should return your corrections within 2 days of receiving the proofs.

Please inform us if there is likely to be any difficulty in reaching you at the above address at that time. Failure to meet our deadlines may result in a delay of publication, or publication without your corrections.

All further communications concerning your paper should quote reference number EMBOR-2023-57233V3 and be addressed to emboreports@wiley.com.

Should you be planning a Press Release on your article, please get in contact with emboreports@wiley.com as early as possible, in order to coordinate publication and release dates.

EMBO Press Author Checklist

Corresponding Author: Marcus J Taylor
Journal Submitted to: EMBO Reports
Manuscript Number: EMBOR-2023-57233V1

USEFUL LINKS FOR COMPLETING THIS FORM
The EMBO Journal - Author Guidelines
EMBO Reports - Author Guidelines
Molecular Systems Biology - Author Guidelines
EMBO Molecular Medicine - Author Guidelines

Reporting Checklist for Life Science Articles (updated January
This checklist is adapted from Materials Design Analysis Reporting (MDAR) Checklist for Authors. MDAR establishes a minimum set of requirements in transparent reporting in the life sciences (see Statement of Task: 10.31222/osf.io/9sm4x). Please follow the journal's guidelines in preparing your manuscript.
Please note that a copy of this checklist will be published alongside your article.

Abridged guidelines for figures
1. Data
The data shown in figures should satisfy the following conditions:
- the data were obtained and processed according to the field's best practice and are presented to reflect the results of the experiments in an accurate and unbiased manner.
- ideally, figure panels should include only measurements that are directly comparable to each other and obtained with the same assay.
- plots include clearly labeled error bars for independent experiments and sample sizes. Unless justified, error bars should not be shown for technical
- if n<5, the individual data points from each experiment should be plotted. Any statistical test employed should be justified.
- Source Data should be included to report the data underlying figures according to the guidelines set out in the authorship guidelines on Data

2. Captions
Each figure caption should contain the following information, for each panel where they are relevant:
- a specification of the experimental system investigated (eg cell line, species name).
- the assay(s) and method(s) used to carry out the reported observations and measurements.
- an explicit mention of the biological and chemical entity(ies) that are being measured.
- an explicit mention of the biological and chemical entity(ies) that are altered/varied/perturbed in a controlled manner.
- the exact sample size (n) for each experimental group/condition, given as a number, not a range;
- a description of the sample collection allowing the reader to understand whether the samples represent technical or biological replicates (including how many animals, litters, cultures, etc.).
- a statement of how many times the experiment shown was independently replicated in the laboratory.
- definitions of statistical methods and measures:
  - common tests, such as t-test (please specify whether paired vs. unpaired), simple χ2 tests, Wilcoxon and Mann-Whitney tests, can be unambiguously identified by name only, but more complex techniques should be described in the methods section;
  - are tests one-sided or two-sided?
  - are there adjustments for multiple comparisons?
  - exact statistical test results, e.g., P values = x but not P values < x;
  - definition of 'center values' as median or average;
  - definition of error bars as s.d. or s.e.m.

Please complete ALL of the questions below.
Select "Not Applicable" only when the requested information is not relevant for your study.

Materials

Newly Created Materials	Information included in the manuscript?	In which section is the information available? (Reagents and Tools Table, Materials and Methods, Figures, Data Availability Section)
New materials and reagents need to be available; do any restrictions apply?	Not Applicable	

Antibodies	Information included in the manuscript?	In which section is the information available? (Reagents and Tools Table, Materials and Methods, Figures, Data Availability Section)
For **antibodies** provide the following information:<br>- Commercial antibodies: RRID (if possible) or supplier name, catalogue number and or/clone number<br>- Non-commercial: RRID or citation	Yes	Reagent and Tools Table

DNA and RNA sequences	Information included in the manuscript?	In which section is the information available? (Reagents and Tools Table, Materials and Methods, Figures, Data Availability Section)
**Short novel DNA or RNA including primers, probes**: provide the sequences.	Yes	Reagents and Tools Table and Material and Methods section

Cell materials	Information included in the manuscript?	In which section is the information available? (Reagents and Tools Table, Materials and Methods, Figures, Data Availability Section)
**Cell lines:** Provide species information, strain. Provide accession number in repository **OR** supplier name, catalog number, clone number, and/**OR** RRID.	Yes	Reagents and Tools Table, Material and Methods section
**Primary cultures:** Provide species, strain, sex of origin, genetic modification status.	Not Applicable	
Report if the cell lines were recently **authenticated** (e.g., by STR profiling) and tested for mycoplasma contamination.	Yes	Mycoplasma testing done routinely, details provided in Materials and Methods

Experimental animals	Information included in the manuscript?	In which section is the information available? (Reagents and Tools Table, Materials and Methods, Figures, Data Availability Section)
**Laboratory animals or Model organisms:** Provide species, strain, sex, age, genetic modification status. Provide accession number in repository **OR** supplier name, catalog number, clone number, **OR** RRID.	Not Applicable	
**Animal observed in or captured from the field:** Provide species, sex, and age where possible**.**	Not Applicable	
Please detail **housing and husbandry conditions**.	Not Applicable	

Plants and microbes	Information included in the manuscript?	In which section is the information available? (Reagents and Tools Table, Materials and Methods, Figures, Data Availability Section)
**Plants:** provide species and strain, ecotype and cultivar where relevant, unique accession number if available, and source (including location for collected wild specimens).	Not Applicable	
**Microbes:** provide species and strain, unique accession number if available, and source.	Not Applicable	

Human research participants	Information included in the manuscript?	In which section is the information available? (Reagents and Tools Table, Materials and Methods, Figures, Data Availability Section)
If collected and within the bounds of privacy constraints report on age, sex and gender or ethnicity for all study participants.	Not Applicable	

Core facilities	Information included in the manuscript?	In which section is the information available? (Reagents and Tools Table, Materials and Methods, Figures, Data Availability Section)
If your work benefited from core facilities, was their service mentioned in the acknowledgments section?	Not Applicable	

Design

Study protocol	Information included in the manuscript?	In which section is the information available? (Reagents and Tools Table, Materials and Methods, Figures, Data Availability Section)
If study protocol has been **pre-registered, provide DOI in the manuscript**. For clinical trials, provide the trial registration number **OR** cite DOI.	Not Applicable	
Report the **clinical trial registration number** (at ClinicalTrials.gov or equivalent), where applicable.	Not Applicable	

Laboratory protocol	Information included in the manuscript?	In which section is the information available? (Reagents and Tools Table, Materials and Methods, Figures, Data Availability Section)
Provide DOI OR other citation details if **external detailed step-by-step protocols** are available.	Not Applicable	

Experimental study design and statistics	Information included in the manuscript?	In which section is the information available? (Reagents and Tools Table, Materials and Methods, Figures, Data Availability Section)
Include a statement about **sample size** estimate even if no statistical methods were used.	Not Applicable	
Were any steps taken to minimize the effects of subjective bias when allocating animals/samples to treatment (e.g. **randomization procedure**)? If yes, have they been described?	Not Applicable	
Include a statement about **blinding** even if no blinding was done.	Yes	
Describe **inclusion/exclusion criteria** if samples or animals were excluded from the analysis. Were the criteria pre-established?<br><br>If sample or data points were omitted from analysis, report if this was due to attrition or intentional exclusion and provide justification.	Not Applicable	
For every figure, are **statistical tests** justified as appropriate? Do the data meet the assumptions of the tests (e.g., normal distribution)? Describe any methods used to assess it. Is there an estimate of variation within each group of data? Is the variance similar between the groups that are being statistically compared?	Yes	

Sample definition and in-laboratory replication	Information included in the manuscript?	In which section is the information available? (Reagents and Tools Table, Materials and Methods, Figures, Data Availability Section)
In the figure legends: state number of times the experiment was **replicated** in laboratory.	Yes	In the figure legends
In the figure legends: define whether data describe **technical or biological replicates**.	Yes	In the figure legends

Ethics

Ethics	Information included in the manuscript?	In which section is the information available? (Reagents and Tools Table, Materials and Methods, Figures, Data Availability Section)
Studies involving **human participants**: State details of **authority granting ethics approval** (IRB or equivalent committee(s), provide reference number for approval.	Not Applicable	
Studies involving **human participants**: Include a statement confirming that **informed consent** was obtained from all subjects and that the experiments conformed to the principles set out in the WMA Declaration of Helsinki and the Department of Health and Human Services Belmont Report.	Not Applicable	
Studies involving **human participants:** For publication of **patient photos**, include a statement confirming that consent to publish was obtained.	Not Applicable	
Studies involving experimental **animals**: State details of **authority granting ethics approval** (IRB or equivalent committee(s), provide reference number for approval. Include a statement of compliance with ethical regulations.	Not Applicable	
Studies involving **specimen and field samples:** State if relevant **permits** obtained, provide details of authority approving study; if none were required, explain why.	Not Applicable	

Dual Use Research of Concern (DURC)	Information included in the manuscript?	In which section is the information available? (Reagents and Tools Table, Materials and Methods, Figures, Data Availability Section)
Could your study fall under dual use research restrictions? Please check biosecurity documents and list of **select agents and toxins** (CDC): https://www.selectagents.gov/sat/list.htm	Not Applicable	
If you used a select agent, is the security level of the lab appropriate and reported in the manuscript?	Not Applicable	
If a study is subject to dual use research of concern regulations, is the name of the **authority granting approval and reference number** for the regulatory approval provided in the manuscript?	Not Applicable	

Reporting

The MDAR framework recommends adoption of discipline-specific guidelines, established and endorsed through community initiatives. Journals have their own policy about requiring specific guidelines and recommendations to complement MDAR.

Adherence to community standards	Information included in the manuscript?	In which section is the information available? (Reagents and Tools Table, Materials and Methods, Figures, Data Availability Section)
State if relevant guidelines or checklists (e.g., **ICMJE, MIBBI, ARRIVE, PRISMA**) have been followed or provided.	Not Applicable	
For **tumor marker prognostic studies,** we recommend that you follow the **REMARK** reporting guidelines (see link list at top right). See author guidelines, under 'Reporting Guidelines'. Please confirm you have followed these guidelines.	Not Applicable	
For **phase II and III randomized controlled trials**, please refer to the **CONSORT** flow diagram (see link list at top right) and submit the CONSORT checklist (see link list at top right) with your submission. See author guidelines, under 'Reporting Guidelines'. Please confirm you have submitted this list.	Not Applicable	

Data Availability

Data availability	Information included in the manuscript?	In which section is the information available? (Reagents and Tools Table, Materials and Methods, Figures, Data Availability Section)
Have **primary datasets** been deposited according to the journal's guidelines (see 'Data Deposition' section) and the respective accession numbers provided in the Data Availability Section?	Yes	Imaging and numerical data sets provided in ZIP file uploaded with resubmission, in accordance with the journal guidelines
Were **human clinical and genomic datasets** deposited in a public access-controlled repository in accordance to ethical obligations to the patients and to the applicable consent agreement?	Not Applicable	
Are **computational models** that are central and integral to a study available without restrictions in a machine-readable form? Were the relevant accession numbers or links provided?	Not Applicable	
If publicly available data were reused, provide the respective **data citations in the reference list**.	Not Applicable	
